# Preparation of Lipophilic Derivatives of *para*-Aminosalicylic Acid for Antimicrobial Drug Design

**DOI:** 10.1021/acsomega.5c08092

**Published:** 2025-09-24

**Authors:** Michael J. Hearn, Alice K. Min

**Affiliations:** Department of Chemistry, Wellesley College, Wellesley, Massachusetts 02481, United States

## Abstract

Interference with folic acid metabolism in pathogenic micro-organisms is a productive approach for the design of new anti-infectives. Such materials out-compete the natural substrates, inhibiting folate synthetase. They prevent cellular replication of the pathogen and promote the health of the infected host. Investigations of *para*-aminosalicylic acid (PAS) and its congeners as antifolates have had a long and distinguished history, but much still remains to be learned about the specific roles of structural features leading to successful antimicrobial activity. The subject of this report is the preparation of a number of stable lipophilic derivatives of PAS, using the methods of synthetic organic chemistry. Our focus was on the development of a set of well-characterized structurally related materials that might be used as probes to clarify the particular role of lipophilicity in PAS antifolate drug action. The lipophilicities (*C*log *P*) of the new compounds in this cohort range in a graduated way from a low of approximately 1 to a high of nearly 11. We anticipate that this range of values could be of use in explorations of factors crucial to the antifolate drug efficacy of PAS and its derivatives. Biological assessments in vitro of the new compounds against *Mycobacterium tuberculosis* are provided, as one example of a pathogen-specific application.

## Graphical abstract

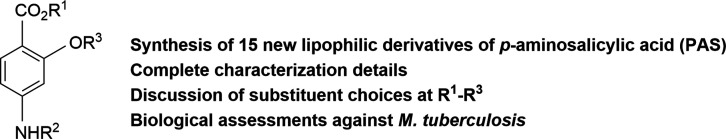

## Introduction

In many pathogenic micro-organisms, disruption of the folic acid metabolism is a productive tactic for the design of new anti-infectives. Folic acid antagonists may be structural analogs of folic acid itself or of its precursors. Such materials out-compete the natural substrates, inhibiting folate synthetase, thus preventing cellular replication of the pathogen and promoting the well-being of the infected host.^1–6^ The roles of *para*-aminosalicylic acid and its congeners as antifolates have long been of interest,^7,8^ but much still remains to be learned about the specific roles of structural features leading to successful antimicrobial activity. As only two examples of current widely used drugs in line with this approach, applied in both human and veterinary medicine, we may cite *para*-aminosalicylic acid (PAS, Figure 1, R^1^ = R^2^ = R^3^ = H) in the chemotherapy of drug-resistant tuberculosis in people^9^ and the use of Ethopabate (Figure 1, R^1^ = H, R^2^ = COCH_3_, R^3^ = CH_2_CH_3_) against *Eimeria* species in the treatment of avian coccidiosis.^10^ Drug action is a complex process, in which a number of important factors play significant roles. Each may make a contribution, influencing drug absorption, metabolism, and ultimate fit within an active site, to name only a few. Among these significant factors is the characteristic of lipophilicity. The subject of this report is the preparation of a number of stable lipophilic derivatives of PAS, using the methods of synthetic organic chemistry, and the use of these derivatives in *Mycobacterium tuberculosis*.

**Figure 1:**
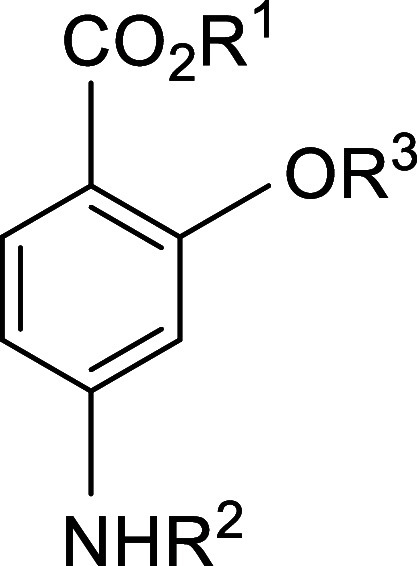
*Para*-Aminosalicylic acid (PAS) and its derivatives.

## Results and Discussion

In the search for promising anti-infectives, the functional groups of PAS provide rich opportunities for derivatization, each of which has its own basis for exploration. Significant chemical issues include: (1) at the carboxyl group, the promotion of chemical stability for robust drugs; (2) at the amino group, addressing the issue of the drug-deactivating effects of endogenous enzymes in both host and pathogen; and (3) at the phenolic unit, the management of drug lipophilicity to promote drug distribution and penetration. Because they motivated our overall design choices for the new compounds, we briefly discuss each of these chemical issues below; however, the emphasis of the work in this study is on the manipulation of lipophilicity through substitution at R^3^ (Figure 1).

(1) With regard to the issue of chemical stability, under warm and humid conditions PAS is subject to decarboxylation. This process degrades the drug and forms the human hepatotoxin *meta*-aminophenol, a process on which we^11^ and others^12^ have previously reported. This places some limits on the use of PAS in the tropics. To counter this deleterious effect, a number of esters of PAS have been studied for their stability and antimicrobial properties. Among these esters, methyl 4-aminosalicylate (MAS, Figure 1, R^1^ = CH_3_, R^2^ = R^3^ = H)^13,14^ is a stable crystalline solid which does not require a cold chain or anhydrous conditions for transport, storage or use. The material is widely available as an inexpensive bulk chemical and represents a suitable departure point for our synthesis efforts.
(2) Concerning enzymatic deactivation, drugs containing the arylamino moiety, including PAS, are susceptible to deactivation by arylamine *N*-acetyltransferases (NATs), enzymes that are found in both pathogens and their hosts.^15,16^ In human beings, NATs form part of the normal processes of xenobiotic transformation, whereby compounds outside the body’s normal biochemistry, including arylamine drugs, are converted in the liver to acetamides that may be more readily excreted. Drugs can be metabolized before reaching effective antimicrobial concentrations in circulation in the host. In some disease-causing organisms, NATs also form part of the pathogen’s own defense system against anti-infective drugs.^17^ The metabolic products have lower antimicrobial activities, compromising their role in effective chemotherapy.^18^ One prospective strategy for the improvement of antimicrobial character emphasizes chemically blocking the deactivating effects of NATs through changes in drug structure at the amine group. Previous work in our laboratory and others has suggested that a number of structural units may be suitable as blocking agents,^19–22^ including acyl groups other than acetyl.^23,24^ The action of NATs may be countered by blocking the free amino group of PAS with a suitable function. In the present study, we chose the propionyl group. Propionylation of MAS thus gave us the precursor propionamido compound (Figure 1, R^1^ = CH_3_, R^2^ = COCH_2_CH_3_, R^3^ = H, **I**, PAC) that was stable, easily purified and amenable to preparation at gram scale (see Methods Section). This enabled the preparation of the desired lipophilic derivatives.
(3) In regard to drug penetration, molecules with higher lipophilicities may be able to more easily cross biological membranes, permeating by diffusion the hydrocarbon interior of the lipid bilayer of a pathogen’s cell wall. However, the thickness of the bilayer itself may result in a reduction of the diffusion process, and this may be only one of the presumed factors limiting penetration. This situation underscores the potential usefulness of an array of PAS derivatives possessing a wide range of lipophilicities. The availability of such an array would facilitate biological assessments of antimicrobial potencies as a function of compound lipophilicity and foster a better understanding of the role of lipophilicity in antifolate drug action.^2,25^ Some studies have suggested that lipophilic derivatives of chemotherapeutic agents are more active against bacterial pathogens than their less-lipophilic counterparts,^25–27^ and there is some evidence that higher lipophilicity may be a contributing factor for increased oral biovailability.^28,29^ Nonetheless, it is important to bear in mind the multifactorial nature of drug action, and there are dangers in driving drug design solely on this property.^30^

In the present work, our focus was on the manipulation of compound lipophilicity by modifying the phenolic function through nucleophilic substitution. This allowed for the introduction of a considerable variety of structural units. Given the considerations reviewed above, PAC **I** was prepared by the selective reaction of MAS using one equivalent of propionic anhydride in refluxing tetrahydrofuran (Scheme 1). The material was purified by a procedure which did not require chromatography or recrystallization, and PAC could be stored for months without degradation.

**Scheme 1:**
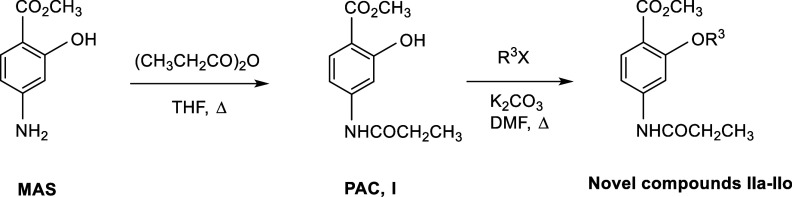
Preparation of Novel *para*-Aminosalicylic Acid Derivatives **IIa**–**IIo**

In the event, PAC **I** served as a convenient precursor for the preparation of the desired stable lipophilic derivatives **II** (Scheme 1). The reaction of **I** with 1.5 equiv of suitable alkyl or arylalkyl halides generated the target compounds **II** as crystalline solids. Analytically pure compounds were obtained simply by washing with ether. In a representative example, treatment of **I** with 4-fluorobenzyl bromide and potassium carbonate in warm DMF gave novel **IIa** (Scheme 1, R^3^ = 4-FC_6_H_4_CH_2_), in 98% yield, fully characterized as noted in the Methods Section. At the bench, the conversion of **I** to **IIa** was conveniently monitored by the in-growth of the very strong C–O–C ether band near 1260 cm^–1^ in the infrared spectrum, and the observation of this characteristic band was a rapid diagnostic for the routine determination of our other synthetic reactions.

The preparations of the 15 new compounds **IIa**–**IIo** are summarized in Table 1, and complete characterization details are provided in the Methods Section. Copies of all of the original spectra (FT-IR, ^1^H NMR, ^13^C NMR) are shown in the Supporting Information, and the data were consistent with the results of elemental analysis and HRMS. We would like to comment on the usefulness and reliability of the ^13^C NMR chemical shift values for structure determination in this set of compounds **II**. There were noteworthy consistencies in the values for the ester carbonyl (δ 173), the amide carbonyl (δ 165), the aromatic carbon bearing the ether oxygen (δ 160), and the aromatic carbon bearing the propionamido function (δ 144). The values have been tabulated in the Supporting Information for comparison.

**Table 1:**
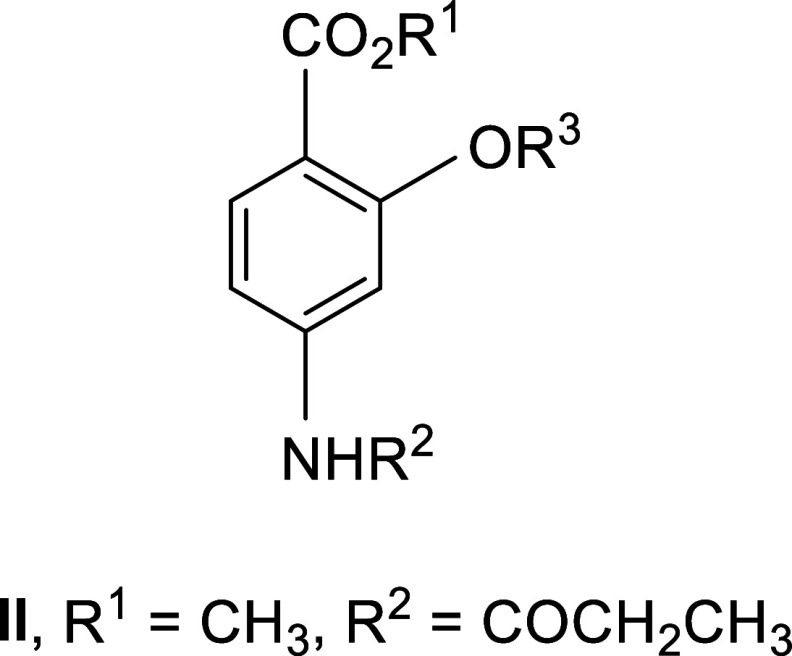
Preparation of Novel *para*-Aminosalicylic Acid Derivatives for Antimicrobial Drug Design

entry	compound **II**, R^3^	formula	mol. wt	yield	mp
1	**IIa**, CH_2_4–FC_6_H_4_	C_18_H_18_NO_4_F	331 g/mol	98%	111–113 °C
2	**IIb**, CH_2_2–C_10_H_7_	C_22_H_21_NO_4_	363	11^a^	122–123
3	**IIc**, *n*-C_7_H_15_	C_18_H_27_NO_4_	321	83	64–66
4	**IId**, *n*-C_6_H_13_	C_17_H_25_NO_4_	307	76	59–61
5	**IIe**, *n*-C_5_H_11_	C_16_H_23_NO_4_	293	68	62–63
6	**IIf**, *n*-C_4_H_9_	C_15_H_21_NO_4_	279	82	87
7	**IIg**, *i*-C_5_H_11_	C_16_H_23_NO_4_	293	68	70
8	**IIh**, CH_2_3–CH_3_C_6_H_4_	C_19_H_21_NO_4_	327	100	95–98
9	**IIi**, CH_2_4–CH_3_C_6_H_4_	C_19_H_21_NO_4_	327	65	118
10	**IIj**, CH_2_3–CH_3_OC_6_H_4_	C_19_H_21_NO_5_	343	90	87–89
11	**IIk**, CH_2_C_6_H_5_	C_18_H_19_NO_4_	313	70	105
12	**IIl**, (CH_2_)_11_CH_3_	C_23_H_37_NO_4_	391	88	45–46
13	**IIm**, (CH_2_)_13_CH_3_	C_25_H_41_NO_4_	419	97	50–51
14	**IIn**, (CH_2_)_15_CH_3_	C_27_H_45_NO_4_	447	99	55–57
15	**IIo**, (CH_2_)_17_CH_3_	C_29_H_49_NO_4_	475	85	65–67

a See Methods Section.

Table 2 lists the calculated values of the lipophilicities of the compounds (*C*log *P*) in this cohort. These range in a graduated way from a low of approximately 1 for PAS itself to a high of nearly 11 for compound **IIo**. In the synthesis laboratory, this range was readily obtained by etherification of the phenolic unit, via the progressive increase in chain length of alkyl components (**IIc**–**IIg**, **IIl**–**IIo**) or via substituent effects in arylalkyl groups (**IIa**, **IIb**, **IIh**–**IIk**).

**Table 2:** *C*log *P* Values

entry	compound	*C*log *P* ^a^
1	PAS	1.056
2	MAS	1.261
3	PAC	2.203
4	**IIa**	3.849
5	**IIb**	4.880
6	**IIc**	5.112
7	**IId**	4.583
8	**IIe**	4.054
9	**IIf**	3.525
10	**IIg**	3.924
11	**IIh**	4.205
12	**IIi**	4.205
13	**IIj**	3.625
14	**IIk**	3.706
15	**IIl**	7.757
16	**IIm**	8.815
17	**IIn**	9.873
18	**IIo**	10.931
19	ethopabate	1.94

a *C*log *P* values calculated using ChemDraw Ultra (version 11.0, Cambridgesoft, Cambridge, MA USA).

The interaction of antifolate drugs with pathogens is a complex and multifactorial process.^2^ Because the folate pathway may differ from one micro-organism to another, the exploration of the role of lipophilicity in antifolate action should be done on a pathogen-specific basis. In our work, the compounds were examined for their activities against *M. tuberculosis*, a microbe well-known for its waxy cell wall and for its hardiness toward cell wall penetration by antimycobacterial agents. The activities were measured in vitro as the minimum inhibitory concentrations needed to prevent at least 90% of the growth of the bacteria (MIC_90_). The activity data are shown in Table 3 and were obtained as noted in the Methods Section, according to well-documented protocols.^31,32^

**Table 3:** Activities in vitro Against *M. tuberculosis* ^a^

entry	compound	compound **II**, R^3^	MIC_90_ (μg/mL)
1	**I**, PAS		8
2	**IIa**	CH_2_4-FC_6_H_4_	36.8
3	**IIb**	CH_2_2–C_10_H_7_	>50
4	**IIc**	*n*-C_7_H_15_	29.1
5	**IId**	*n*-C_6_H_13_	16.7
6	**IIe**	*n*-C_5_H_11_	71.9
7	**IIf**	*n*-C_4_H_9_	>100
8	**IIg**	*i*-C_5_H_11_	44.1
9	**IIh**	CH_2_3–CH_3_C_6_H_4_	74.3
10	**IIi**	CH_2_4–CH_3_C_6_H_4_	87.1
11	**IIj**	CH_2_3–CH_3_OC_6_H_4_	>100
12	**IIk**	CH_2_C_6_H_5_	>50
13	**IIl**	(CH_2_)_11_CH_3_	34.9
14	**IIm**	(CH_2_)_13_CH_3_	>50
15	**IIn**	(CH_2_)_15_CH_3_	not determined
16	**IIo**	(CH_2_)_17_CH_3_	>100

a MIC_90_ values for positive controls: Isoniazid 0.03 μg/mL, PAS 0.015 μg/mL.^31,32^

Focusing on the effects of lipophilicity, it might be hypothesized that compounds with greater *C*log *P* values would have greater activities. Considering compounds with shorter straight-chain alkyl substituents at the phenolic unit (C_4_–C_7_), the data suggest that compounds with higher *C*log *P* values (**IIc**–**IIe**) are indeed somewhat more active than the case with a lower *C*log *P* value (**IIf**). On the other hand, for compounds with longer straight-chain alkyl substituents, such as **IIo**, the lipophilicity is very much higher, but the antibacterial activity is lower. With the latter molecules, there is a substantial increase in size, and this increase in size may over-ride lipophilicity in the ultimate fit of the antimicrobial agent within the active site of the relevant folate enzyme, a situation which has precedence in the literature.^2,25^ In line with this, we note that the calculated Connolly accessible area of **IIo** (973 Å^2^) is significantly greater than those of shorter-chain materials such as **IIc** (640 Å^2^). Compounds derived from substitutions at the phenolic unit with arylalkyl moieties displayed a range of MIC_90_ values, as might be anticipated from effects additional to lipophilic character, such as size or hydrogen bonding. In general, the antitubercular activities of the new compounds are modest. Comparing the overall activities of compounds **II** versus **I**, it seems clear that, for *M. tuberculosis*, the presence of a free phenolic moiety enhances activity. However, this observation may not necessarily apply to other pathogens, in which the folate pathway may differ.

## Conclusions

In this report we have presented in detail the preparation and characterization of stable lipophilic derivatives of *para*-aminosalicylic acid. The rationale for our compound design choices has been discussed. These novel compounds could serve as useful probes of the disruption of the folic acid metabolism of pathogenic organisms and help to clarify the role of lipophilicity as a factor in the antifolate action of PAS and its analogs. Biological assessments in vitro of the new compounds against *M. tuberculosis* are provided, as one example of a pathogen-specific application. The results are consistent with lipophilicity as a contributing characteristic in antifolate drug action, but they point out the need to consider other factors as well, including size.

## Methods

Reagents were obtained and used without further purification from the following commercial sources: Aldrich Chemical Co., Milwaukee, Wisconsin, USA; Lancaster Synthesis Incorporated, Windham, New Hampshire, USA; and Trans World Chemicals, Incorporated, Rockville, Maryland, USA. Reactions were carried out in a shielded fume hood on 10–15 mmol scale in a 100 mL round-bottom flask fitted for reflux with a magnetic stirrer and heating mantle. *Safety notes*: standard good-practice techniques were followed, including the routine use of safety glasses, protective laboratory coat, gloves and good ventilation. Any reactions for the preparation of compounds with higher nitrogen or oxygen content were done with due caution. No specific safety problems were noted with the methods given below.

Melting points (mp, °C) were taken in open capillary tubes using a Mel-Temp apparatus (Laboratory Devices, Cambridge, Massachusetts, USA) and have been corrected. Elemental analyses were carried out by Galbraith Laboratories, Knoxville, Tennessee, USA. Infrared spectra (FT-IR) were recorded on a PerkinElmer Spectrum One Fourier Transform spectrophotometer fitted with a universal attenuated total reflectance sampling accessory, reported in wavenumbers (ν, cm^–1^). Nuclear magnetic resonance (NMR) spectra were taken on a Bruker 300 Fourier transform instrument in dimethyl sulfoxide-*d*_6_ (DMSO), recorded at 300 MHz (^1^H NMR) or 75 MHz (^13^C NMR), and are reported in parts per million delta (δ) downfield from internal tetramethylsilane as reference. In the ^1^H NMR spectra, common aliphatic and aromatic coupling constants (*J*) were in the expected range (5–9 Hz) and are not specifically enumerated. High resolution mass spectra (HRMS, fast atom bombardment method) and low resolution mass spectra were determined at the National Institutes of Health Mass Spectrometry Facility at Michigan State University, East Lansing, Michigan, USA. No attempts were made to optimize yields. Connolly characteristics of compounds **IIo** and **IIc** were obtained using the QSAR properties function of ChemDraw and Chem 3D.

Activities of antimicrobial agents in vitro were determined through the well-documented protocols^31^ of the Tuberculosis Antimicrobial Acquisition and Coordinating Facility, a unit of the National Institutes of Health, and through the courtesy of Dr. Michael Cynamon, Veterans Affairs Medical Center, Syracuse, New York, by established procedures.^32^

### Methyl 4-Propionamidosalicylate (**I**, PAC)

Methyl 4-aminosalicylate (MAS, 1.67 g, 10.0 mmol) was dissolved in tetrahydrofuran (THF, 15 mL) and brought to reflux in the apparatus described above. To this warm rapidly stirring homogeneous mixture was added dropwise a solution of propionic anhydride (1.30 g, 10.0 mmol) in THF (5 mL). The propionic anhydride was washed in with further THF (5 mL). Reflux was continued for 2.5 h. The mixture was then cooled to room temperature, and the THF was evaporated in a gentle stream of air until the reaction product solidified. The solid was dissolved in anhydrous ethyl ether (15 mL) and refluxed for 10 min. The contents of the flask were cooled to room temperature and the resulting solid filtered. The solid was washed on the filter with ether (3 × 3 mL) and dried, giving the analytically pure compound. The product was a pale beige solid; over many preparations the yield ranged from 50 to 60%, averaging approximately 55%, mp 175–176 °C; FT-IR (ATR, cm^–1^): 3303, 2955, 1671, 1262, 1190; H NMR (300 MHz, DMSO-*d*_6_, δ): 10.5 (s, 1H), 10.1 (s, 1H), 7.6 (d, 1H), 7.3 (s, 1H), 7.0 (d, 1H), 3.8 (s, 3H), 2.2 (q, 2H), 1.0 (t, 3H); C NMR (75 MHz, DMSO-*d*_6_, δ): 173, 169, 161, 146, 131, 110, 107, 106, 52, 30, 9; HRMS (*m*/*z* MH+) calcd for C_11_H_14_NO_4_, 224.09228; found, 224.09220.

Anal. Calcd for C_11_H_13_NO_4_: C, 59.19; H, 5.87. Found: C, 59.14; H, 5.97.

### General Procedure for the Preparation of Compounds **IIa**–**IIp**

Methyl 4-propionamidosalicylate (PAC, **I**, 2.09 g, 9.37 mmol) was mixed with potassium carbonate (2.76 g, 20.0 mmol) and dimethylformamide (DMF, 10 mL). To the stirring mixture was added in several portions a solution of the appropriate alkyl or arylalkyl halide (13.7 mmol) dissolved in DMF (5 mL). The halide was washed in with a further portion of DMF (5 mL). The mixture was warmed at 50–60 °C for 18 h. Heating was stopped, and the mixture attained room temperature and was then quenched in an excess of ice-cold water (100 mL). Ethyl acetate (75 mL) was added and the entire mixture was transferred to a 250 mL separatory funnel. The aqueous layer was extracted, and the organic layer was washed with cold water (4 × 25 mL) and dried over anhydrous magnesium sulfate. The solution was then filtered and evaporated to form a solid product. The analytical sample was obtained by washing the solid with several portions of anhydrous ethyl ether.

### Methyl 2-(4-Fluorobenzyloxy)-4-propionamidosalicylcate (**IIa**)

Yield 3.03 g (98%); mp 111–113 °C; FT-IR (ATR, cm^–1^): 3258, 3192, 2954, 1699, 1670, 1600, 1537, 1509, 1402, 1260, 1220, 1132, 1010, 925, 921, 758; H NMR (300 MHz, DMSO-*d*_6_, δ): 10.2 (s, 1H), 7.7–7.2 (m, 7H), 5.2 (s, 2H), 3.8 (s, 3H), 2.3 (q, 2H), 1.1 (t, 3H); C NMR (75 MHz, DMSO-*d*_6_, δ): 173, 165, 163, 160, 158, 144, 133, 129, 115, 114, 111, 104, 69, 52, 30, 9. HRMS (*m*/*z* MH+): calcd for C_18_H_19_NO_4_F, 332.1298; found, 332.1295.

Anal. Calcd for C_18_H_18_NO_4_F: C, 65.24; H, 5.47. Found: C, 65.12; H, 5.36.

### Methyl 2-(2-Naphthylmethoxy)-4-propionamidosalicylate (**IIb**)

Yield 0.37 g (11%); mp 122–123 °C; FT-IR (ATR, cm^–1^): 3348, 3048, 1681, 1595, 1509, 1436, 1406, 1259, 1207, 1136, 1023, 855, 801, 739; H NMR (300 MHz, DMSO-*d*_6_, δ): 10.3 (s, 1H), 8.3–7.3 (m, 10H), 5.5 (s, 2H), 4.08 (s, 3H), 2.5 (q, 2H), 1.2 (t, 3H); C NMR (75 MHz, DMSO-*d*_6_, δ): 173, 165, 159, 144, 134, 133, 132.45, 132.30, 127.93, 127.74, 127.60, 126.30, 126.02, 125.47, 125.07, 114, 111, 104, 70, 52, 30, 9; HRMS (*m*/*z* MH+): calcd for C_22_H_22_NO_4_, 364.1549; found, 364.1544. We believe that the low yield in this preparation was due to the considerable solubility of the product in DMF and that exhaustive extraction with further portions of ethyl acetate would likely improve recovery.

Anal. Calcd for C_22_H_21_NO_4_: C, 72.71; H, 5.82. Found: C, 72.31; H, 5.64.

### Methyl 2-(*n*-Heptyloxy)-4-propionamidosalicylate (**IIc**)

Yield 2.50 g (83%); mp 64–66 °C; FT-IR (ATR, cm^–1^): 3311, 2943, 2869, 1683, 1663, 1584, 1537, 1421, 1291, 1267, 1229, 1139, 1019, 857, 782, 759; H NMR (300 MHz, DMSO-*d*_6_, δ): 10.3 (s, 1H), 7.9 (d, 1H), 7.7 (s, 1H), 7.4 (dd, 1H), 4.1 (t, 2H), 3.9 (s, 3H), 2.5 (q, 2H), 1.9 (m, 2H), 1.6 (m, 2H), 1.4 (m, 6H), 1.2 (t, 3H), 1.0 (t, 3H); C NMR (75 MHz, DMSO-*d*_6_, δ): 173, 165, 159, 144, 132, 114, 110, 103, 68, 51, 31, 30, 29, 28, 25, 22, 14, 9; HRMS (*m*/*z* MH+): calcd for C_18_H_28_NO_4_, 322.2018; found, 322.2016.

Anal. Calcd for C_18_H_27_NO_4_: C, 67.30; H, 8.47. Found: C, 67.08; H, 8.21.

### Methyl 2-(*n*-Hexyloxy)-4-propionamidosalicylate (**IId**)

Yield 2.18 g (76%); mp 59–61 °C; FT-IR (ATR, cm^–1^): 3342, 2936, 2870, 1703, 1688, 1583, 1528, 1417, 1329, 1288, 1235, 1209, 1148, 1090, 1039, 927, 860, 823, 777, 702; H NMR (300 MHz, DMSO-*d*_6_, δ): 10.2 (s, 1H), 7.7 (d, 1H), 7.6 (s, 1H), 7.2 (dd, 1H), 4.0 (t, 2H), 3.8 (s, 3H), 2.4 (q, 2H), 1.8 (m, 2H), 1.5 (m, 2H), 1.4 (m, 4H), 1.1 (t, 3H), 0.9 (t, 3H); C NMR (75 MHz, DMSO-*d*_6_, δ): 173, 166, 159, 145, 132, 114, 110, 104, 68, 52, 31, 30, 29, 25, 22, 14, 10; HRMS (*m*/*z* MH+): calcd for C_17_H_26_NO_4_, 308.1861; found: 308.1863.

Anal. Calcd for C_17_H_25_NO_4_: C, 66.42; H, 8.19. Found: C, 66.11; H, 8.14.

### Methyl 2-(*n*-Pentyloxy)-4-propionamidosalicylate (**IIe**)

Yield 2.71 g (68%); mp 62–63 °C; FT-IR (ATR, cm^–1^): 3340, 2937, 2868, 1704, 1689, 1584, 1530, 1417, 1331, 1289, 1233, 1195, 1153, 1090, 1025, 857, 778, 701; H NMR (300 MHz, DMSO-*d*_6_, δ): 10.1 (s, 1H), 7.6 (d, 1H), 7.5 (s, 1H), 7.2 (dd, 1H), 4.0 (t, 2H), 3.7 (s, 3H), 2.4 (q, 2H), 1.7 (m, 2H), 1.4 (m, 4H), 1.1 (t, 3H), 0.9 (t, 3H); C NMR (75 MHz, DMSO-*d*_6_, δ): 173, 165, 159, 144, 132, 114, 110, 103, 68, 51, 30, 28, 27, 22, 14, 9; HRMS (*m*/*z* MH+): calcd for C_16_H_24_NO_4_, 294.1705; found, 294.1701.

Anal. Calcd for C_16_H_23_NO_4_: C, 65.50; H, 7.90. Found: C, 65.26; H, 7.91.

### Methyl 2-(*n*-Butyloxy)-4-propionamidosalicylate (**IIf**)

Yield (82%); mp °C; FT-IR (ATR, cm^–1^): 3259, 2940, 1717, 1665, 1591, 1538, 1465, 1413, 1241, 1222, 1147, 1071, 843, 780; H NMR (300 MHz, DMSO-*d*_6_, δ): 10.1 (s, 1H), 7.7 (d, 1H), 7.5 (s, 1H), 7.2 (d, 1H), 4.0 (t, 2H), 3.8 (s, 3H), 2.4 (q, 2H), 1.7 (m, 2H), 1.5 (m, 2H), 1.1 (t, 3H), 0.9 (t, 3H); C NMR (75 MHz, DMSO-*d*_6_, δ): 173, 165, 159, 144, 132, 114, 110, 103, 68, 51, 31, 30, 19, 14, 9; HRMS (*m*/*z* MH+): calcd for C_15_H_22_NO_4_, 280.1548; found, 280.1548.

Anal. Calcd for C_15_H_21_NO_4_: C, 64.49; H, 7.58. Found: C, 64.11; H, 7.40.

### Methyl 2-((3-Methyl)­butyloxy)-4-propionamidosalicylate (**IIg**)

Yield (68%); mp 70 °C; FT-IR (ATR, cm^–1^): 3254, 2949, 1702, 1666, 1598, 1535, 1434, 1404, 1314, 1262, 1227, 1131, 1097, 1047, 991, 923, 840, 760; H NMR (300 MHz, DMSO-*d*_6_, δ): 10.5 (s, 1H), 7.7 (d, 1H), 7.5 (s, 1H), 7.2 (d, 1H), 4.0 (t, 2H), 3.7 (s, 3H), 2.4 (q, 2H), 1.9 (m, 1H), 1.6 (q, 2H), 1.1 (t, 3H), 0.9 (2t, 6H); C NMR (75 MHz, DMSO-*d*_6_, δ): 173, 165, 159, 144, 132, 114, 110, 103, 66, 51, 37, 30, 24, 22, 9; HRMS (*m*/*z* MH+): calcd for C_16_H_24_NO_4_, 294.1705; found, 294.1703.

Anal. Calcd for C_16_H_23_NO_4_: C, 65.50; H, 7.90. Found: C, 65.22; H, 7.83.

### Methyl 2-(3-Methylbenzyloxy)-4-propionamidosalicylate (**IIh**)

Yield (100%); mp 95–98 °C; FT-IR (ATR, cm^–1^): 3355, 2939, 1686, 1530, 1510, 1439, 1406, 1277, 1206, 1135, 1019, 841, 778, 696; H NMR (300 MHz, DMSO-*d*_6_, δ): 10.0 (s, 1H), 7.5 (d, 1H), 7.4 (s, 1H), 7.0 (m, 5H), 4.9 (s, 2H), 3.6 (s, 3H), 2.1 (m, 5H), 0.9 (t, 3H); C NMR (75 MHz, DMSO-*d*_6_, δ): 173, 166, 159, 144, 137.38, 136.65, 132, 128.22, 128.19, 127.46, 123.95, 114, 110, 104, 69, 52, 30, 21, 9; HRMS (*m*/*z* MH+): calcd for C_19_H_22_NO_4_, 328.1549; found, 328.1544.

Anal. Calcd for C_19_H_21_NO_4_: C, 69.71; H, 6.47. Found: C, 69.31; H, 6.65.

### Methyl 2-(4-Methylbenzyloxy)-4-propionamidosalicylate (**IIi**)

Yield (65%); mp 118 °C; FT-IR (ATR, cm^–1^): 3311, 2918, 2974, 1729, 1664, 1603, 1532, 1508, 1397, 1247, 1222, 1184, 1141, 1087, 1019, 913, 843, 802, 713; H NMR (300 MHz, DMSO-*d*_6_, δ): 10.2 (s, 1H), 7.7 (d, 1H), 7.6 (s, 1H), 7.4 (d, 2H), 7.2 (d, 3H), 5.1 (s, 2H), 3.8 (s, 3H), 2.3 (m, 5H), 1.1 (t, 3H); C NMR (75 MHz, DMSO-*d*_6_, δ): 173, 165, 159, 144, 137, 134, 132, 129, 127, 116, 110, 104, 69, 51, 30, 21, 9; HRMS (*m*/*z* MH+): calcd for C_19_H_22_NO_4_, 328.1549; found, 328.1557.

Anal. Calcd for C_19_H_21_NO_4_: C, 69.71; H, 6.47. Found: C, 69.97; H, 6.69.

### Methyl 2-(3-Methoxybenzyloxy)-4-propionamidosalicylate (**IIj**)

Yield (90%); mp 87–89 °C; FT-IR (ATR, cm^–1^): 3352, 2939, 2838, 1719, 1682, 1672, 1587, 1530, 1408, 1380, 1290, 1227, 1149, 1085, 1076, 1046, 1024, 867, 852, 775, 690; H NMR (300 MHz, DMSO-*d*_6_, δ): 10.4 (s, 1H), 8.0 (d, 1H), 7.9 (s, 1H), 7.5 (t, 1H), 7.4 (m, 3H), 7.1 (dd, 1H), 5.3 (s, 2H), 4.0 (s, 6H), 2.6 (q, 2H), 1.3 (t, 3H). ; C NMR (75 MHz, DMSO-*d*_6_, δ): 173, 165, 159, 158, 144, 138, 132, 129, 119, 114, 113, 112, 110, 104, 69, 55, 52, 30, 9; HRMS (*m*/*z* MH+): calcd for C_19_H_22_NO_4_, 344.1498; found, 344.1501.

Anal. Calcd for C_19_H_21_NO_4_: C, 66.46; H, 6.16. Found: C, 66.61; H, 6.05.

### Methyl 2-(Benzyloxy)-4-propionamidosalicylate (**IIk**)

Yield (70%); mp 105 °C; FT-IR (ATR, cm^–1^): 3306, 2953, 2877, 1721, 1699, 1665, 1603, 1532, 1509, 1434, 1401, 1380, 1246, 1223, 1185, 1140, 1079, 1004, 843, 735, 695; H NMR (300 MHz, DMSO-*d*_6_, δ): 10.2 (s, 1H), 7.5 (m, 8H), 5.2 (s, 2H), 3.8 (s, 3H), 2.3 (q, 2H), 1.2 (t, 3H); C NMR (75 MHz, DMSO-*d*_6_, δ): 173, 165, 159, 144, 137, 132, 128.31, 127.57, 126.84, 114, 110, 104, 69, 52, 30, 9; HRMS (*m*/*z* MH+): calcd for C_18_H_20_NO_4_, 314.1392; found, 314.1389.

Anal. Calcd for C_18_H_29_NO_4_: C, 68.94; H, 6.11. Found: C, 69.13; H, 6.08.

### Methyl 2-(Dodecyloxy)-4-propionamidosalicylate (**IIl**)

Yield (88%); mp 45–46 °C; FT-IR (ATR, cm^–1^): 3304, 2915, 2849, 1730, 1699, 1663, 1607, 1546, 1509, 1470, 1434, 1406, 1390, 1242, 1222, 1186, 1141, 1079, 842, 737; H NMR (300 MHz, DMSO-*d*_6_, δ): 10.1 (s, 1H), 7.7 (d, 1H), 7.6 (s, 1H), 7.2 (d, 1H), 4.0 (t, 2H), 3.8 (s, 3H), 2.4 (q, 2H), 1.7­(m, 2H), 1.3 (m, 18H), 1.1 (t, 3H), 0.9 (t, 3H); C NMR (75 MHz, DMSO-*d*_6_, δ): 173, 165, 159, 144, 132, 114, 110, 103, 68, 51, 32.51, 31.71, 30, 29, 28.94, 28.69, 28.63, 28.50, 25, 22, 14, 9, coincident chemical shifts very likely; HRMS (*m*/*z* MH+): calcd for C_23_H_38_NO_4_, 392.2799; found, 392.2801.

Anal. Calcd for C_23_H_37_NO_4_: C, 70.55; H, 9.52. Found: C, 70.50; H, 9.55.

### Methyl 2-(Tetradecanyloxy)-4-propionamidosalicylate (**IIm**)

Yield (97%); mp 50–51 °C; FT-IR (ATR, cm^–1^): 3302, 2914, 2849, 1731, 1698, 1664, 1607, 1546, 1509, 1470, 1434, 1406, 1248, 1224, 1186, 1141, 1079, 842, 737, 719; H NMR (300 MHz, DMSO-*d*_6_, δ): 9.9 (s, 1H), 7.5 (d, 1H), 7.3 (s, 1H), 7.0 (d, 1H), 3.8 (t, 3H), 3.5 (s, 3H), 2.1 (q, 2H), 1.5 (m, 2H), 1.2 (m, 2H), 1.0 (s, 20H), 0.9­(t, 3H), 0.6 (t, 3H); C NMR (75 MHz, DMSO-*d*_6_, δ): 173, 165, 159, 144, 132, 117, 114, 110, 103, 68, 51, 35, 32, 31, 30, 29.02, 28.99, 28.92, 28.68, 28.61, 28.49, 28.06, 27, 25, 22; HRMS (*m*/*z* MH+): calcd for C_25_H_42_NO_4_, 420.3114; found, 420.3119.

Anal. Calcd for C_18_H_41_NO_4_: C, 71.56; H, 9.85. Found: C, 71.34; H, 9.85.

### Methyl 2-(Hexadecanyloxy)-4-propionamidosalicylate (**IIn**)

Yield (99%); mp 55–57 °C; FT-IR (ATR, cm^–1^): 3302, 2914, 2848, 1736, 1698, 1664, 1609, 1546, 1509, 1470, 1434, 1406, 1246, 1223, 1186, 1142, 1080, 841, 736, 719; H NMR (300 MHz, DMSO-*d*_6_, δ): 10.1 (s, 1H), 7.6 (d, 1H), 7.5 (s, 1H), 7.1 (dd, 1H), 3.9 (t, 2H), 3.7 (s, 3H), 2.3 (q, 2H), 1.7 (m, 2H), 1.5 (m, 2H), 1.2 (s, 24H), 1.1 (t, 3H), 0.8 (t, 3H); C NMR (75 MHz, DMSO-*d*_6_, δ): 173, 165, 159, 144, 132, 114, 110, 103, 68, 51, 31, 29.61, 29.01, 28.92, 28.62, 28.50, 28.97, 25, 22 14, 9 (coincident chemical shifts very likely); HRMS (*m*/*z* MH+): calcd for C_27_H_46_NO_4_, 448.3427; found, 448.3427.

Anal. Calcd for C_27_H_45_NO_4_: C, 72.44; H, 10.13. Found: C, 72.52; H, 10.45.

### Methyl 2-(Octadecanyloxy)-4-propionamidosalicylate (**IIo**)

Yield (85%); mp 65–67 °C; FT-IR (ATR, cm^–1^): 3302, 2914, 2849, 1736, 1697, 1665, 1612, 1547, 1471, 1406, 1249, 1223, 1186, 1142, 1080, 842, 737, 718; H NMR (300 MHz, DMSO-*d*_6_, δ): 10.1 (s, 1H), 7.7 (d, 1H), 7.5 (s, 1H), 7.2 (d, 1H), 4.0 (t, 2H), 3.7 (s, 3H), 2.3 (q, 2H), 1.7 (m, 2H), 1.4 (m, 2H), 1.3 (s, 28H), 1.1 (t, 3H), 0.8 (t, 3H); C NMR (75 MHz, DMSO-*d*_6_, δ): 173, 165, 159, 144, 132, 114, 110, 103, 68, 51, 31, 29.61, 28.98, 28.90, 28.67, 28.60, 26, 25, 22, 14, 9 (coincident chemical shifts very likely); HRMS (*m*/*z* MH+): calcd for C_29_H_50_NO_4_, 476.3740; found, 476.3747.

Anal. Calcd for C_29_H_49_NO_4_: C, 73.22; H, 10.38. Found: C, 73.08; H, 10.59.

## Supplementary Material





## Notes

FT-IR, HNMR, and CNMR spectra for all compounds (PDF)
Table S1. ^13^C NMR chemical shifts of compounds **IIa**–**IIo**. Tabulation of significant functional group similarities (PDF)
