# Supplementary material for: Preparation of Lipophilic Derivatives of para-Aminosalicylic Acid for Antimicrobial Drug Design
Source: ACS Omega. 2025 Sep 24;10(39):46134–40. doi: 10.1021/acsomega.5c08092 (PMC12508941; doi:10.1021/acsomega.5c08092)
Supplement: Supplementary file 1 [file ao5c08092_si_001.pdf]

Preparation of Lipophilic Derivatives of *para*-Aminosalicylic Acid for Antimicrobial Drug Design

Michael J. Hearn* and Alice K. Min

Department of Chemistry
Wellesley College
Wellesley, Massachusetts 02481 USA

*Corresponding Author; E-Mail: MHearn@Wellesley.edu

Supplementary Materials

FT-IR, ^{1}H NMR and ^{13}C NMR Spectra of All the Novel Compounds

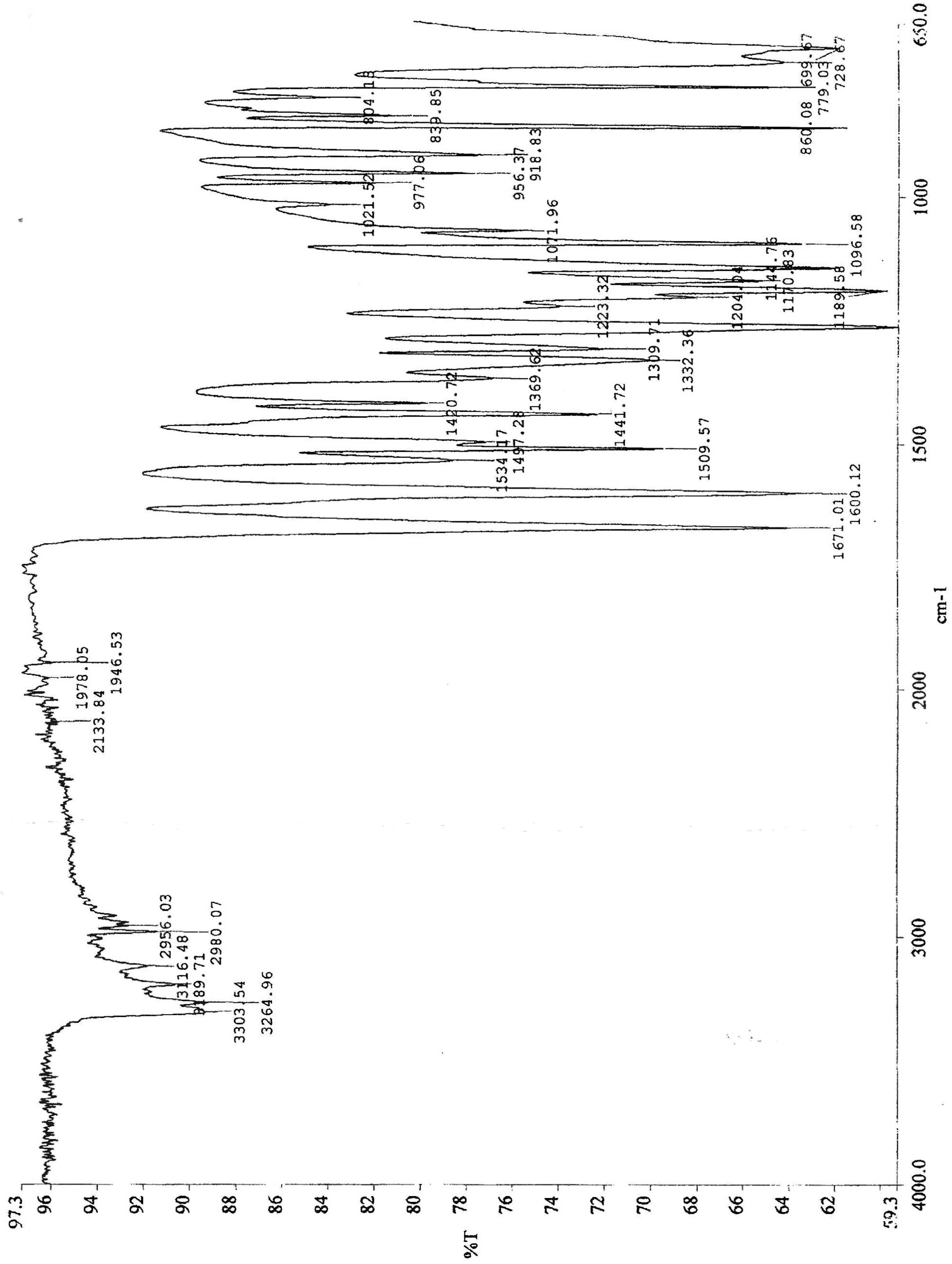

Figure I.i FT-IR Spectrum of Compound I
Methyl 4-propionamido salicylate (Propionamido Compound, PAC), $C_{11}H_{13}NO_4$

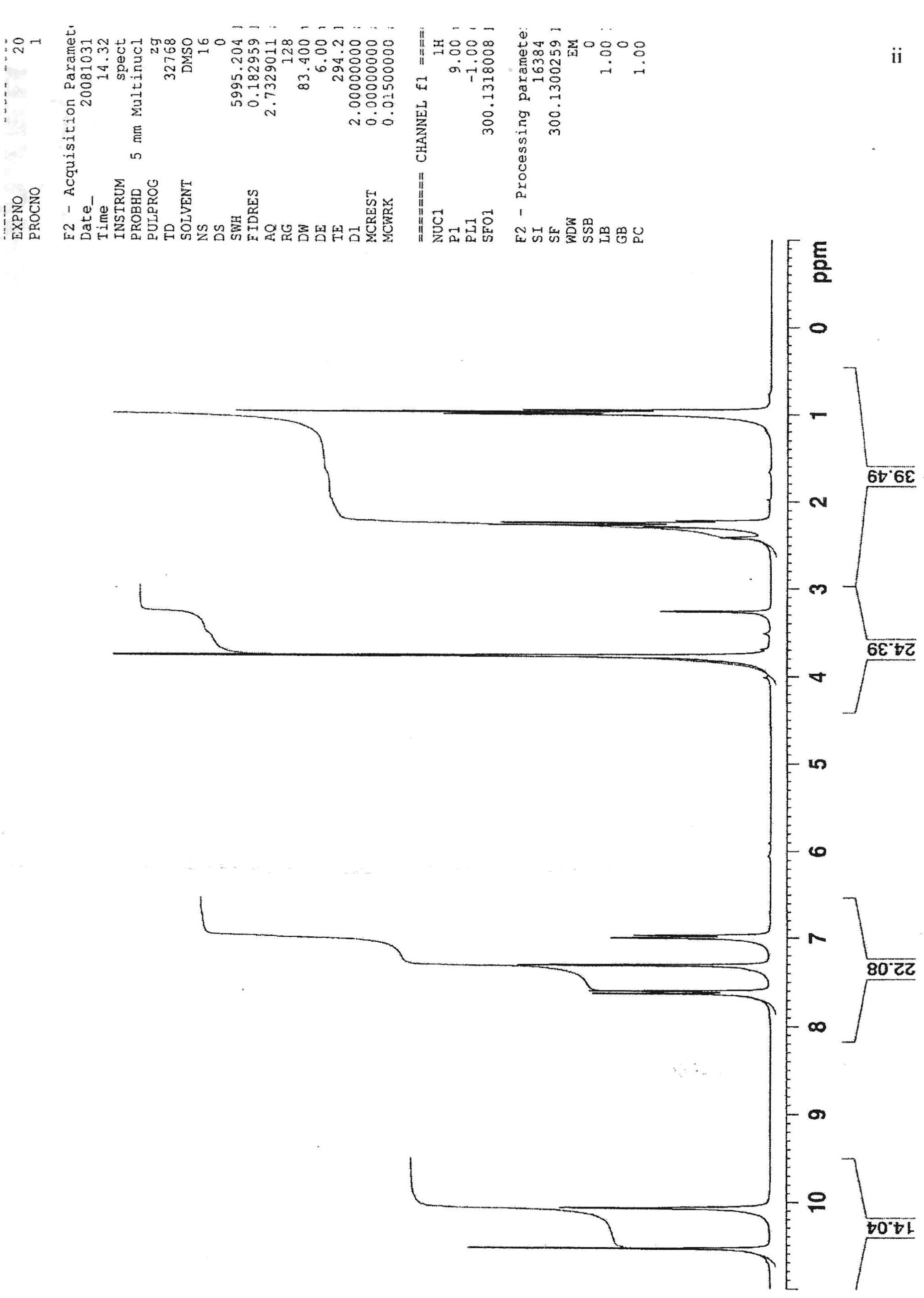

Figure I.ii ^{1}H NMR Spectrum of Compound I
Methyl 4-propionamido salicylate (Propionamido Compound, PAC), $C_{11}H_{13}NO_4$

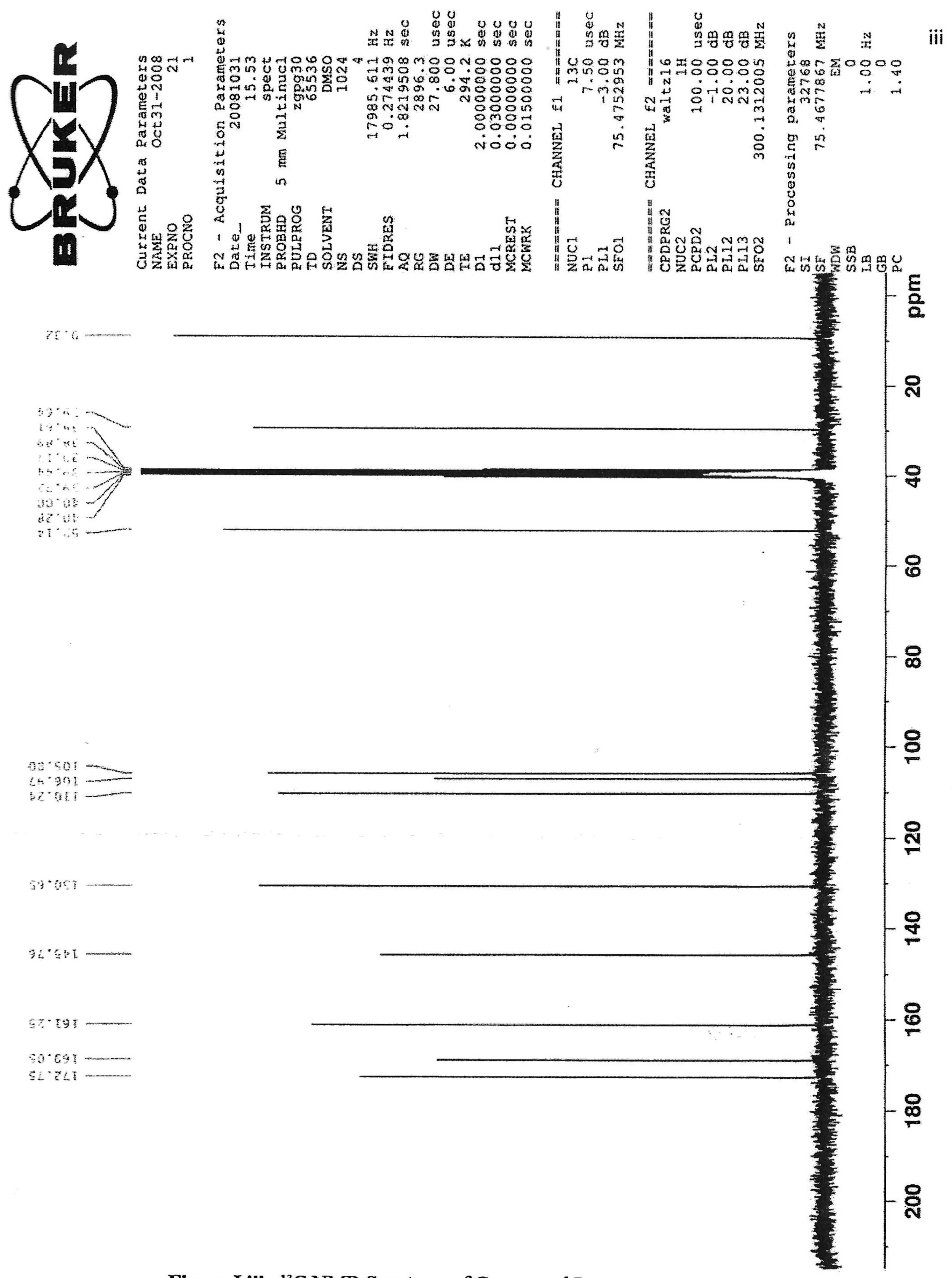

Figure I.iii ^{13}C NMR Spectrum of Compound I
Methyl 4-propionamido salicylate (Propionamido Compound, PAC), $C_{11}H_{13}NO_4$

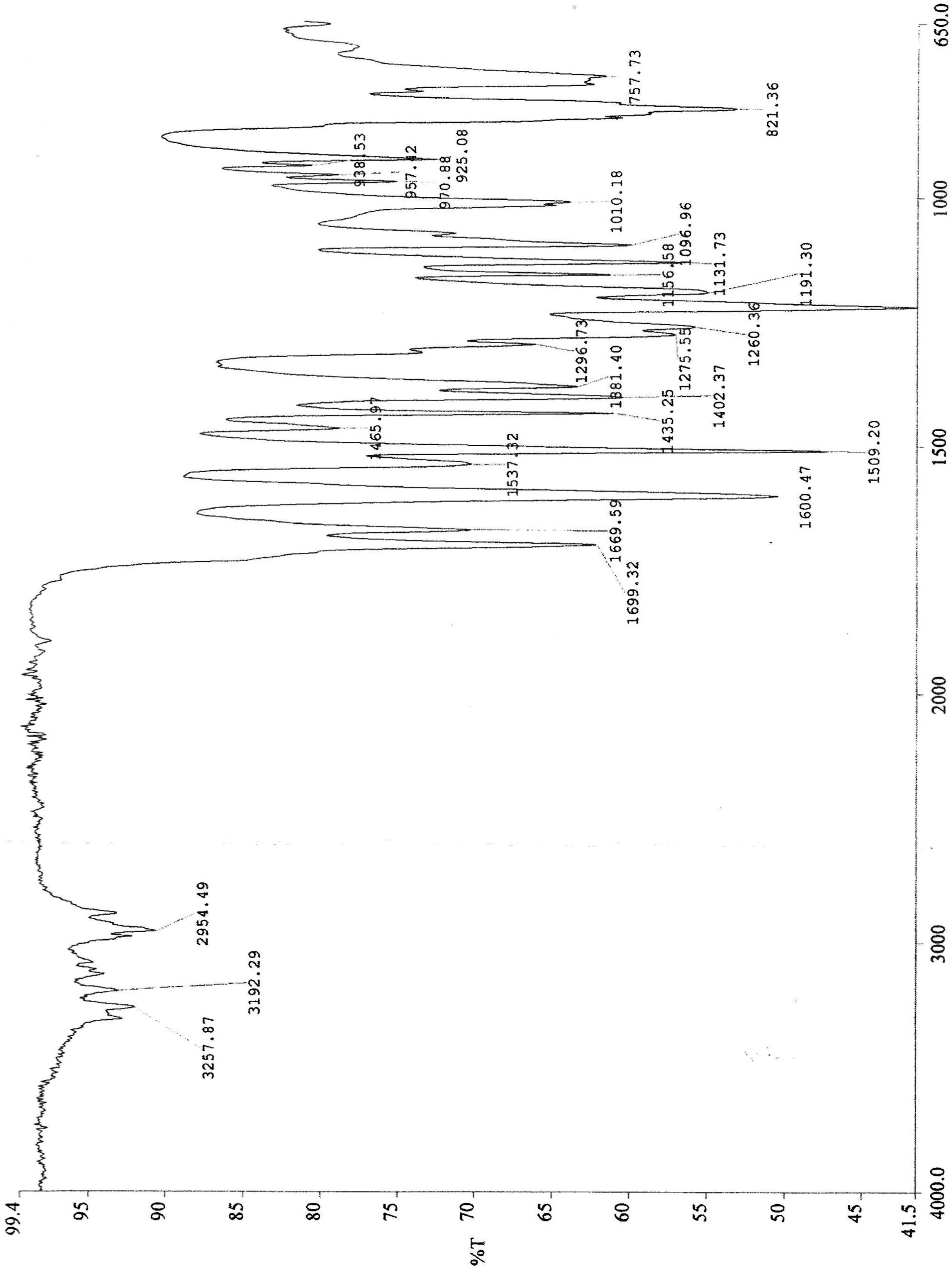

Figure IIa.i ^{1}H NMR Spectrum of Compound IIa
Methyl 2-(4-fluorobenzyloxy)-4-propionamido salicylate, $C_{18}H_{18}NO_4F$

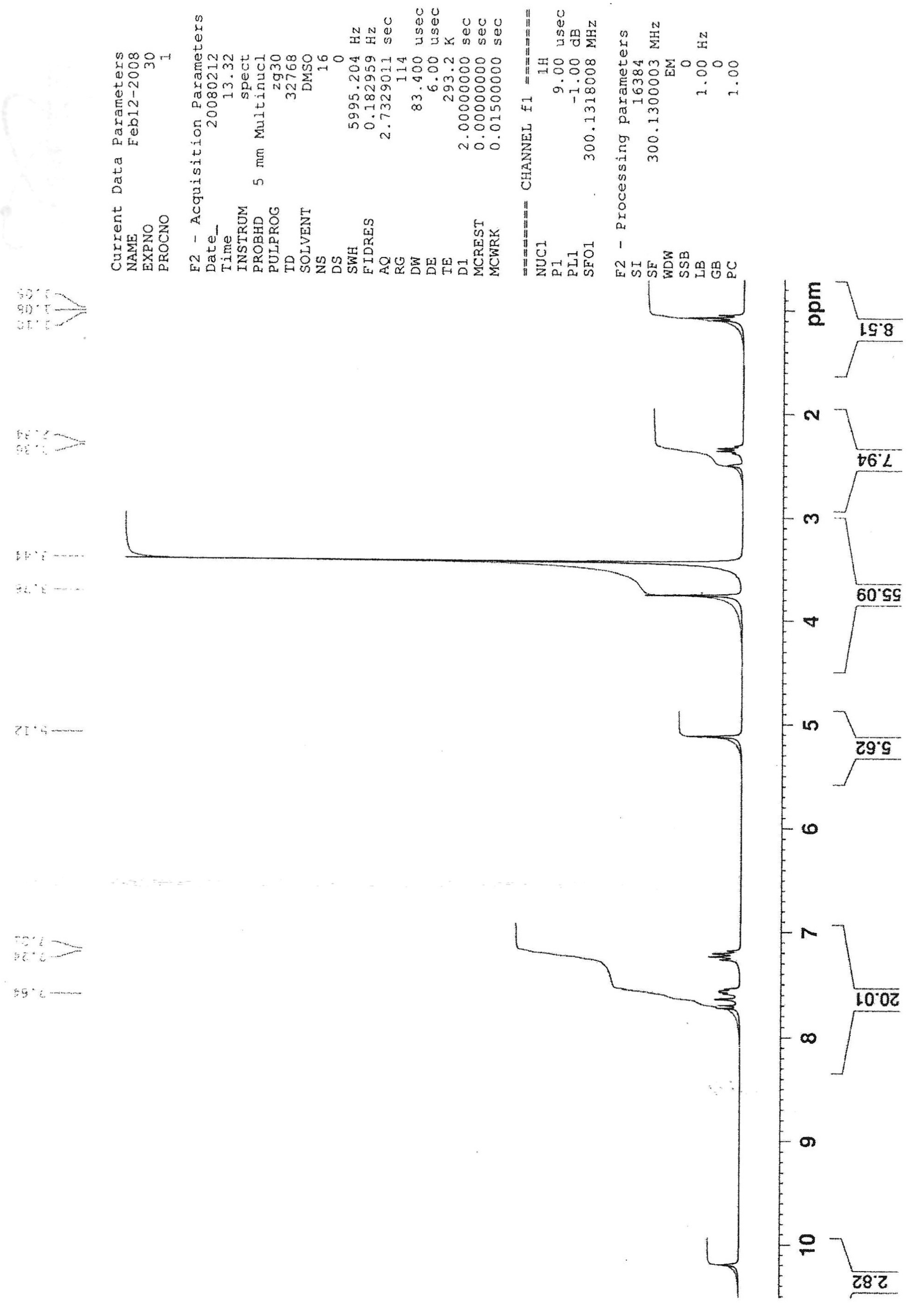

Figure IIa.ii ^{1}H NMR Spectrum of Compound IIa
Methyl 2-(4-fluorobenzyloxy)-4-propionamido salicylate, $C_{18}H_{18}NO_4F$

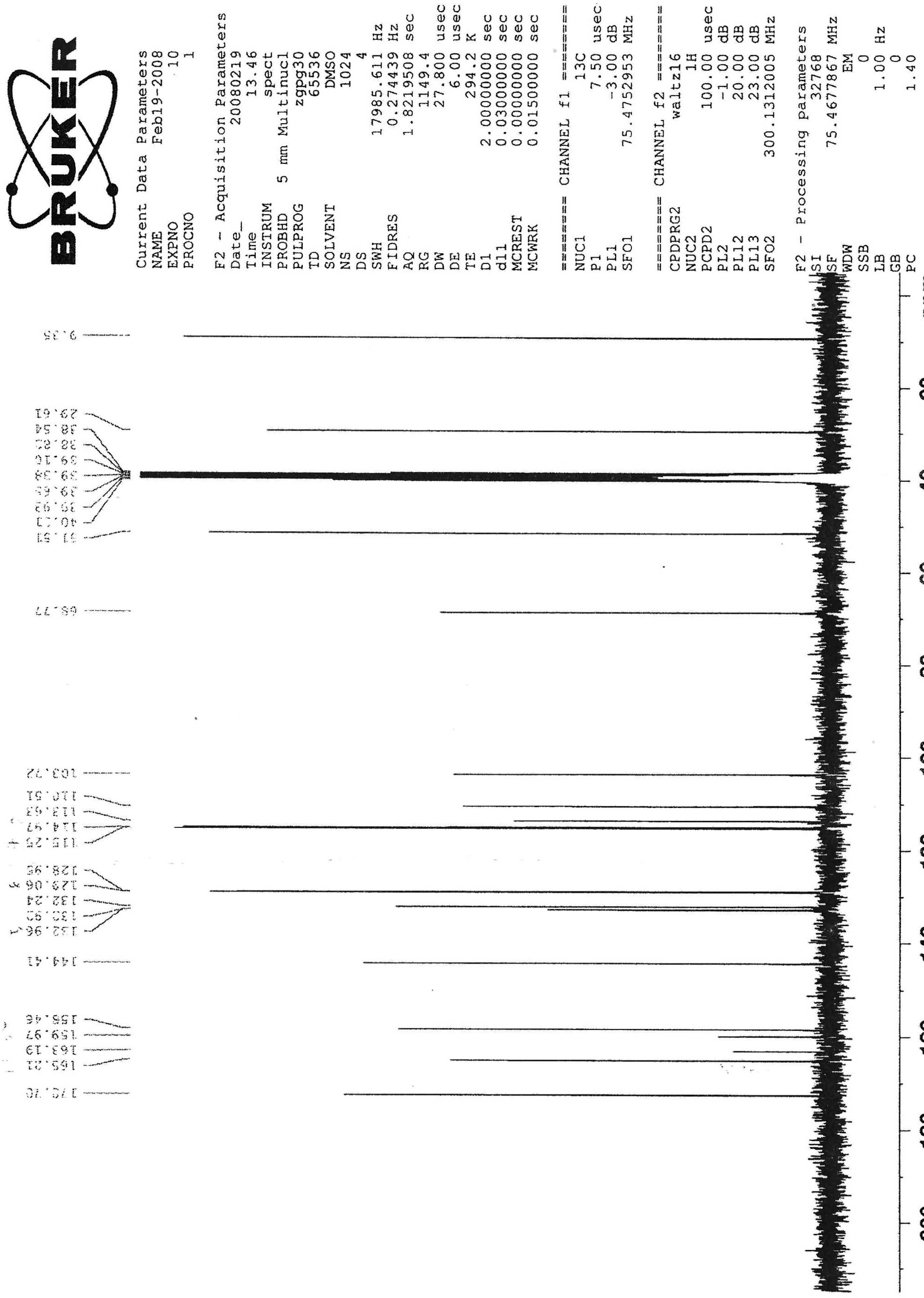

Figure IIa.iii ^{13}C NMR Spectrum of Compound IIa
Methyl 2-(4-fluorobenzyloxy)-4-propionamido salicylate, $C_{18}H_{18}NO_4F$

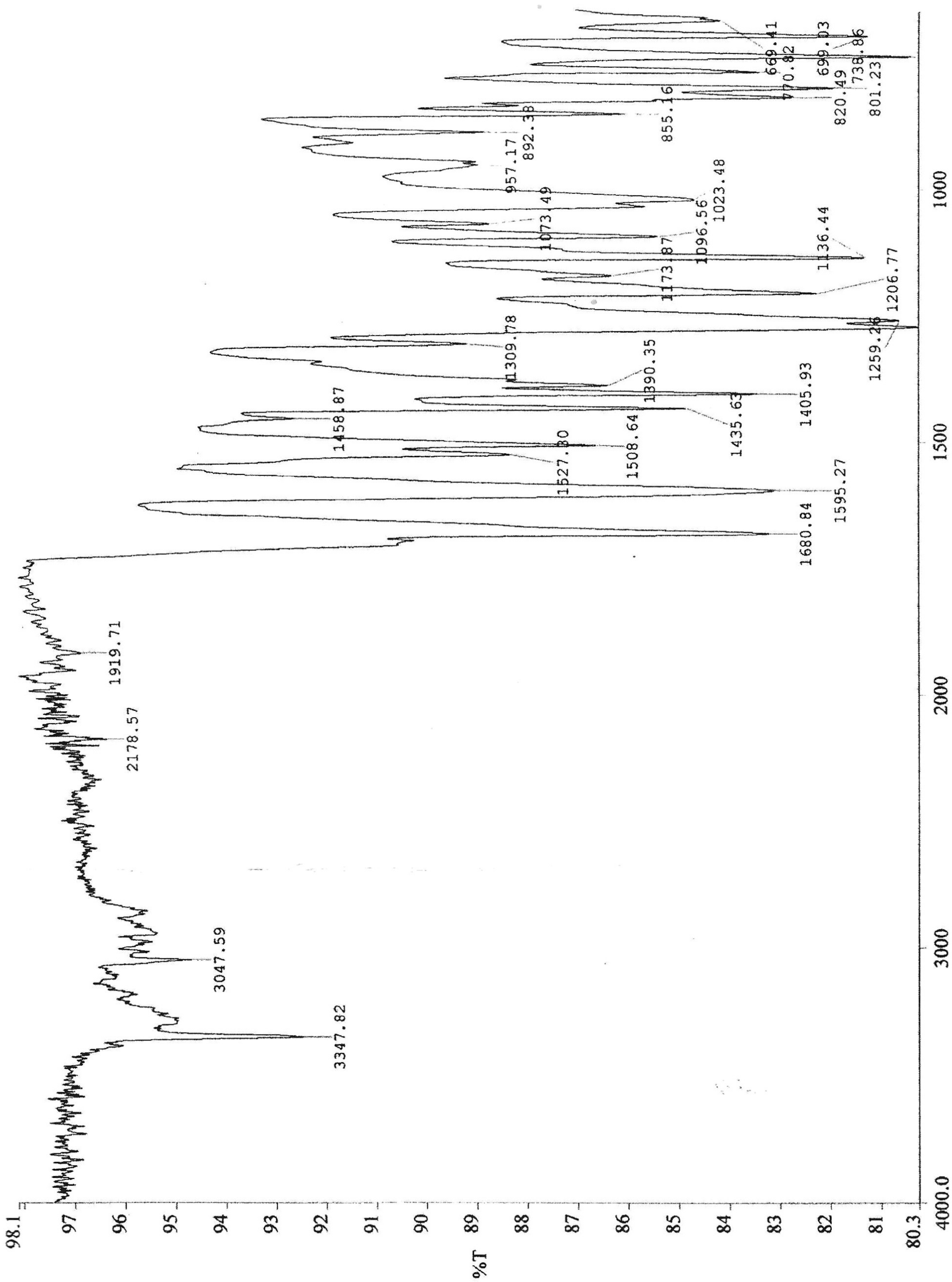

Figure IIb.i FT-IR Spectrum of Compound IIb
Methyl 2-((2-naphthyl)methoxy)-4-propionamido salicylate, $C_{22}H_{21}NO_4$

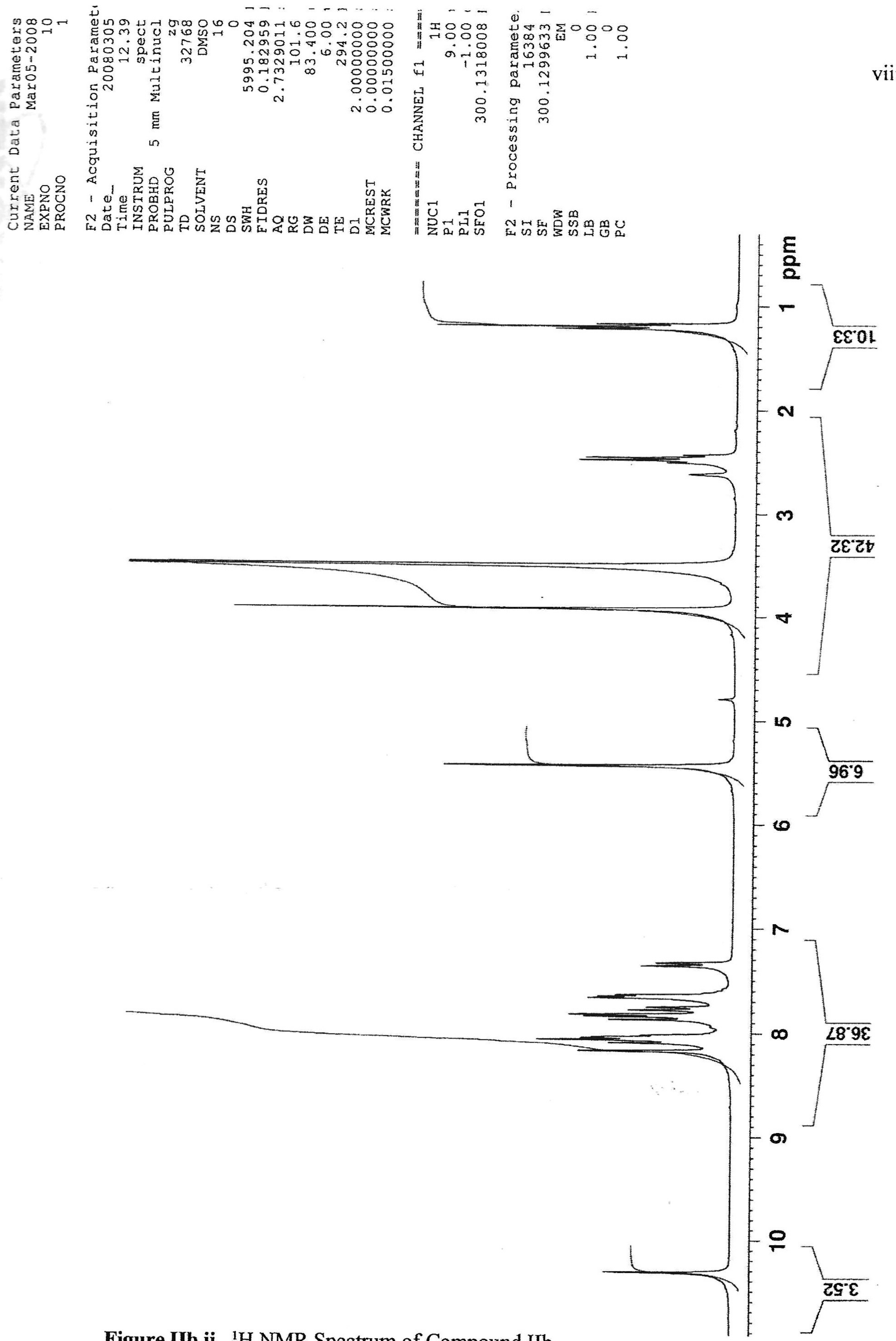

Figure IIb.ii ^{1}H NMR Spectrum of Compound IIb
Methyl 2-((2-naphthyl)methoxy)-4-propionamido salicylate, $C_{22}H_{21}NO_4$

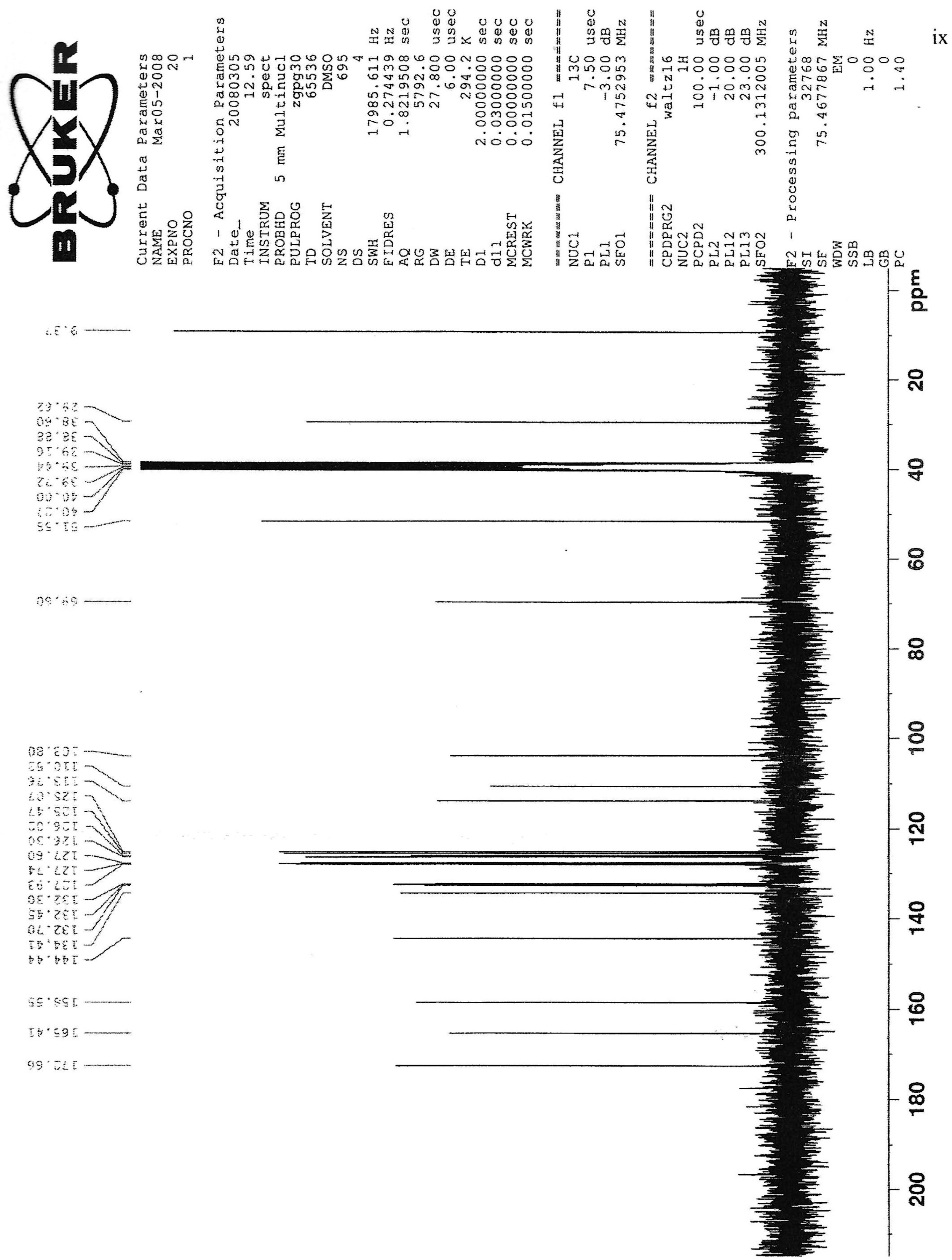

Figure IIb.iii ^{13}C NMR Spectrum of Compound IIb
Methyl 2-((2-naphthyl)methoxy)-4-propionamido salicylate, $C_{22}H_{21}NO_4$

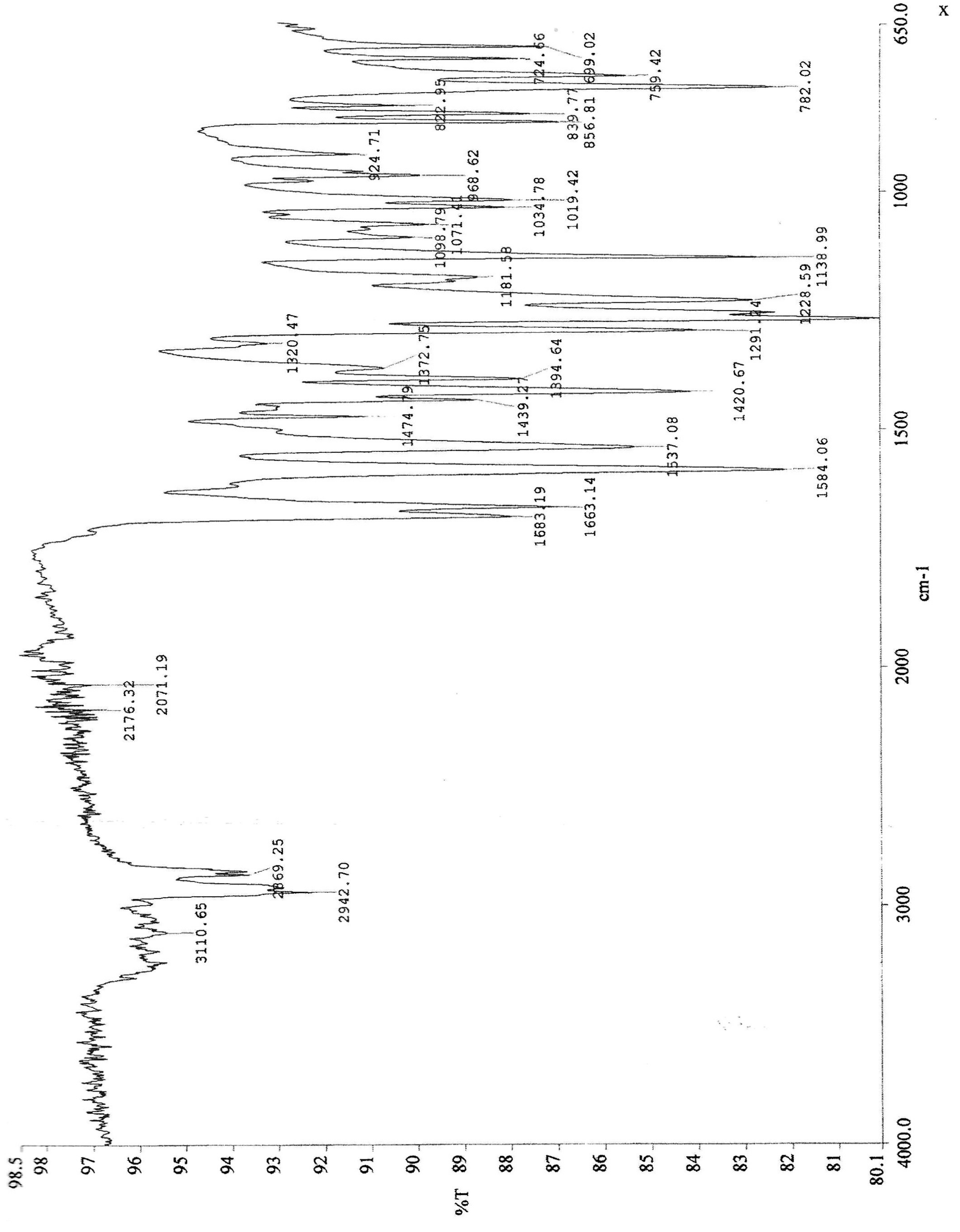

Figure IIc.i FT-IR Spectrum of Compound IIc
Methyl 2-(heptyloxy)-4-propionamido salicylate, $C_{18}H_{27}NO_4$

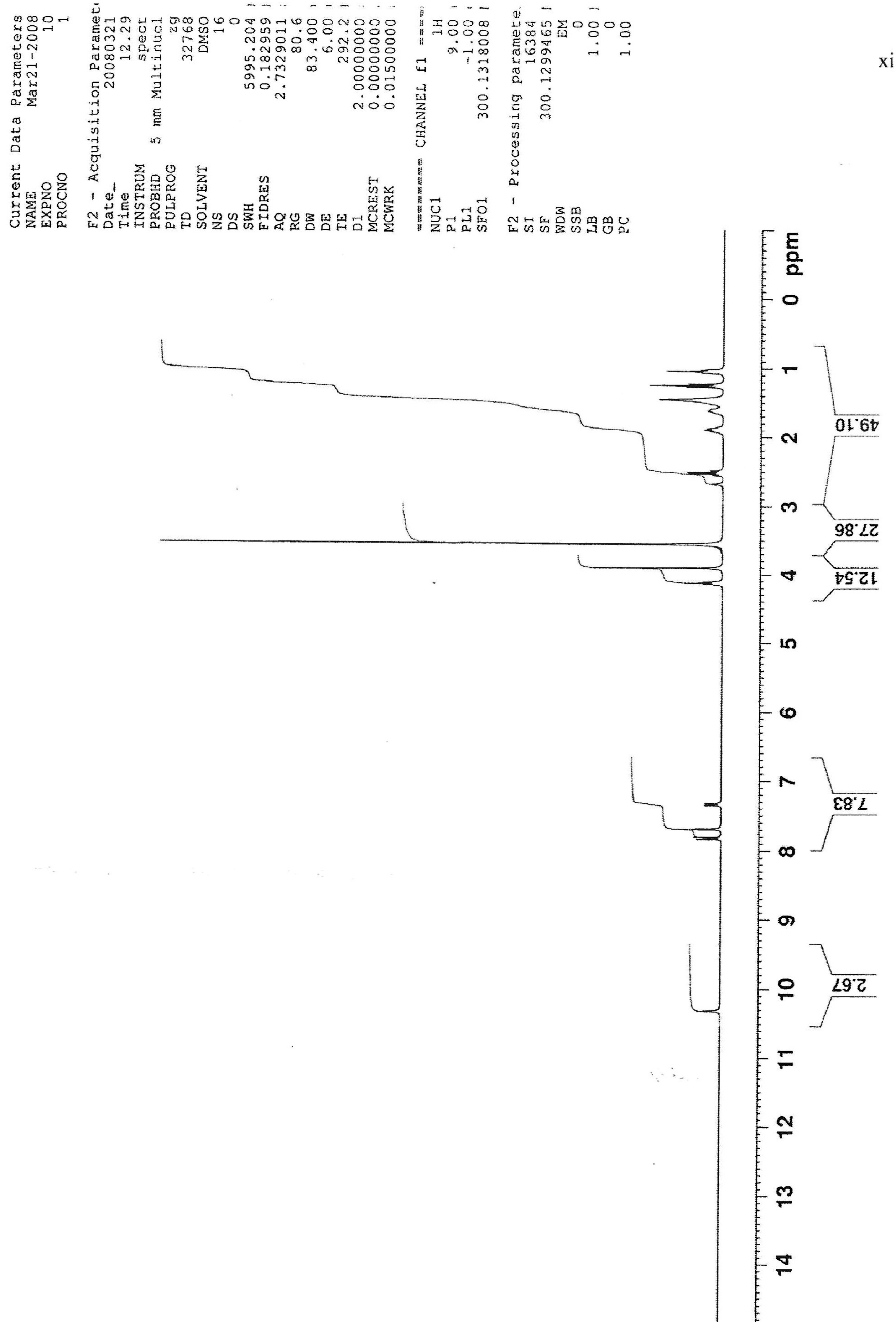

Figure IIc.ii ^{1}H NMR Spectrum of Compound IIc
Methyl 2-(heptyloxy)-4-propionamido salicylate, $C_{18}H_{27}NO_4$

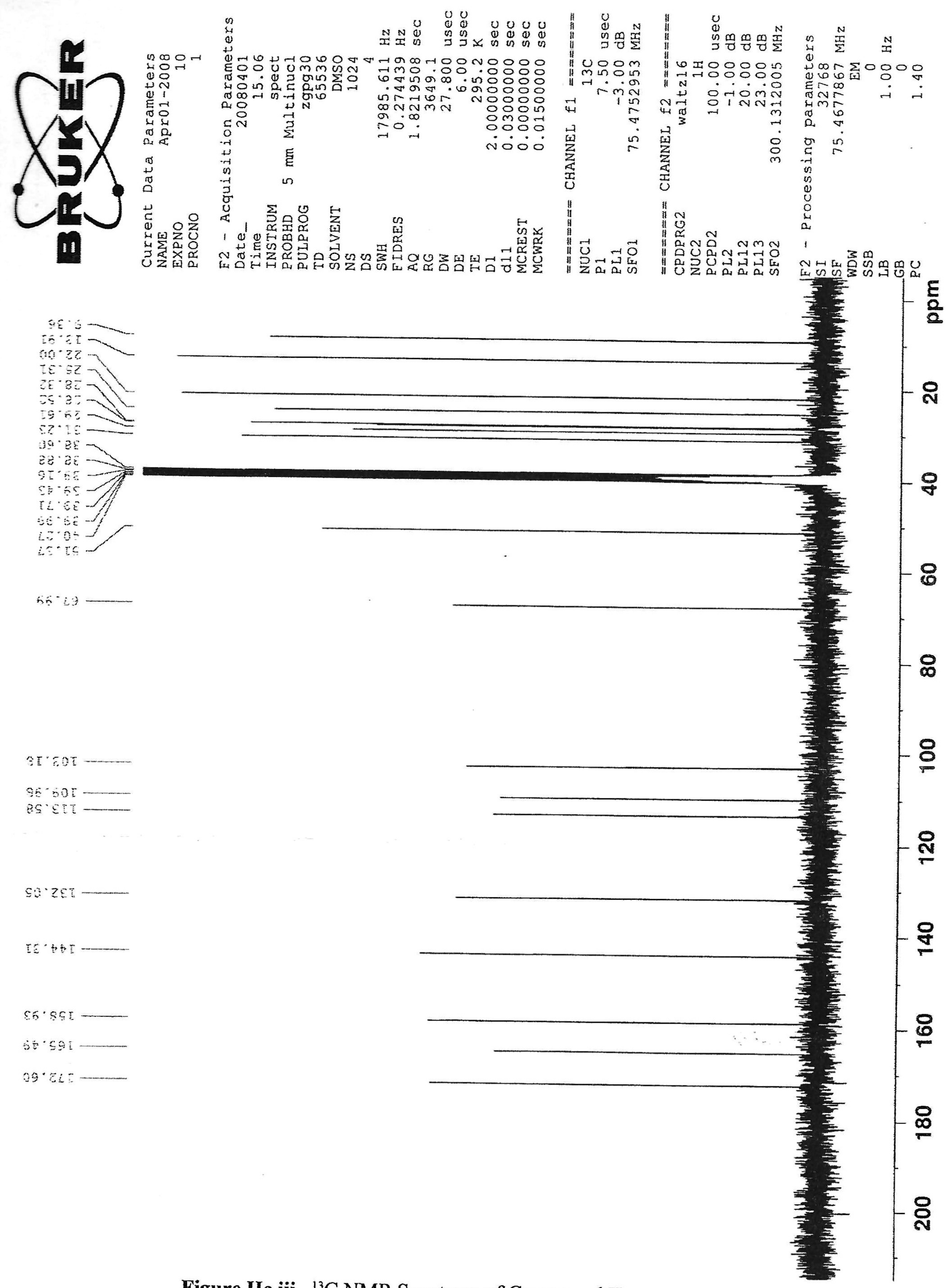

Figure IIc.iii ^{13}C NMR Spectrum of Compound IIc
Methyl 2-(heptyloxy)-4-propionamido salicylate, $C_{18}H_{27}NO_4$

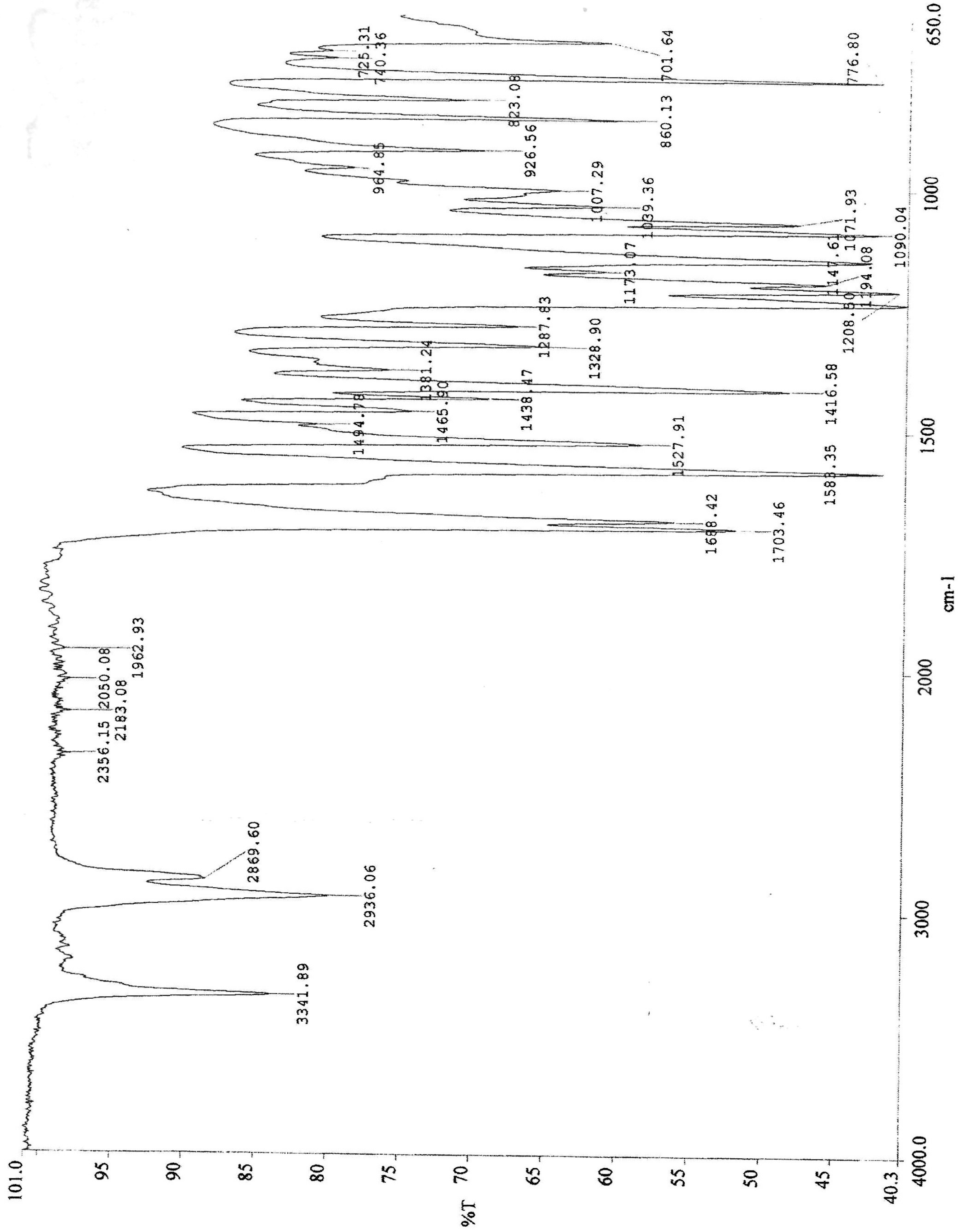

Figure IId.i FT-IR Spectrum of Compound IId
Methyl 2-(hexyloxy)-4-propionamido salicylate, $C_{17}H_{25}NO_4$

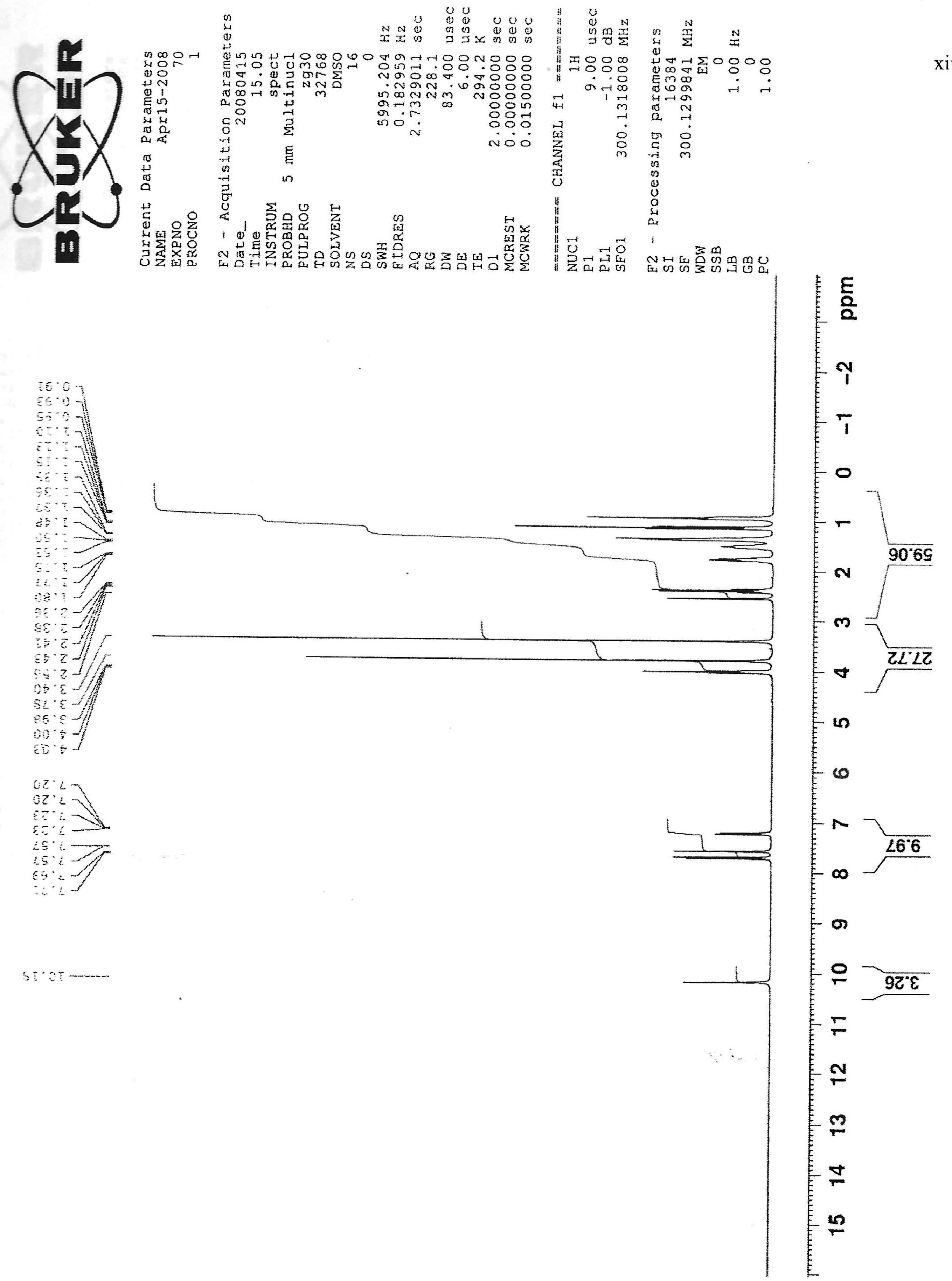

Figure IId.ii ^{1}H NMR Spectrum of Compound IId
Methyl 2-(hexyloxy)-4-propionamido salicylate, $C_{17}H_{25}NO_4$

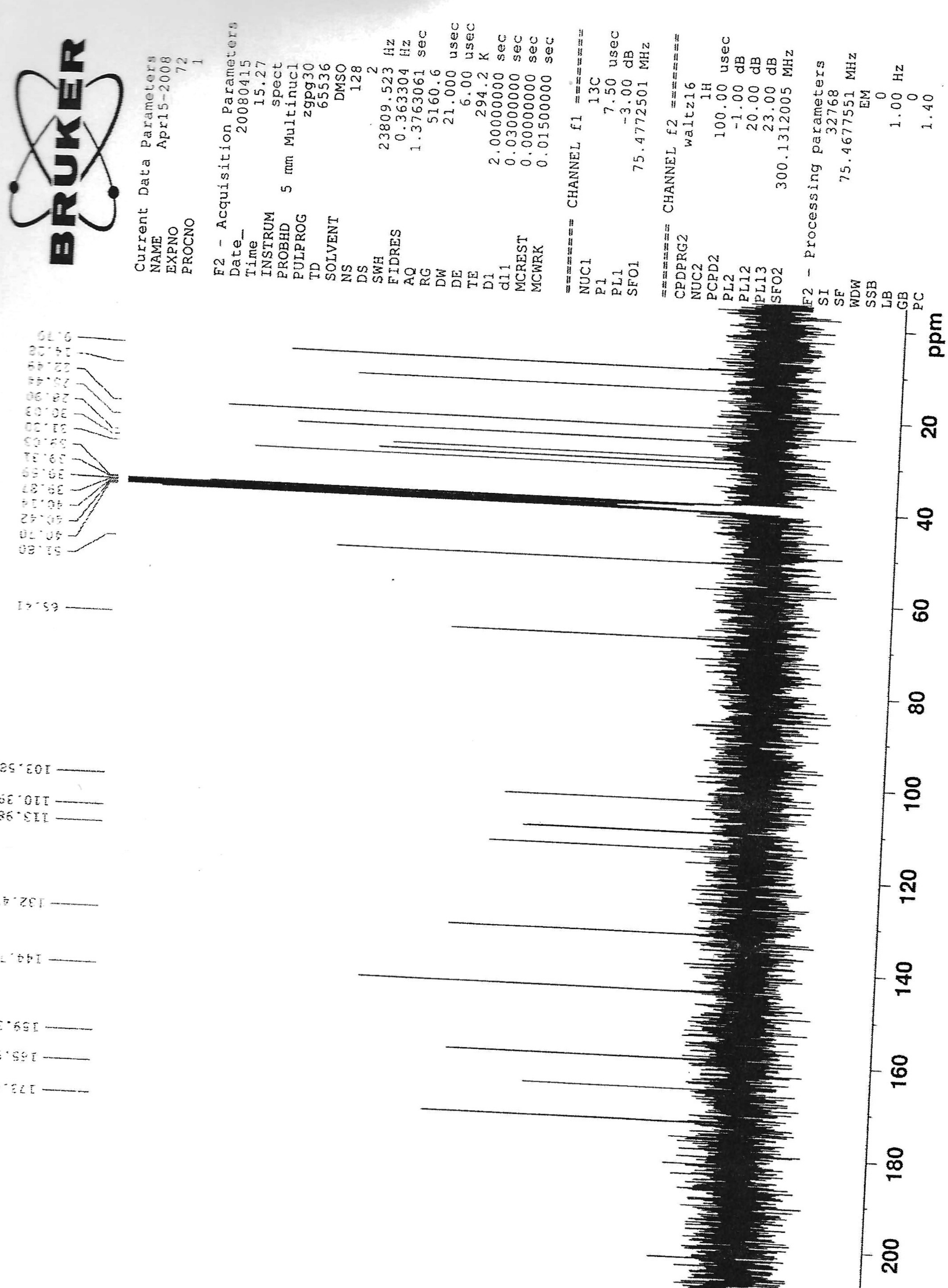

Figure IId.iii ^{13}C NMR Spectrum of Compound IId
Methyl 2-(hexyloxy)-4-propionamido salicylate, $C_{17}H_{25}NO_4$

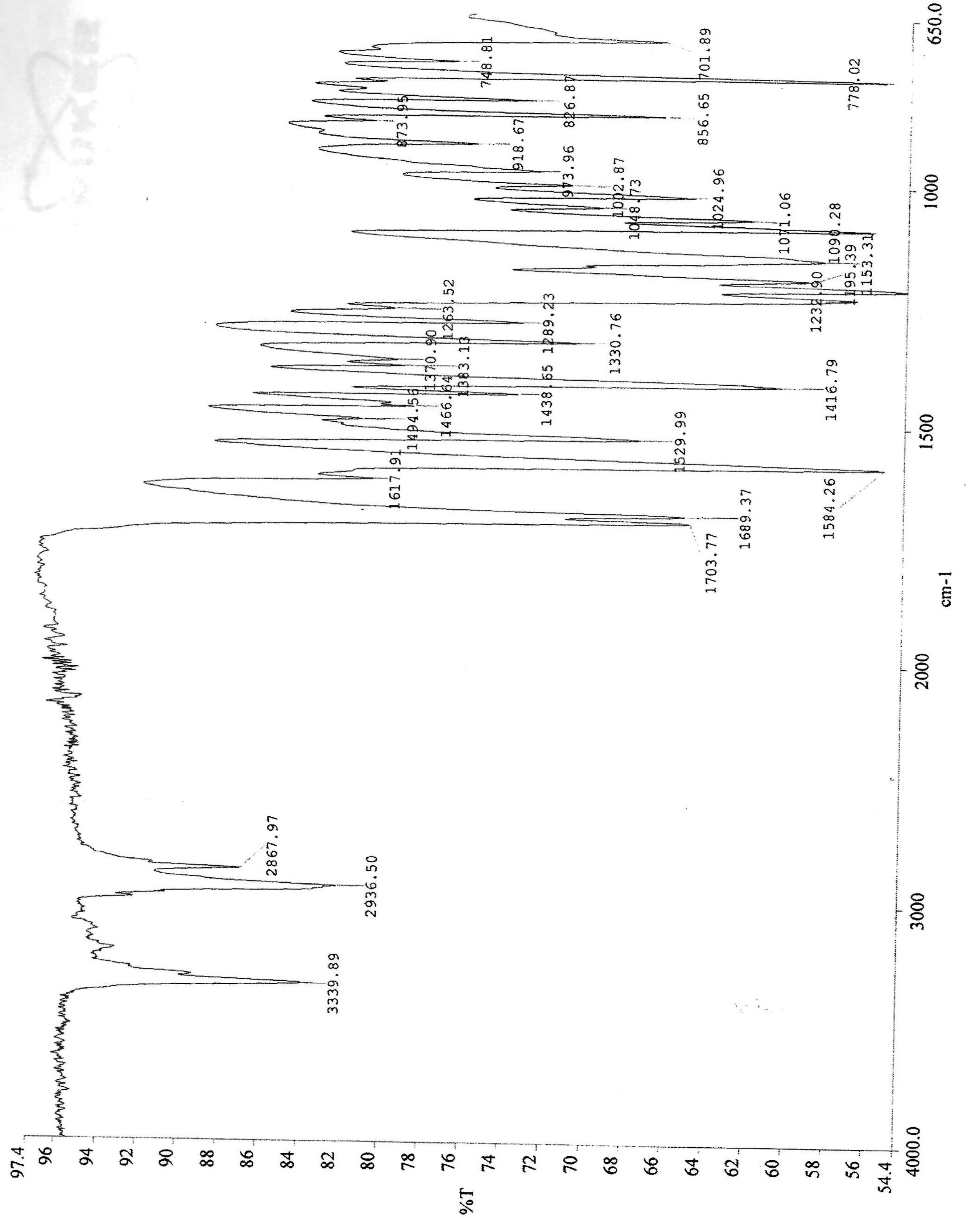

Figure IIe.i FT-IR Spectrum of Compound IIe
Methyl 2-(pentyloxy)-4-propionamido salicylate, $C_{16}H_{23}NO_4$

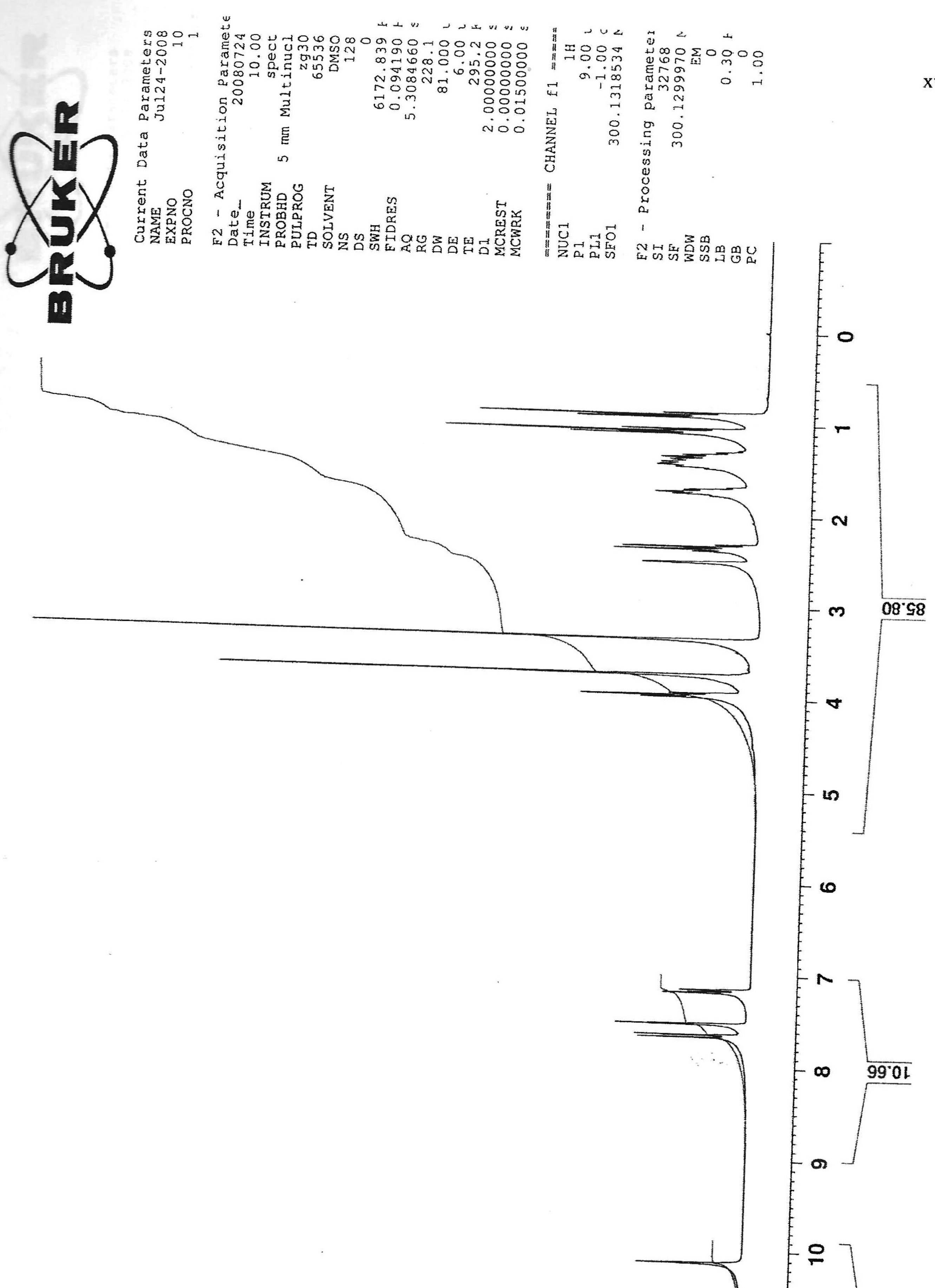

Figure IIe.ii ^{1}H NMR Spectrum of Compound IIe
Methyl 2-(pentyloxy)-4-propionamido salicylate, $C_{16}H_{23}NO_4$

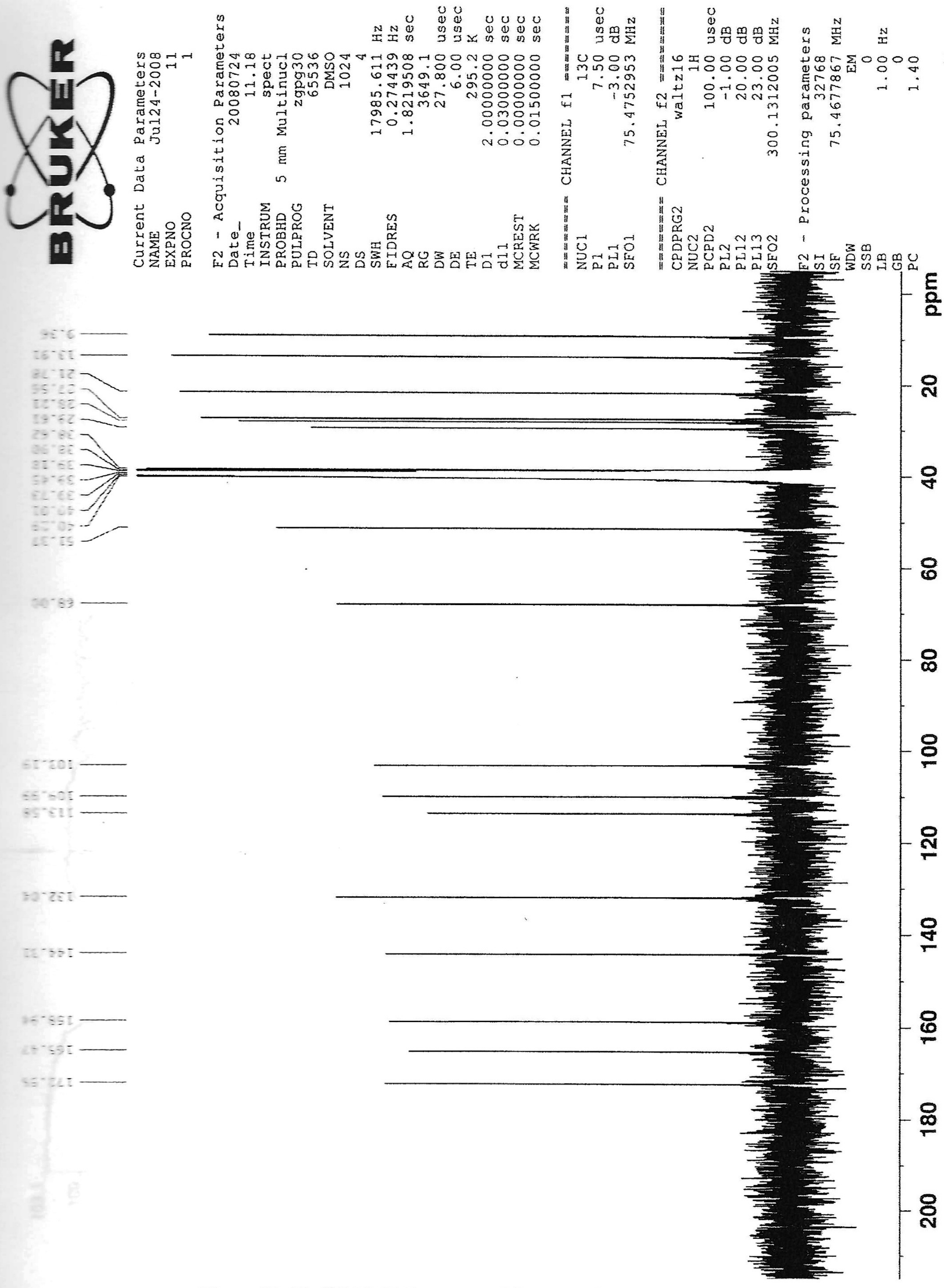

Figure IIe.iii ^{13}C NMR Spectrum of Compound IIe
Methyl 2-(pentyloxy)-4-propionamido salicylate, $C_{16}H_{23}NO_4$

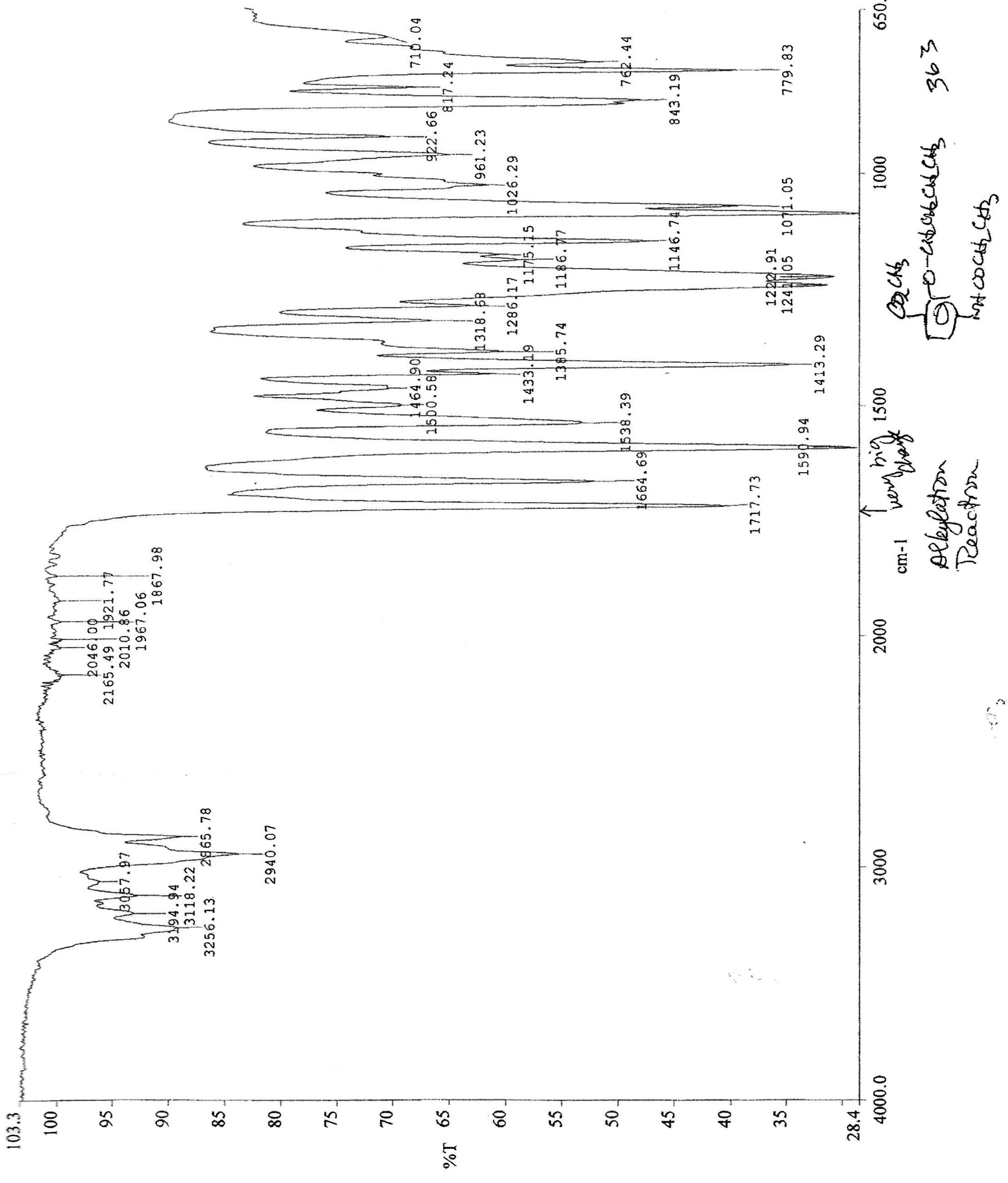

Figure IIf.i FT-IR Spectrum of Compound IIf
Methyl 2-(butyloxy)-4-propionamido salicylate, $C_{15}H_{21}NO_4$

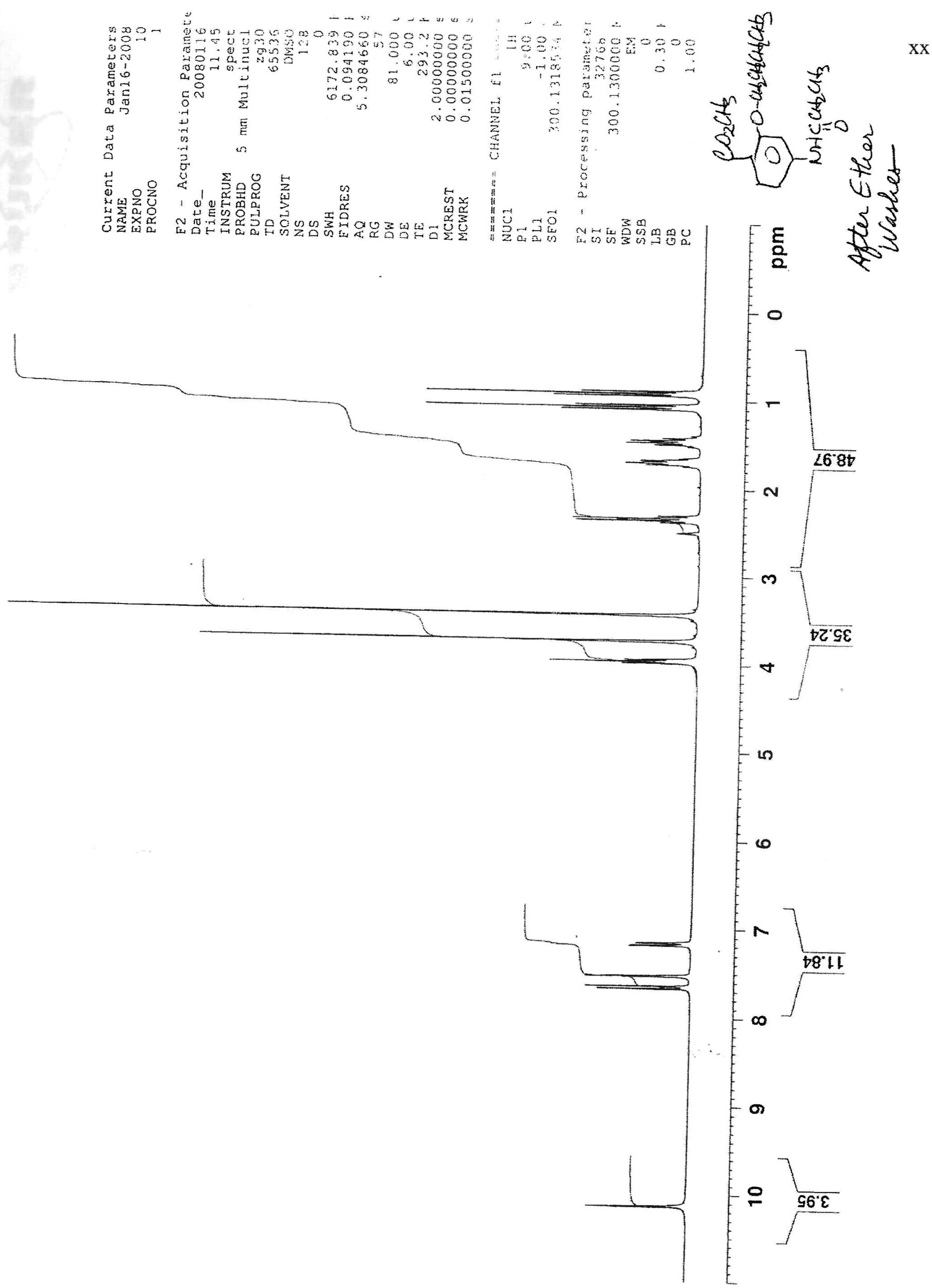

Figure IIf.ii ^{1}H NMR Spectrum of Compound IIf
Methyl 2-(butyloxy)-4-propionamido salicylate, $C_{15}H_{21}NO_4$

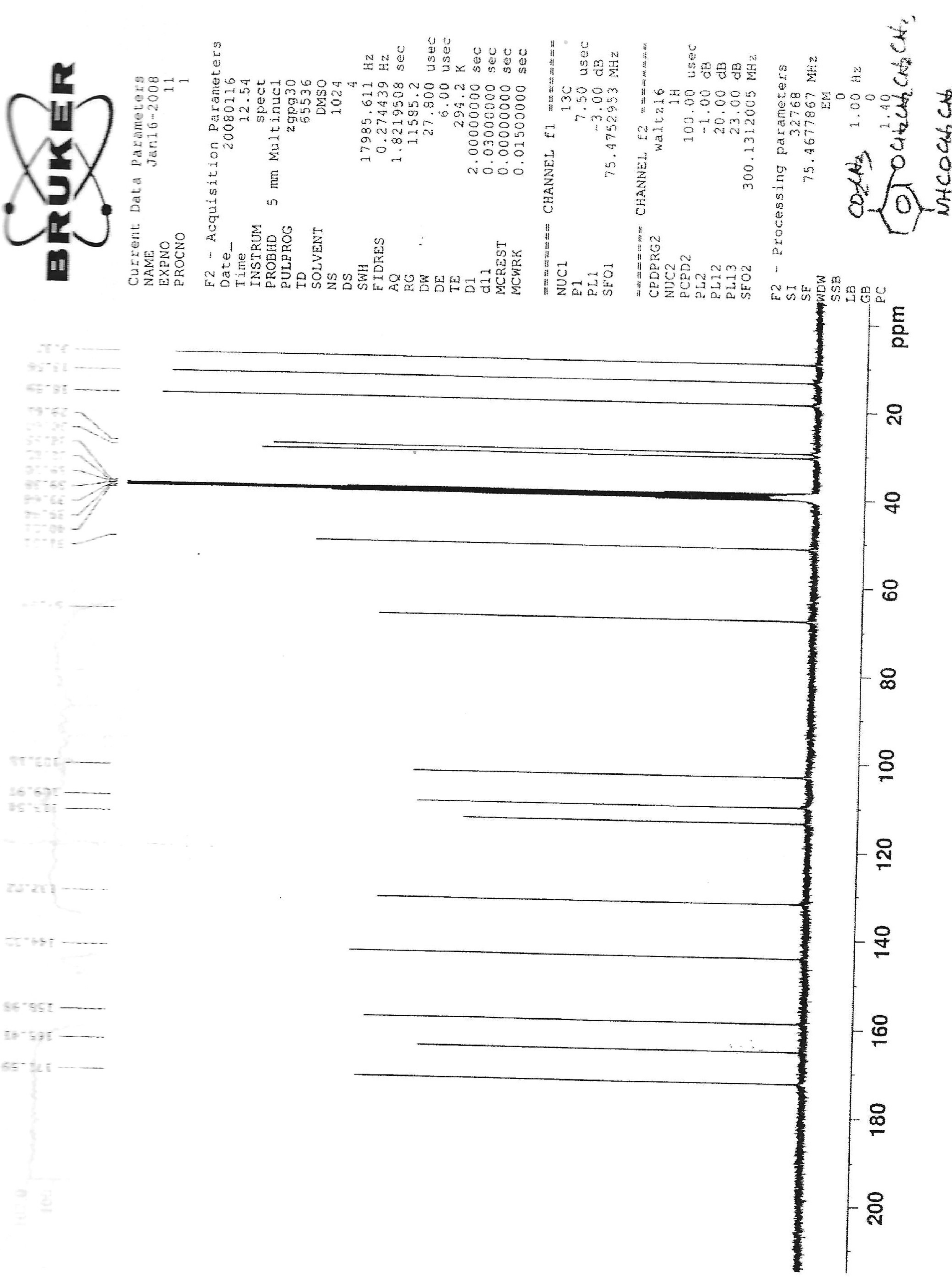

Figure IIf.iii ^{13}C NMR Spectrum of Compound IIf
Methyl 2-(butyloxy)-4-propionamido salicylate, $C_{15}H_{21}NO_4$

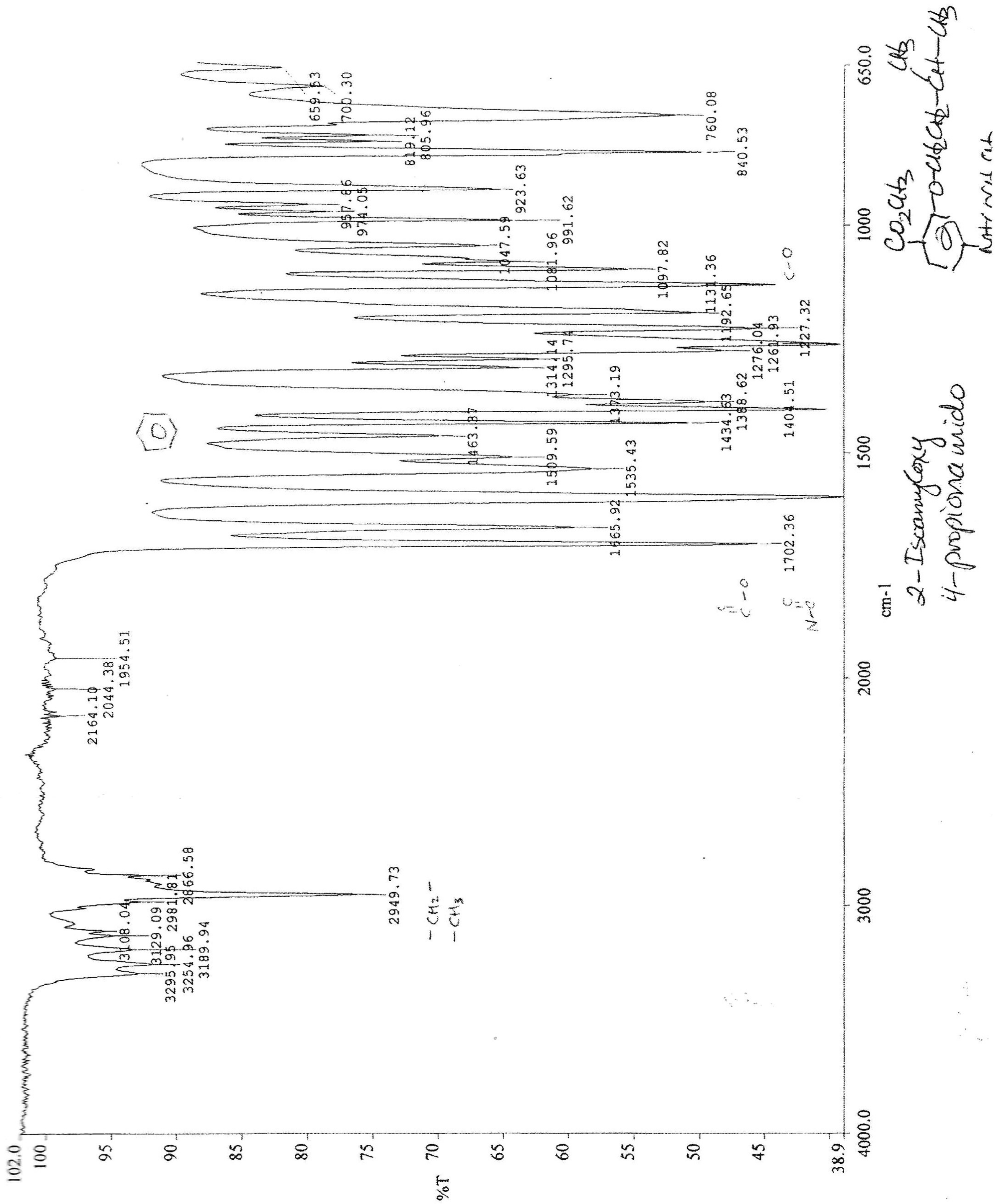

Figure IIg.i FT-IR Spectrum of Compound IIg
Methyl 2-((3-methyl)butyloxy)-4-propionamido salicylate, $C_{16}H_{23}NO_4$

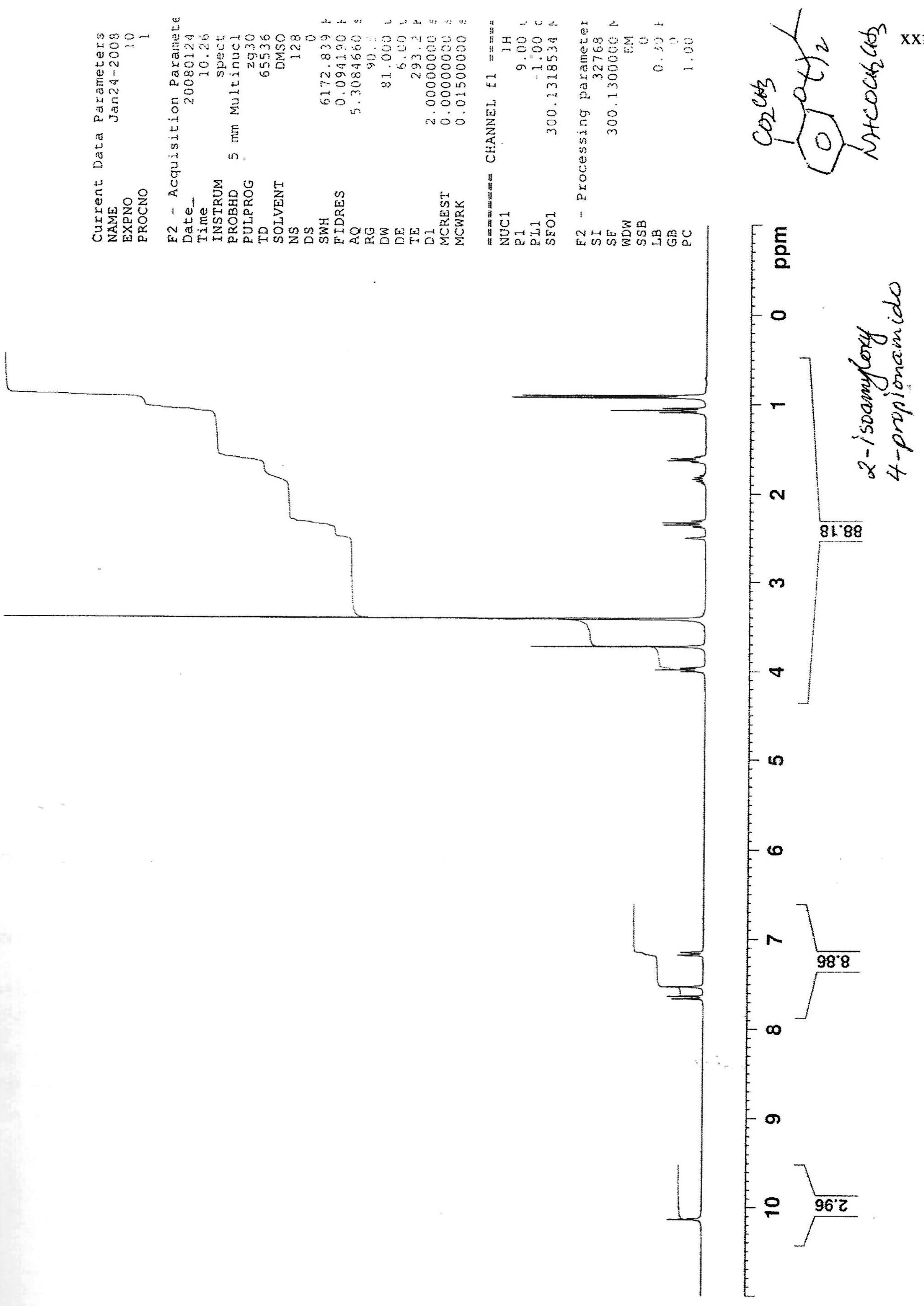

Figure IIg.ii ^{1}H NMR Spectrum of Compound IIg
Methyl 2-((3-methyl)butyloxy)-4-propionamido salicylate, $C_{16}H_{23}NO_4$

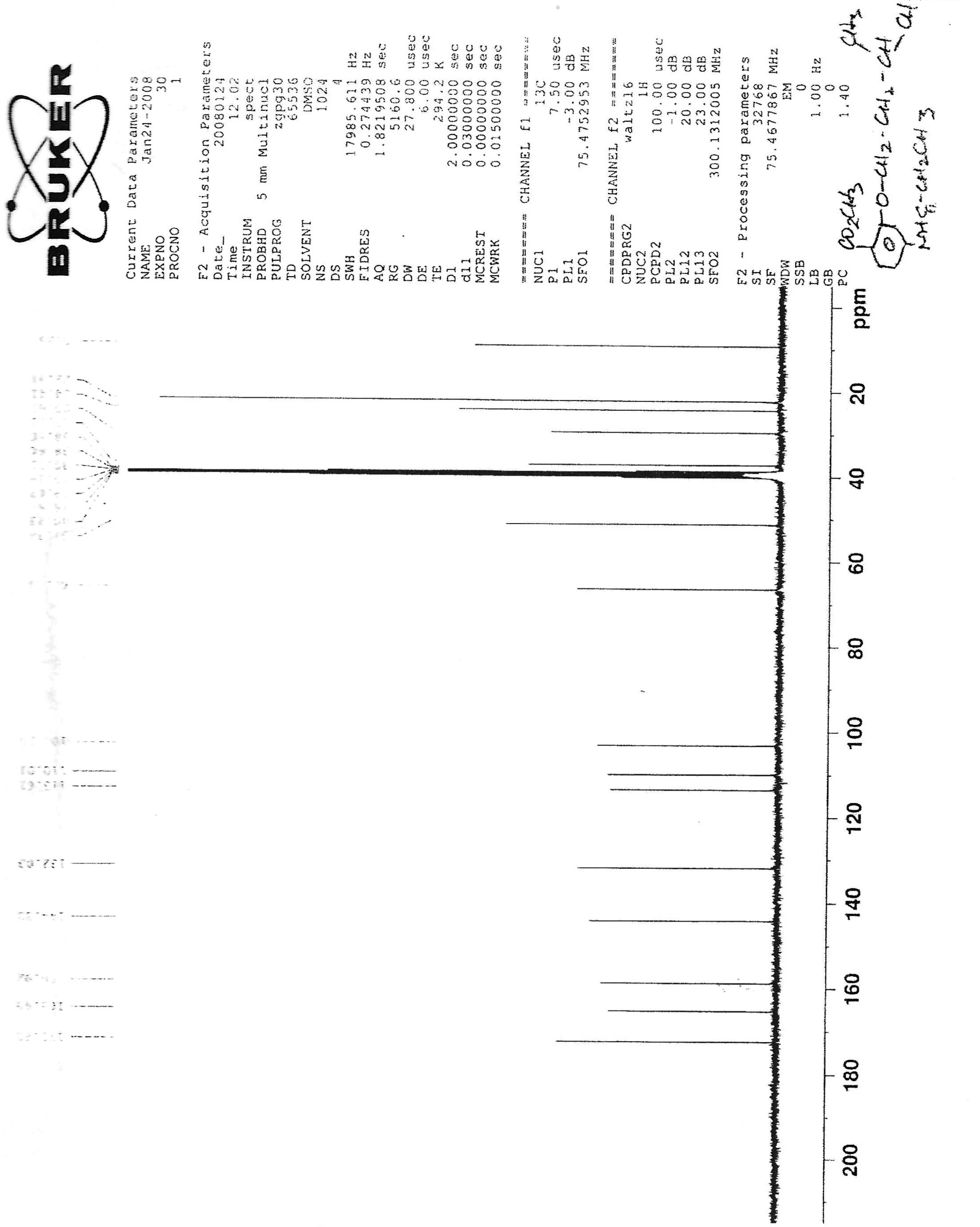

Figure IIg.iii ^{13}C NMR Spectrum of Compound IIg
Methyl 2-((3-methyl)butyloxy)-4-propionamido salicylate, $C_{16}H_{23}NO_4$

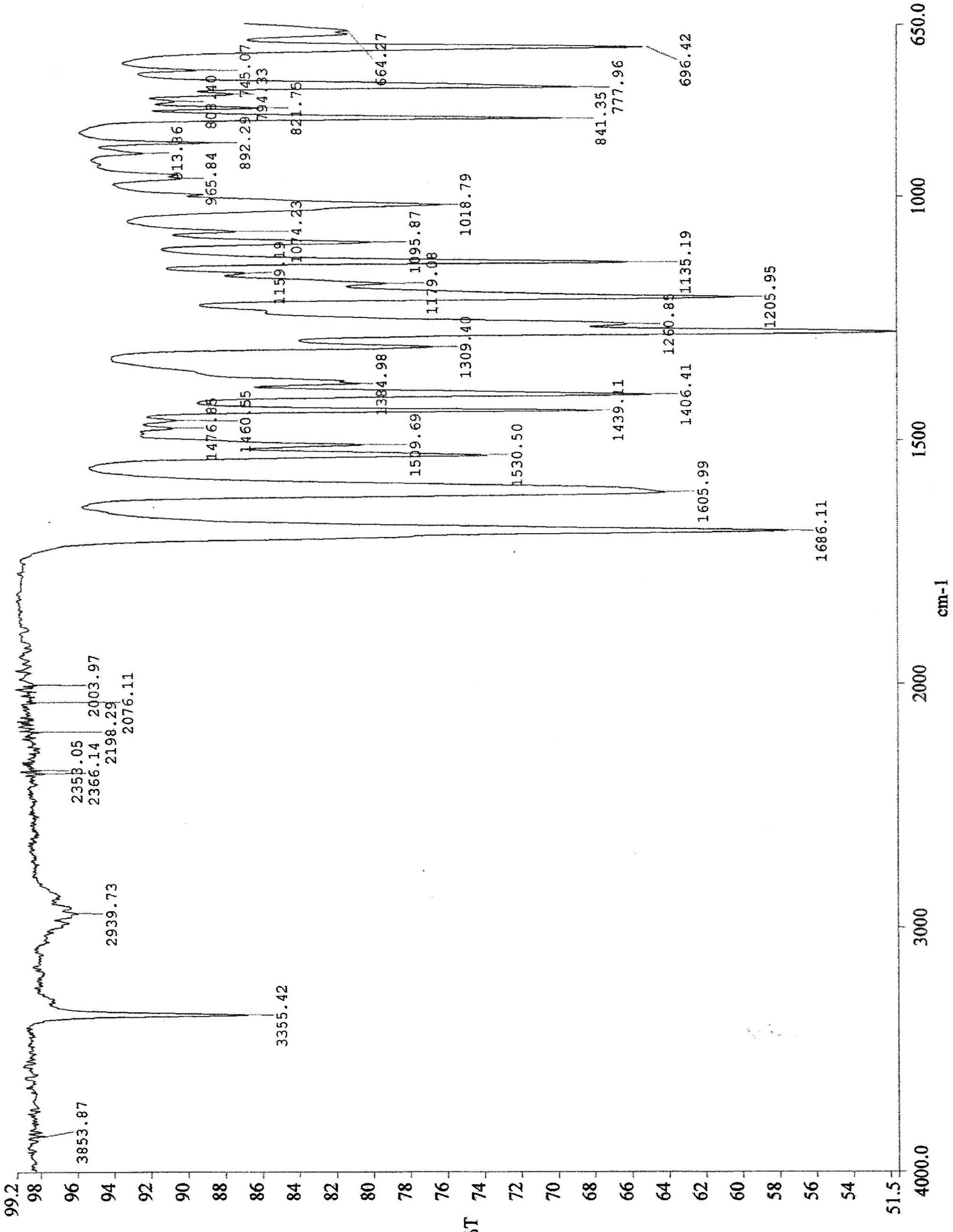

Figure IIh.i FT-IR Spectrum of Compound IIh
Methyl 2-(3-methylbenzyloxy)-4-propionamido salicylate, $C_{19}H_{21}NO_4$

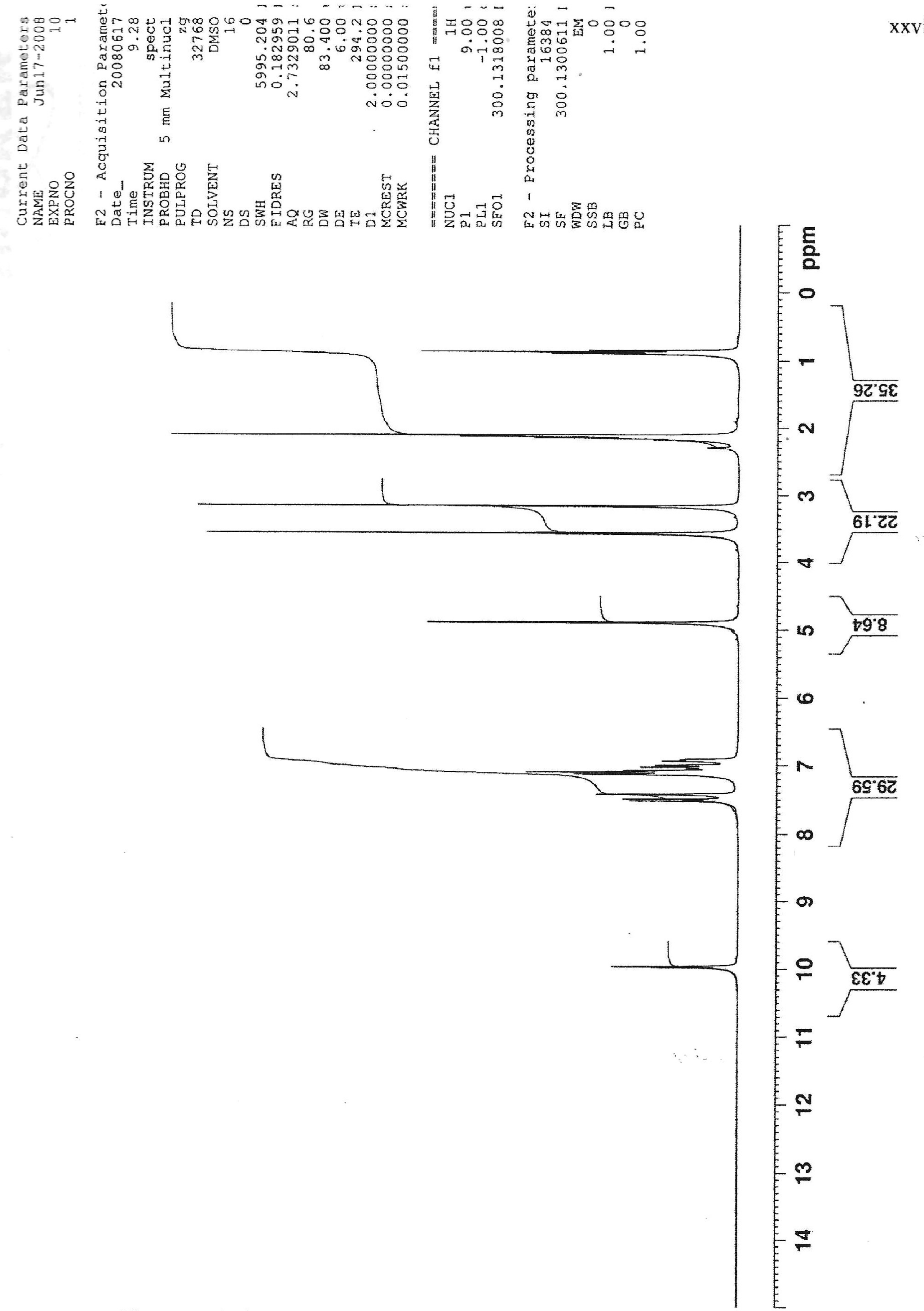

Figure IIh.ii ^{1}H NMR Spectrum of Compound IIh
Methyl 2-(3-methylbenzyloxy)-4-propionamido salicylate, $C_{19}H_{21}NO_4$

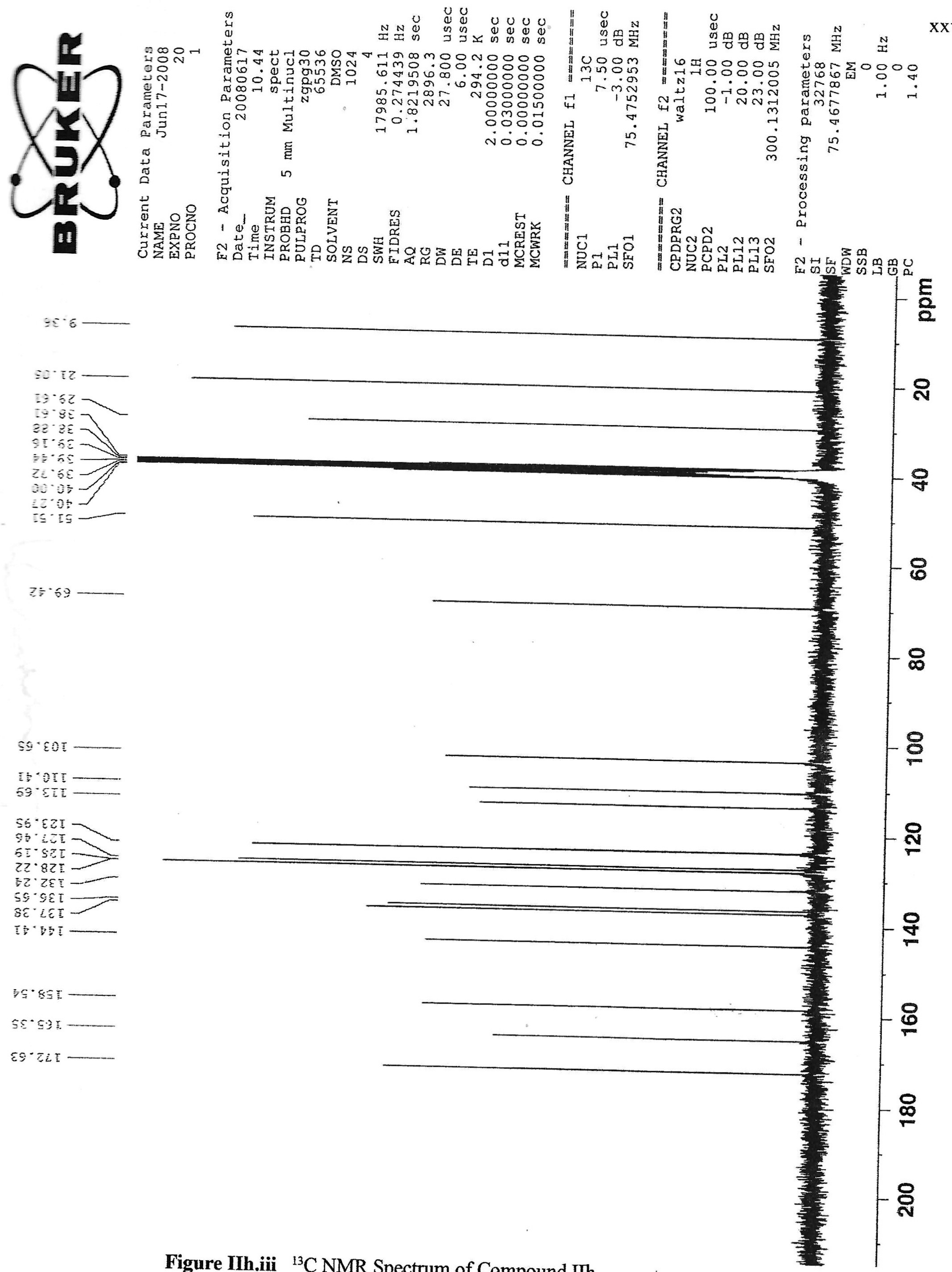

Figure IIh.iii ^{13}C NMR Spectrum of Compound IIh
Methyl 2-(3-methylbenzyloxy)-4-propionamido salicylate, $C_{19}H_{21}NO_4$

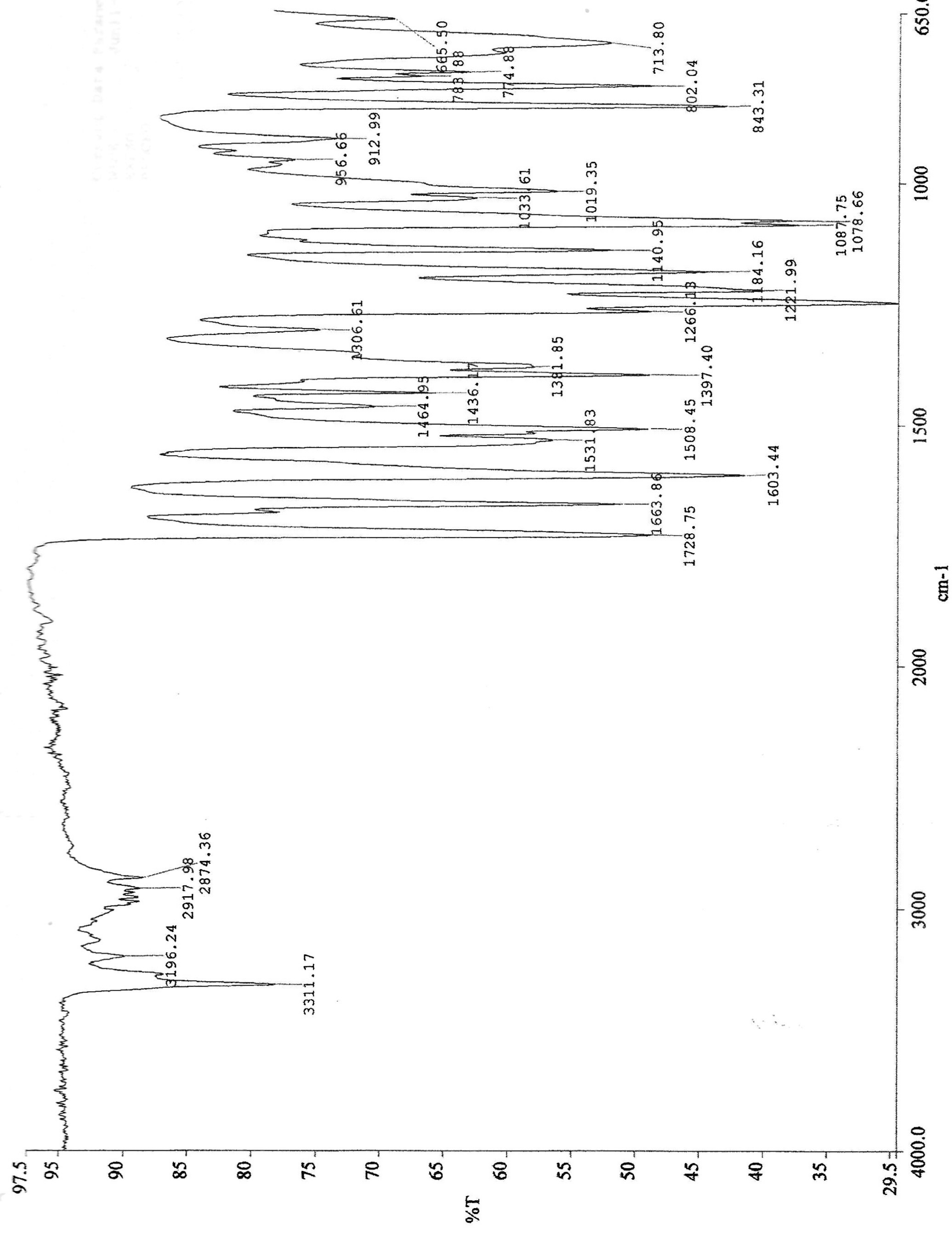

Figure IIi.i FT-IR Spectrum of Compound IIi
Methyl 2-(4-methylbenzyloxy)-4-propionamido salicylate, $C_{19}H_{21}NO_4$

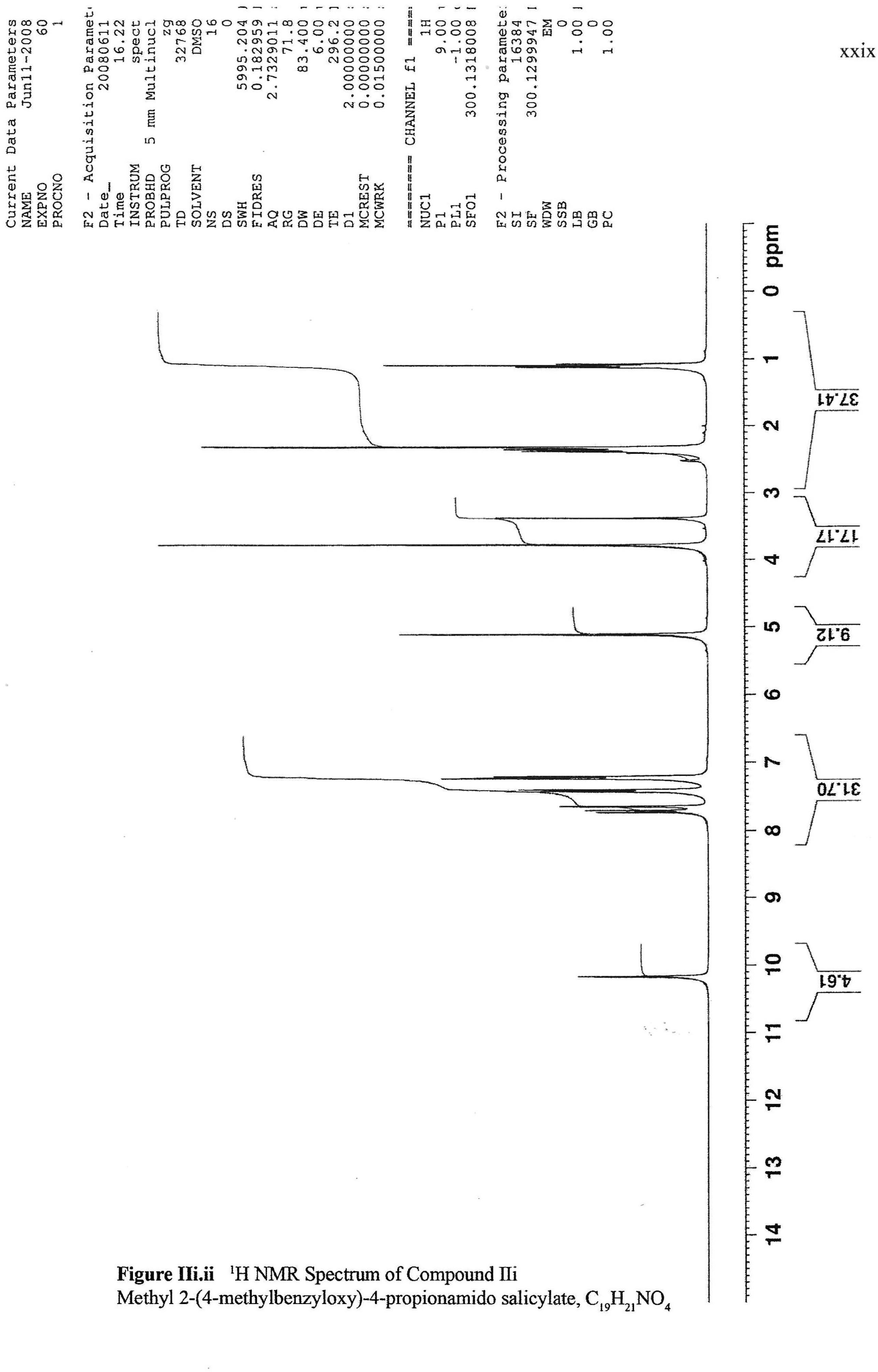

Figure IIi.ii ¹H NMR Spectrum of Compound IIi
Methyl 2-(4-methylbenzyloxy)-4-propionamido salicylate, $C_{19}H_{21}NO_4$

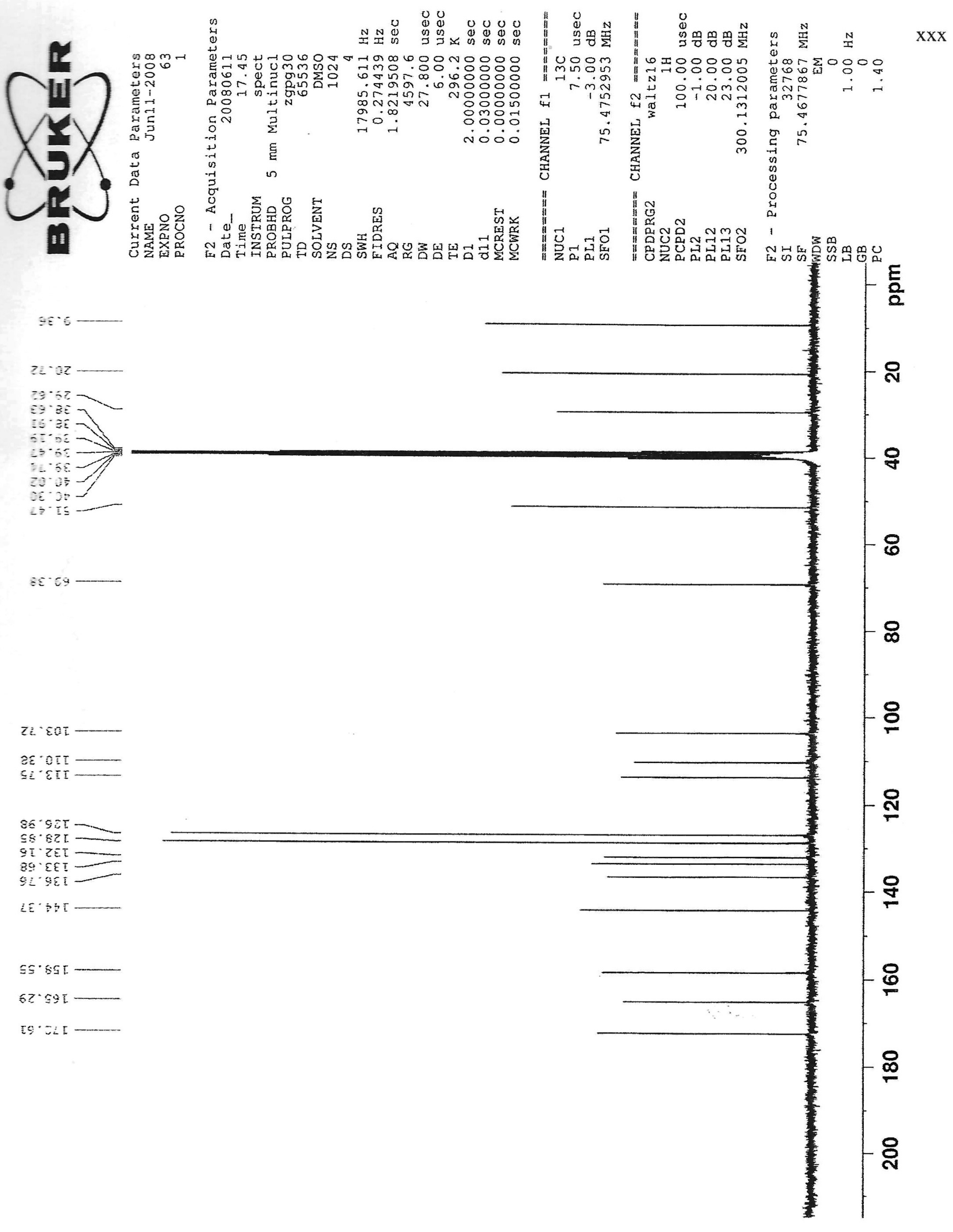

Figure IIi.iii ^{13}C NMR Spectrum of Compound IIi
Methyl 2-(4-methylbenzyloxy)-4-propionamido salicylate, $C_{19}H_{21}NO_4$

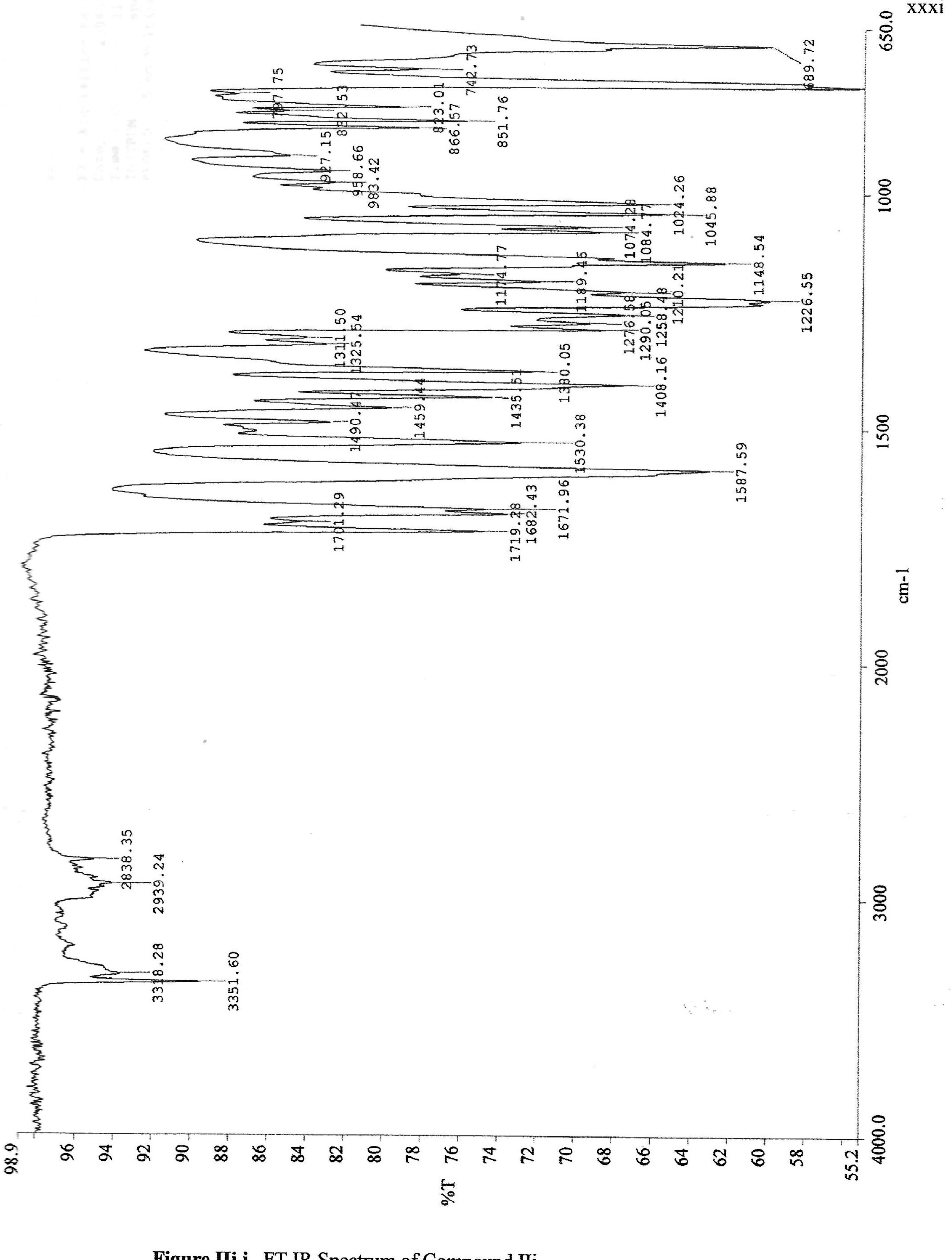

Figure IIj.i FT-IR Spectrum of Compound IIj
Methyl 2-(3-methoxybenzyloxy)-4-propionamido salicylate, $C_{19}H_{21}NO_5$

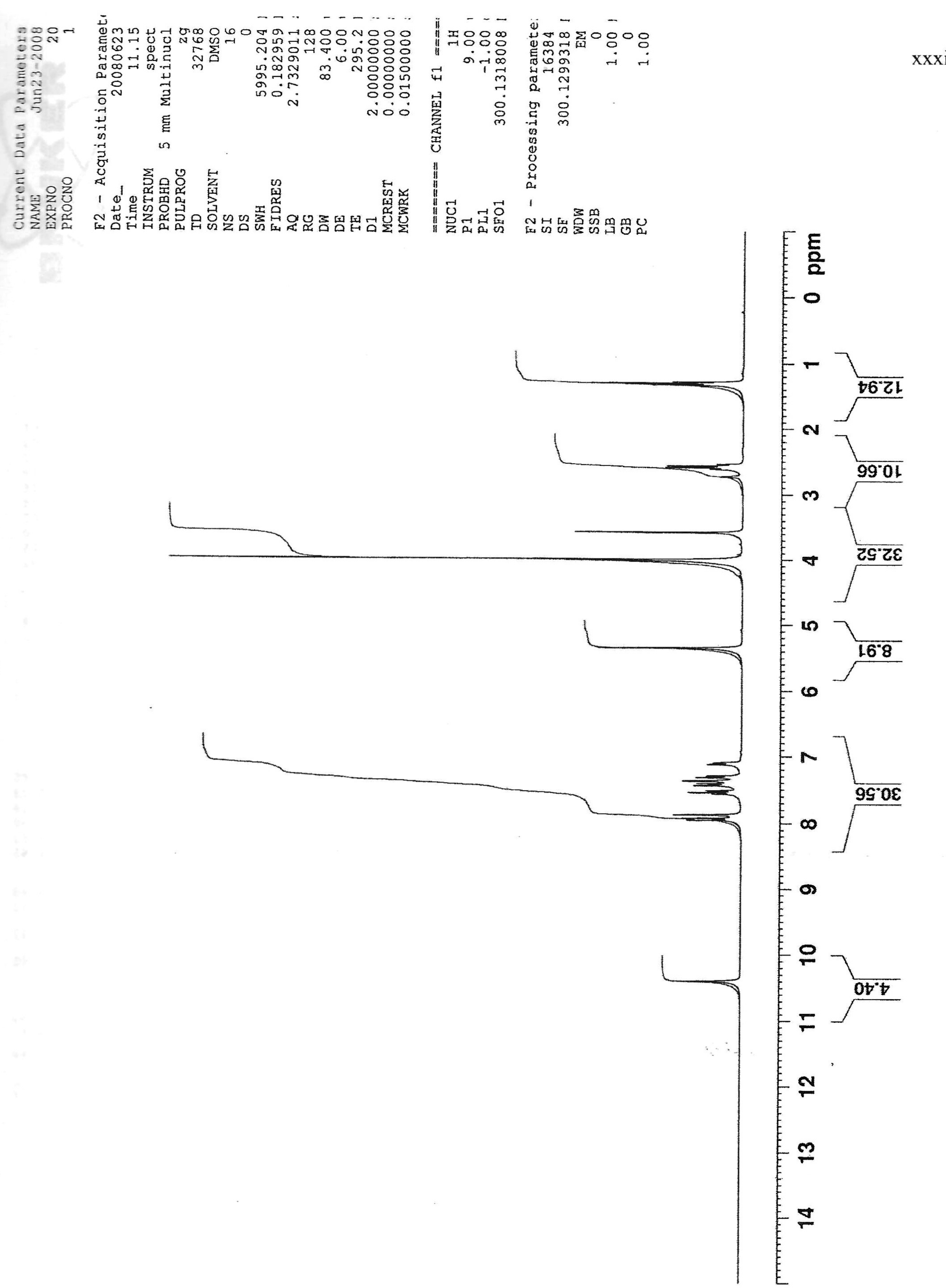

Figure IIj.ii ^{1}H NMR Spectrum of Compound IIj
Methyl 2-(3-methoxybenzyloxy)-4-propionamido salicylate, $C_{19}H_{21}NO_5$

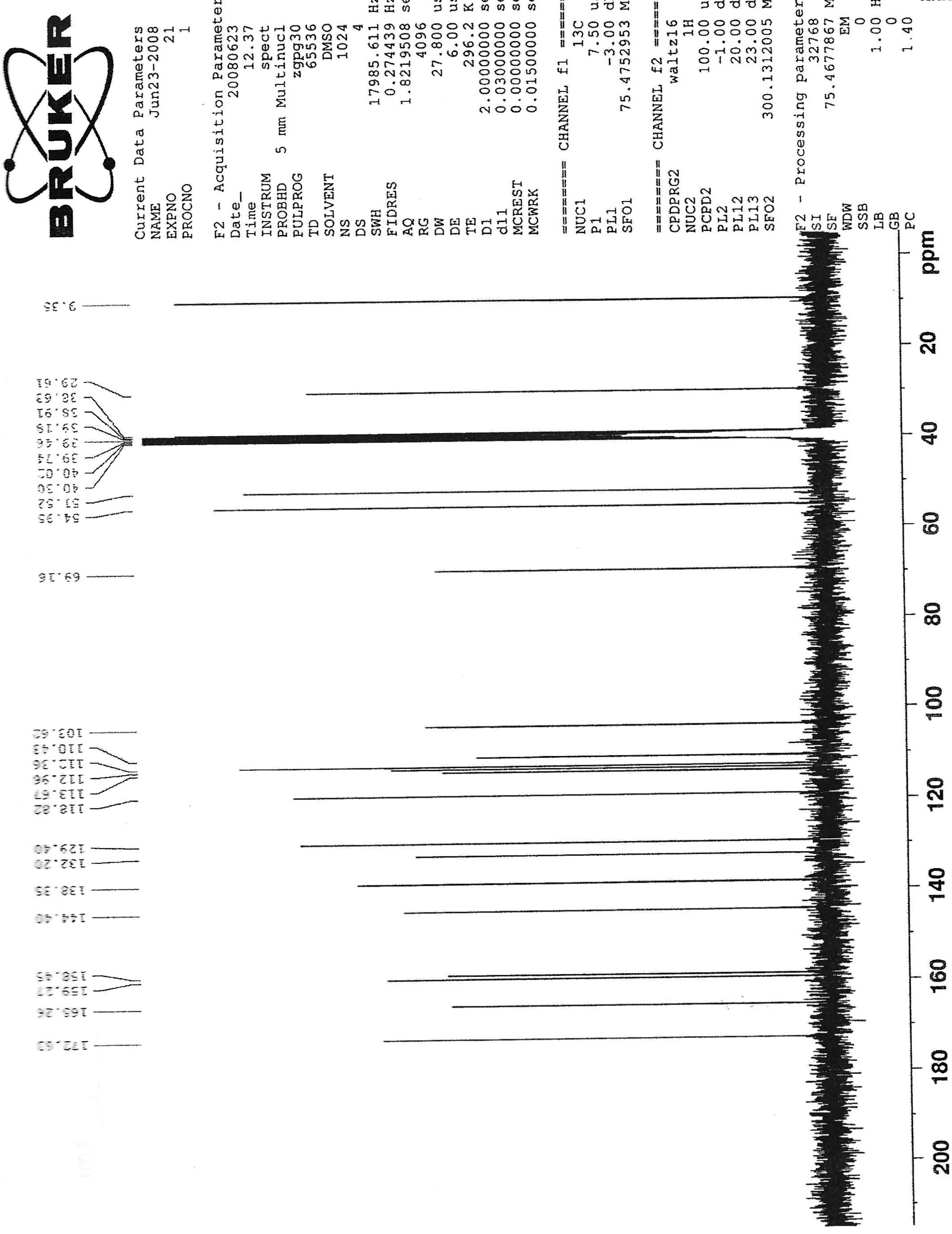

Figure IIj.iii ^{13}C NMR Spectrum of Compound IIj
Methyl 2-(3-methoxybenzyloxy)-4-propionamido salicylate, $C_{19}H_{21}NO_5$

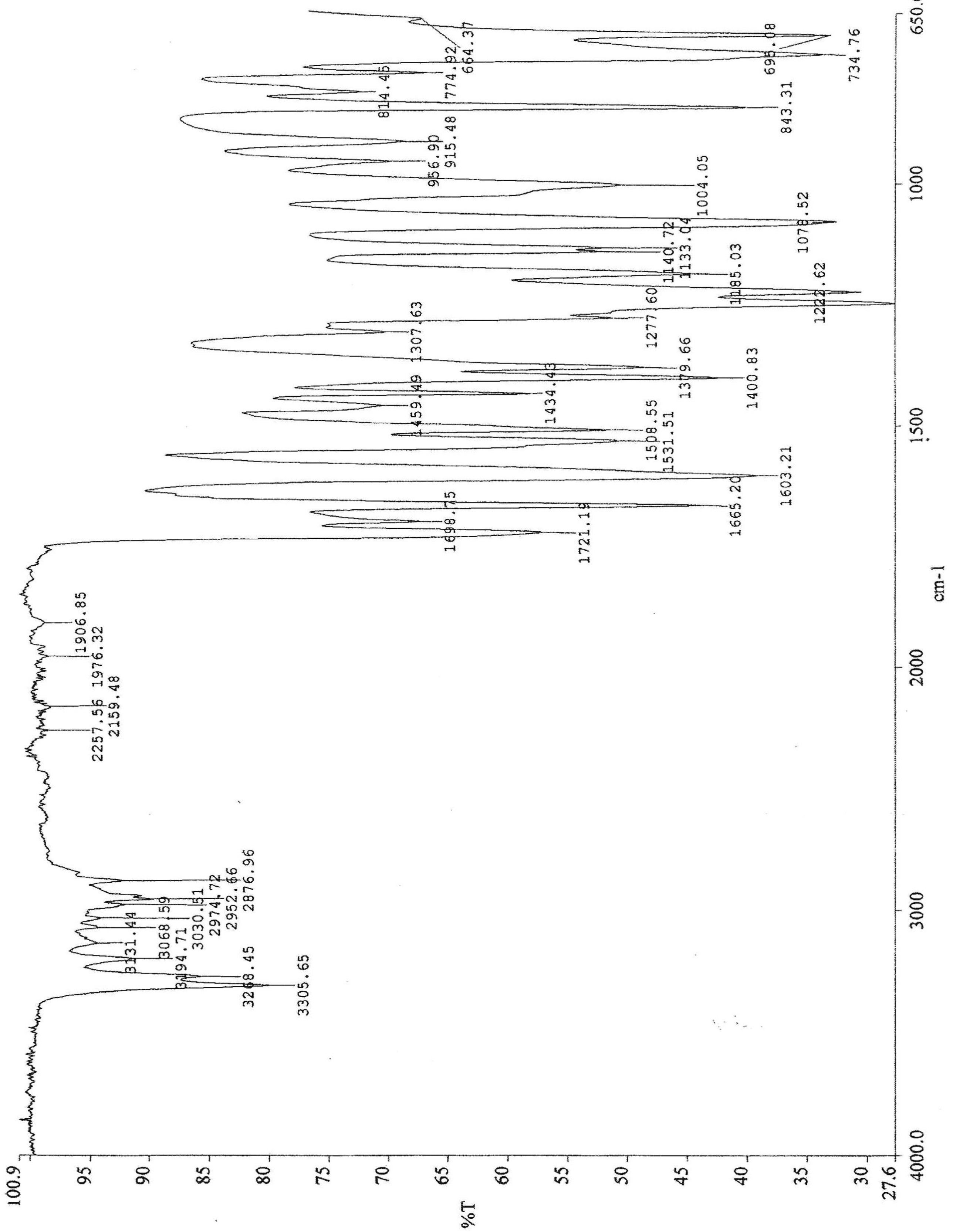

Figure IIk.i FT-IR Spectrum of Compound IIk
Methyl 2-(benzyloxy)-4-propionamido salicylate, $C_{18}H_{19}NO_4$

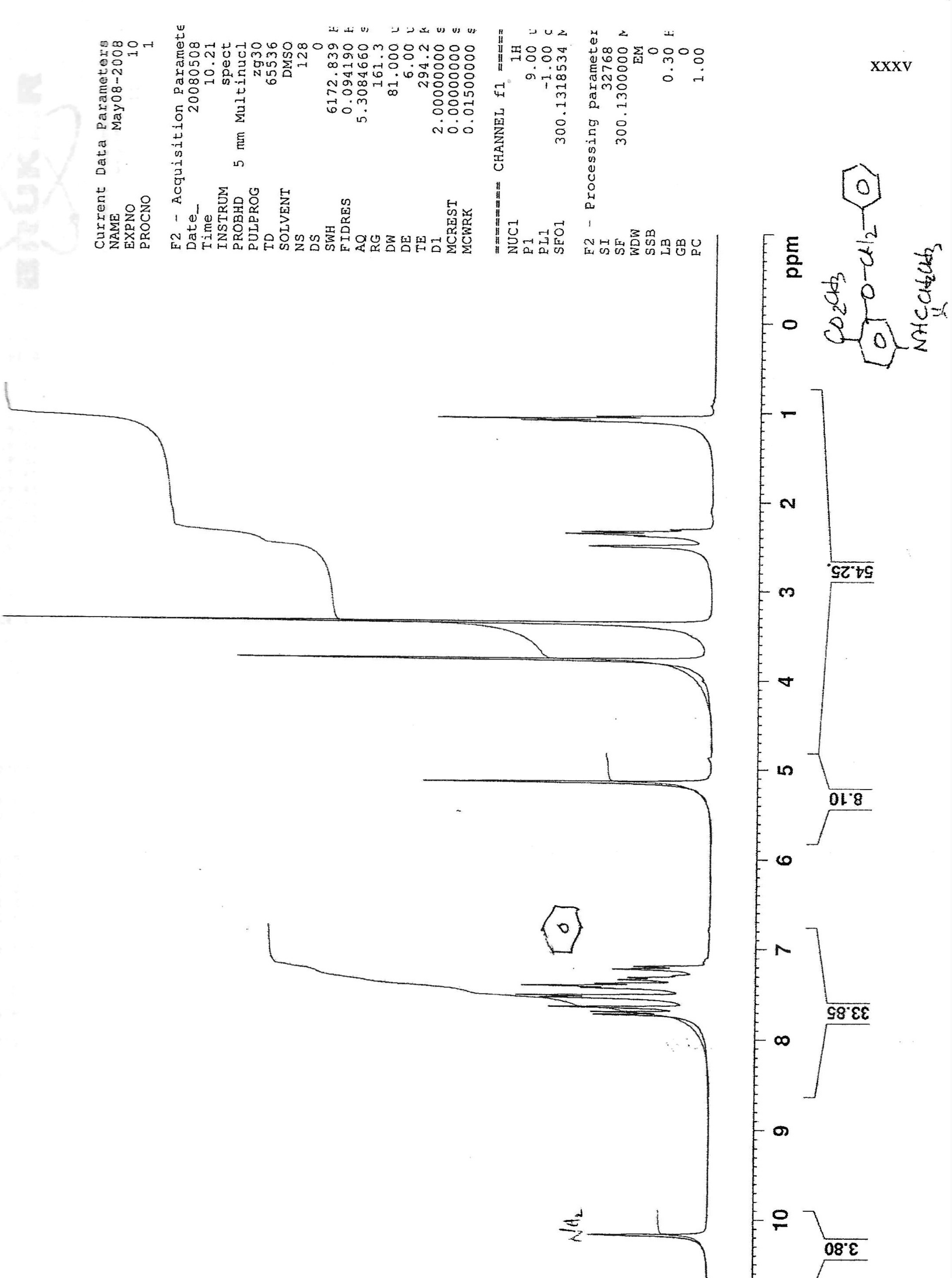

Figure IIk.ii ^{1}H NMR Spectrum of Compound IIk
Methyl 2-(benzyloxy)-4-propionamido salicylate, $C_{18}H_{19}NO_4$

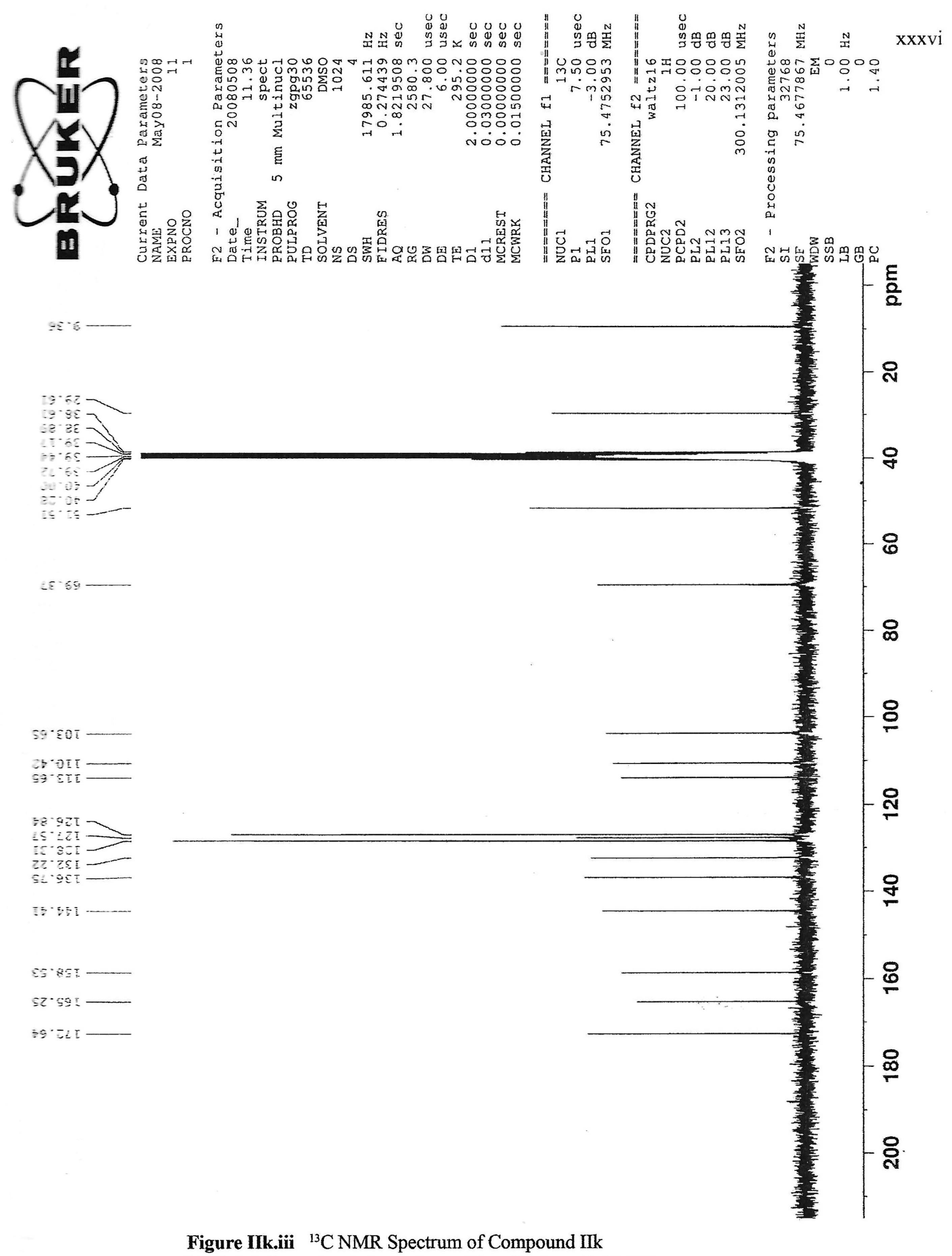

Figure IIk.iii ^{13}C NMR Spectrum of Compound IIk
Methyl 2-(benzyloxy)-4-propionamido salicylate, $C_{18}H_{19}NO_4$

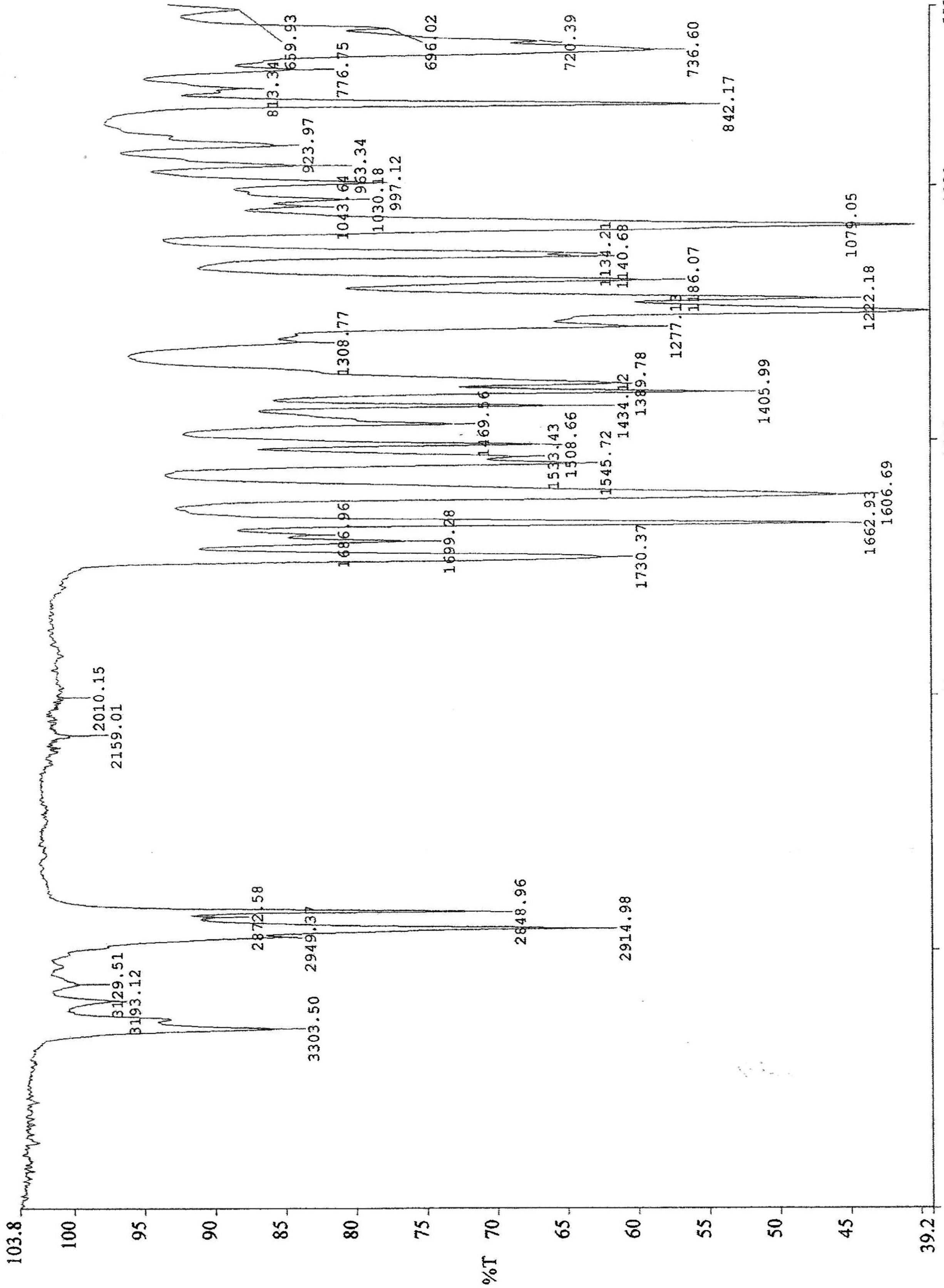

Figure III.i FT-IR Spectrum of Compound III
Methyl 2-(dodecyloxy)-4-propionamido salicylate, $C_{23}H_{37}NO_4$

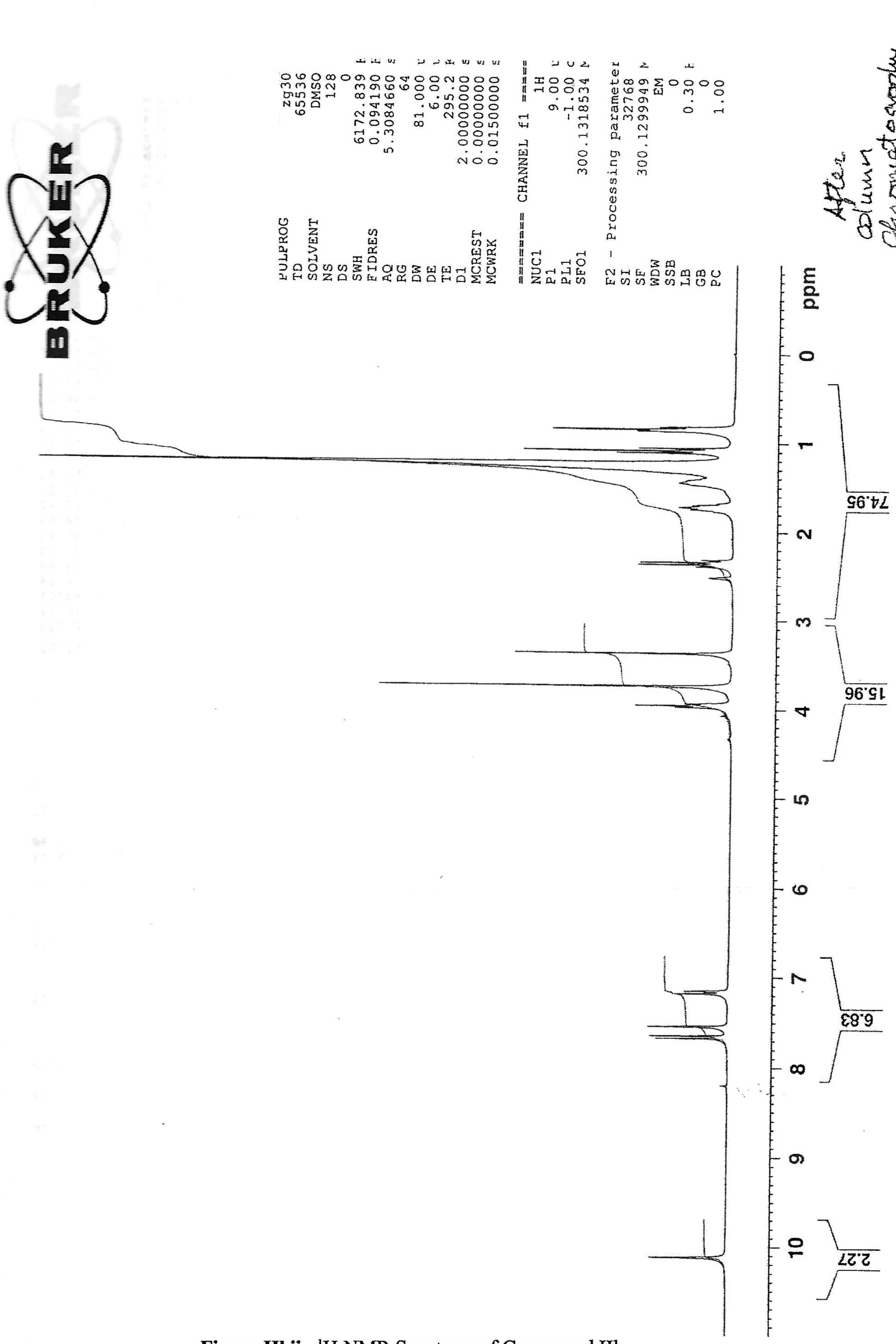

After column Chromatography Sample Actually Shipped

Figure III.ii ^{1}H NMR Spectrum of Compound III
Methyl 2-(dodecyloxy)-4-propionamido salicylate, $C_{23}H_{37}NO_4$

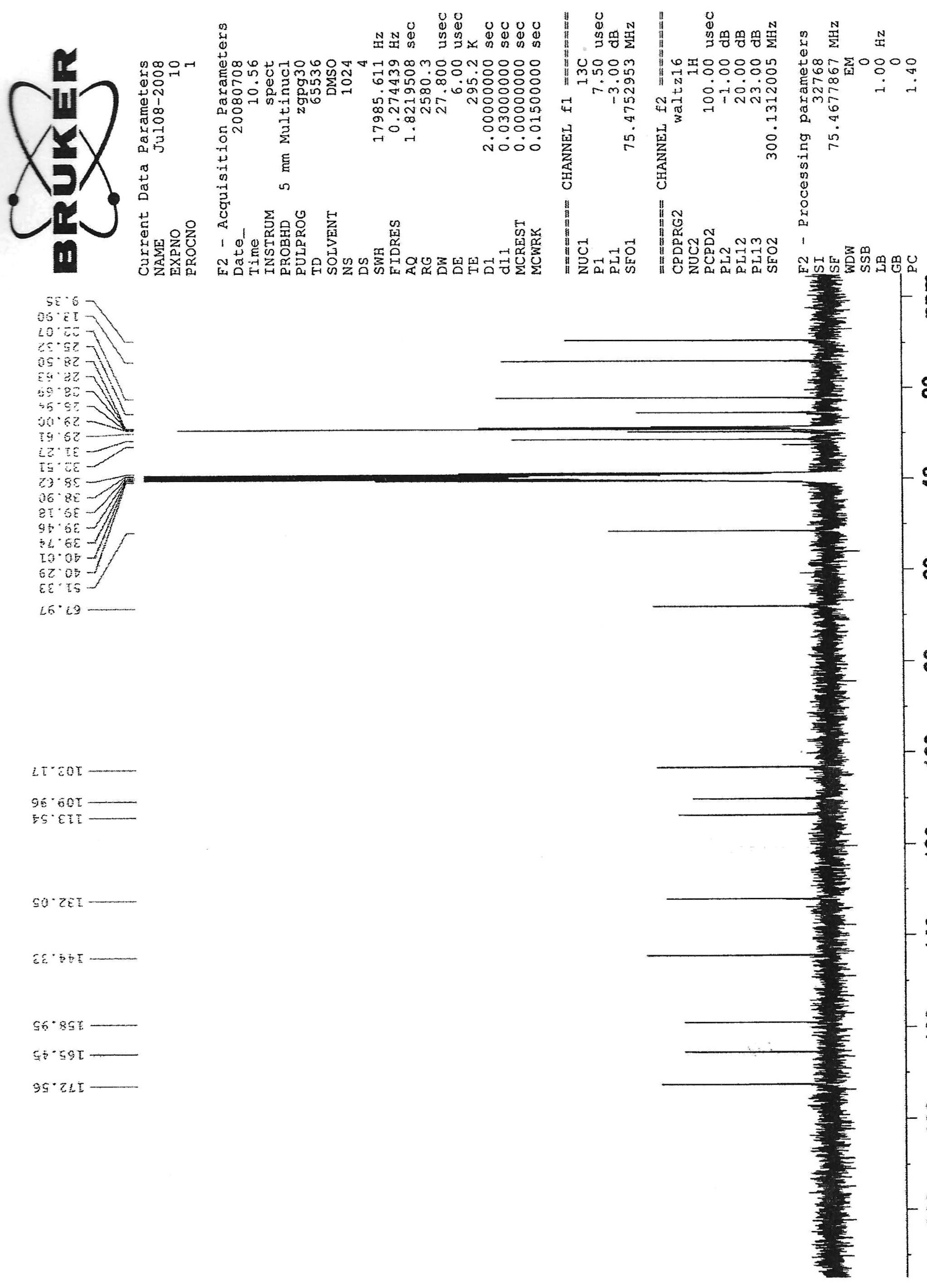

Figure III.iii ^{13}C NMR Spectrum of Compound IIl
Methyl 2-(dodecyloxy)-4-propionamido salicylate, $C_{23}H_{37}NO_4$

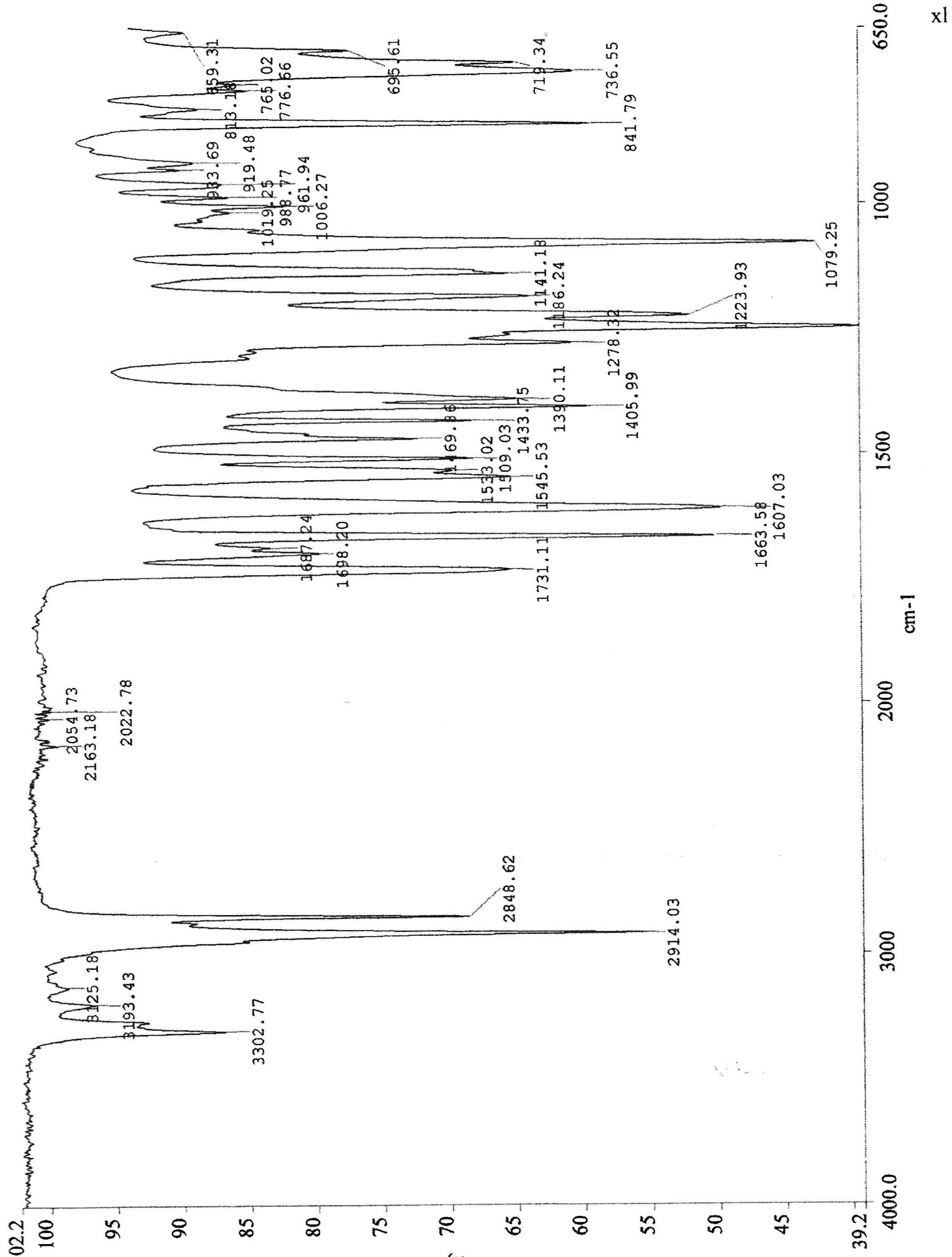

Figure IIm.i FT-IR Spectrum of Compound IIm
Methyl 2-(tetradecanyloxy)-4-propionamido salicylate, $C_{25}H_{41}NO_4$

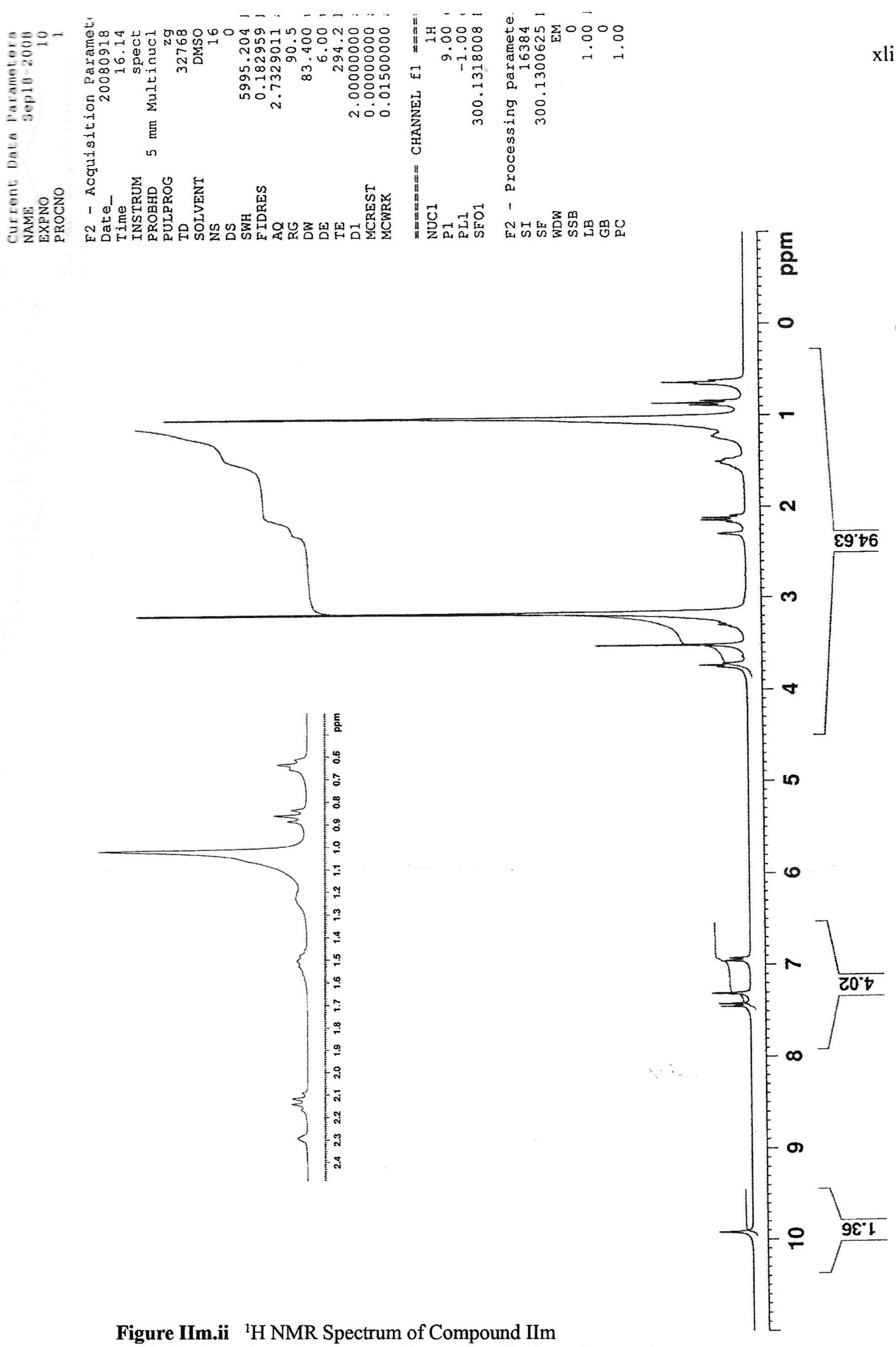

Figure IIm.ii ^{1}H NMR Spectrum of Compound IIm
Methyl 2-(tetradecanyloxy)-4-propionamido salicylate, $C_{25}H_{41}NO_4$

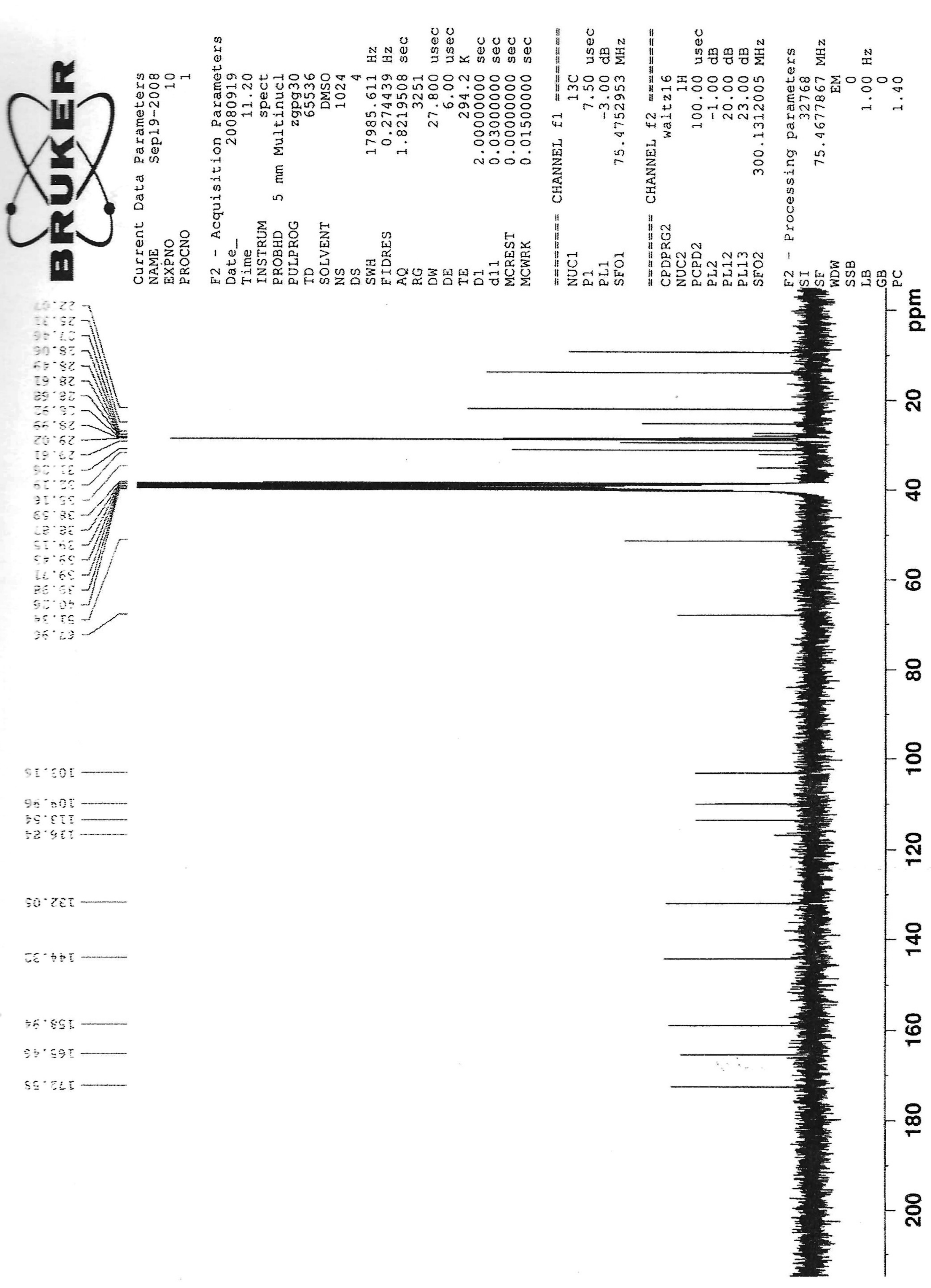

Figure IIm.ii ^{1}H NMR Spectrum of Compound IIm
Methyl 2-(tetradecanyloxy)-4-propionamido salicylate, $C_{25}H_{41}NO_4$

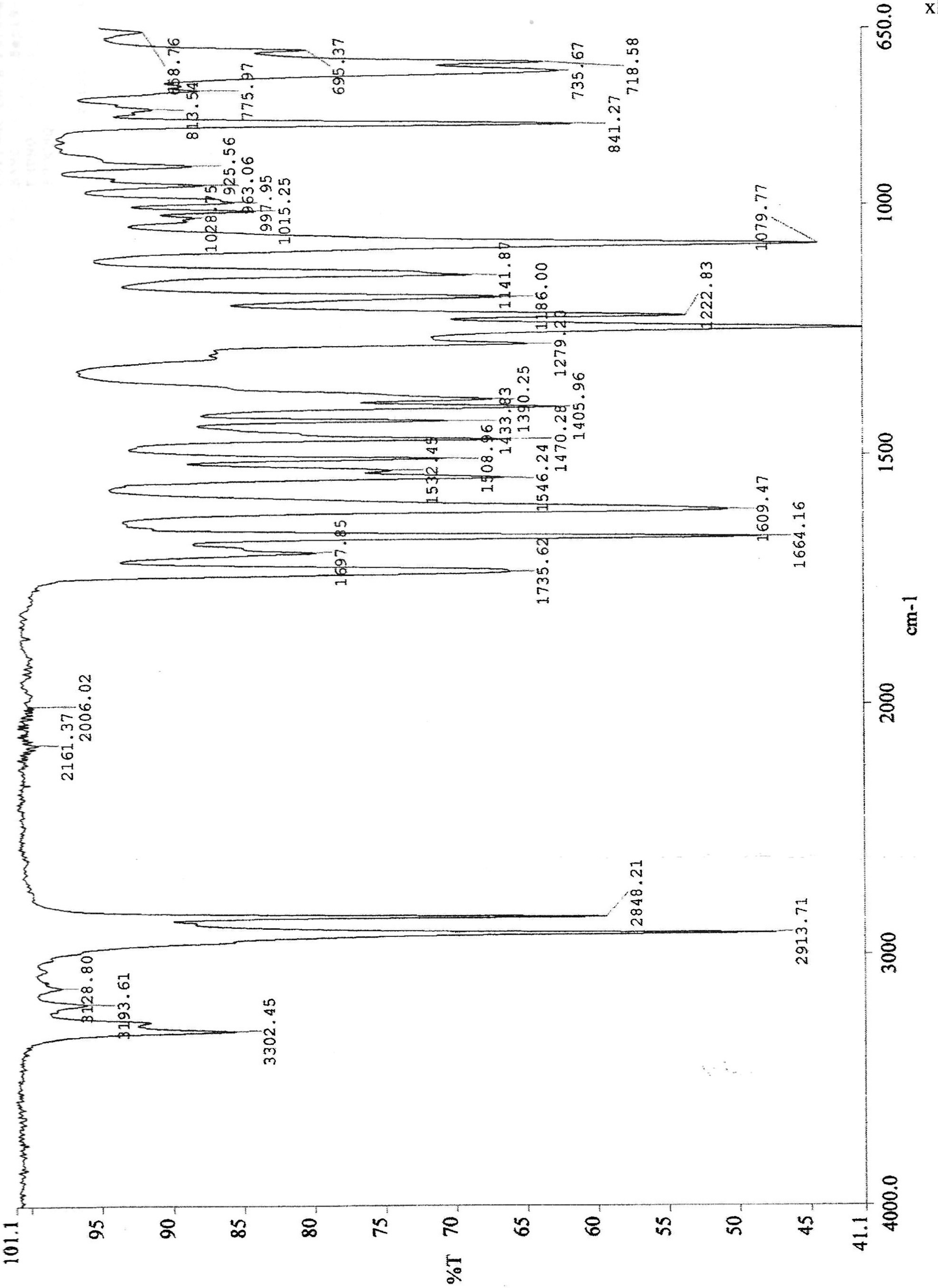

Figure IIn.i FT-IR Spectrum of Compound IIn
Methyl 2-(hexadecanyloxy)-4-propionamido salicylate, $C_{27}H_{45}NO_4$

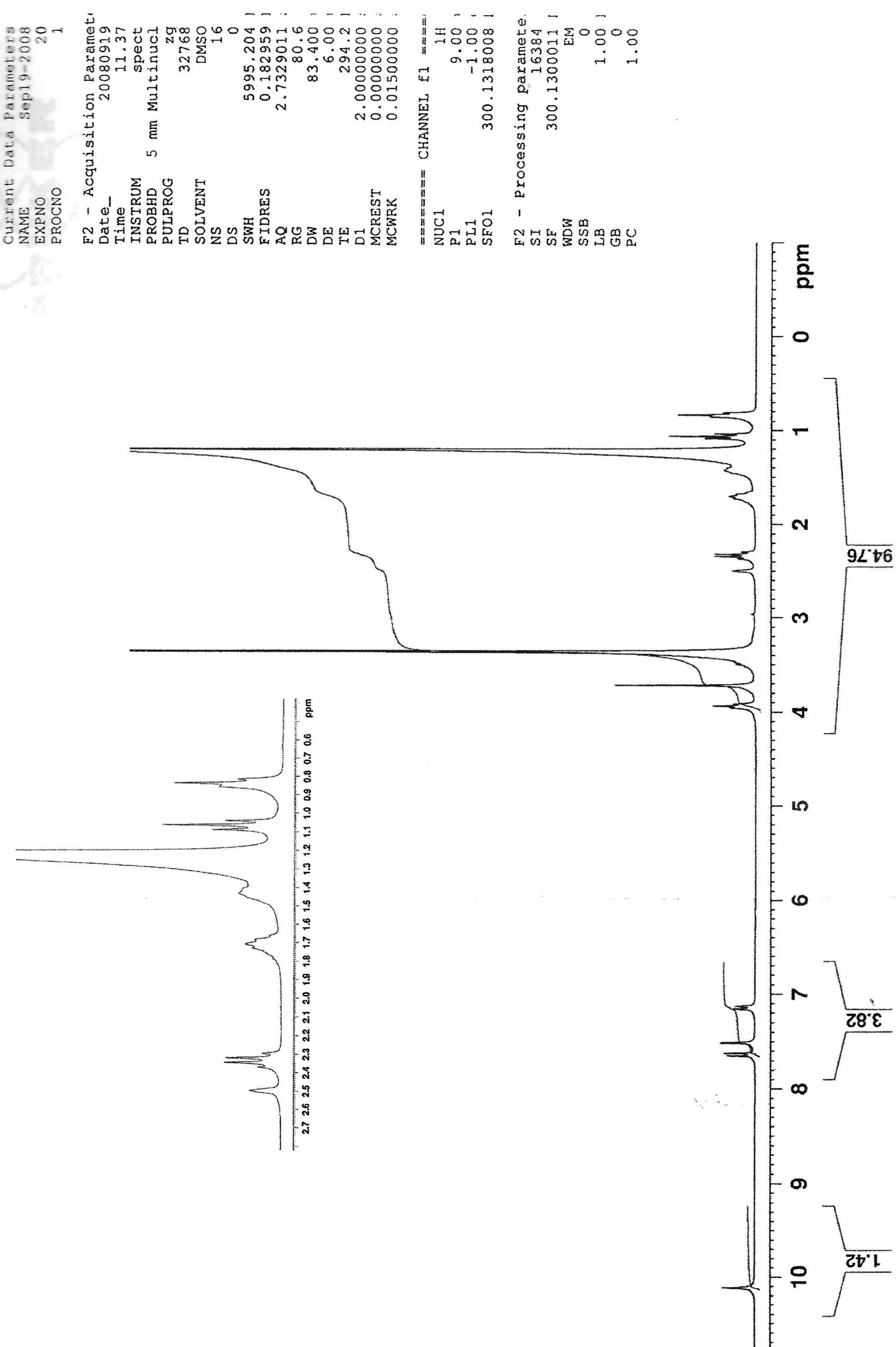

Figure IIn.ii ^{1}H NMR Spectrum of Compound IIn
Methyl 2-(hexadecanyloxy)-4-propionamido salicylate, $C_{27}H_{45}NO_4$

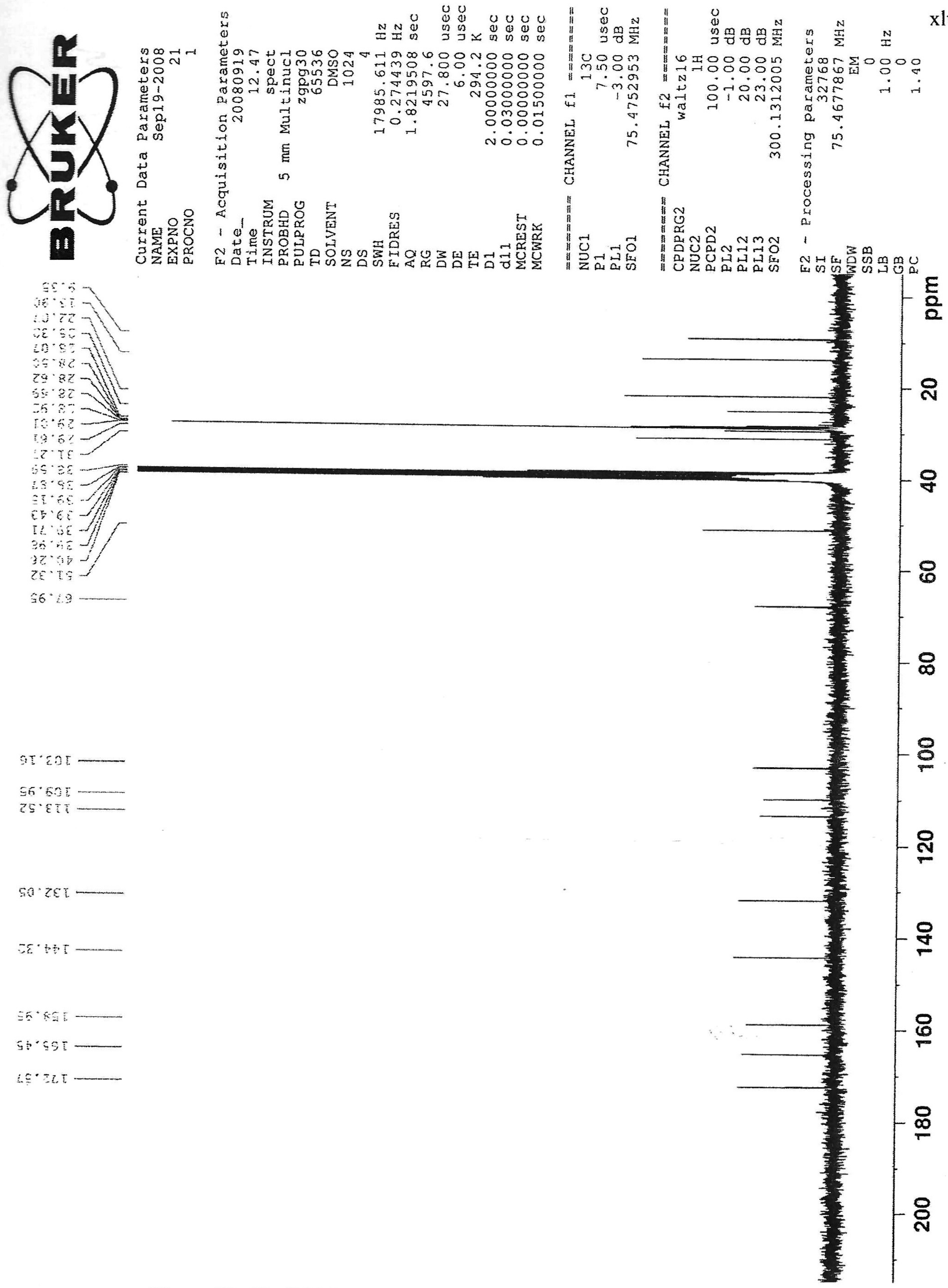

Figure IIn.iii ^{13}C NMR Spectrum of Compound IIn
Methyl 2-(hexadecanyloxy)-4-propionamido salicylate, $C_{27}H_{45}NO_4$

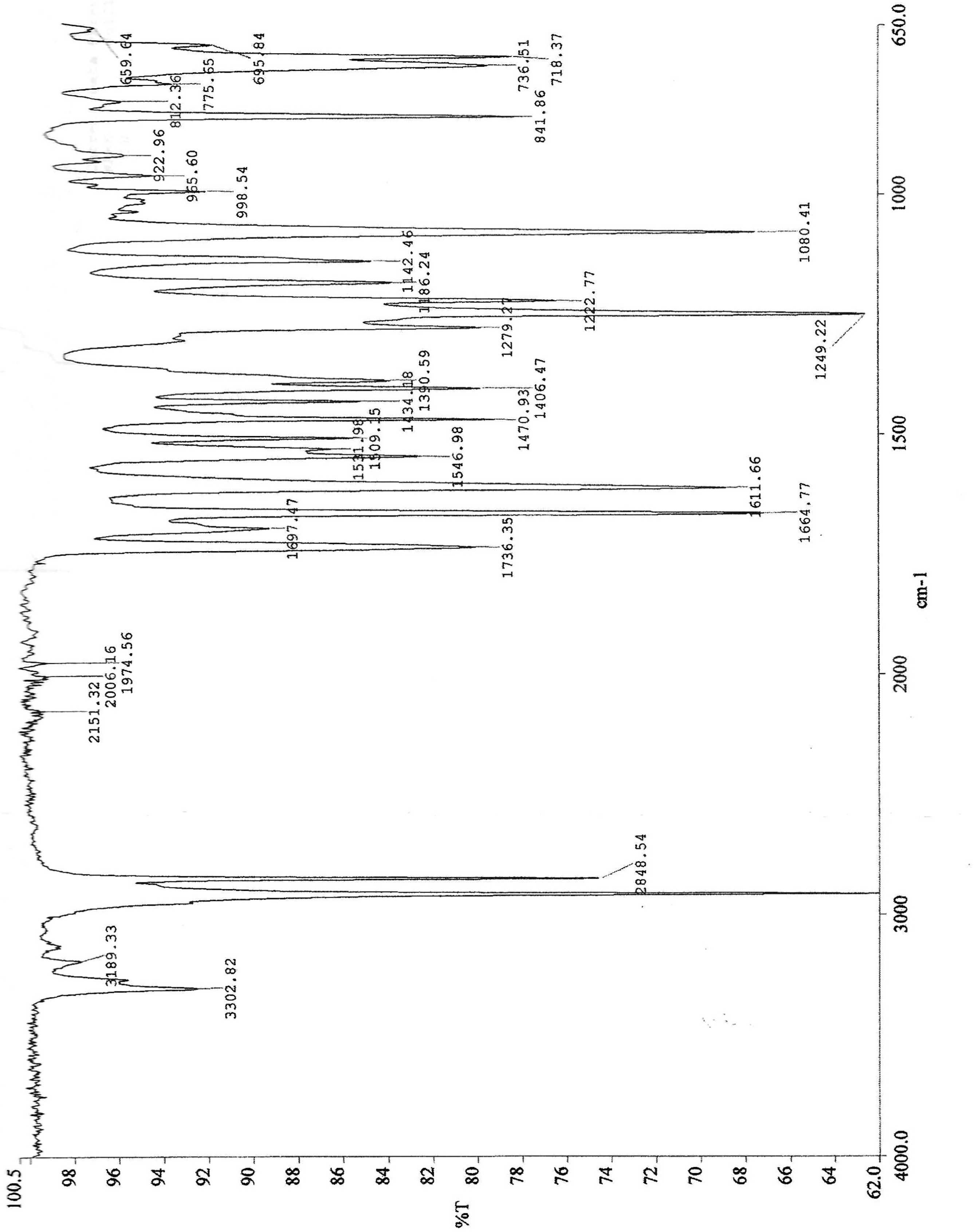

Figure IIo.i FT-IR Spectrum of Compound IIo
Methyl 2-(octadecanyloxy)-4-propionamido salicylate, $C_{29}H_{49}NO_4$

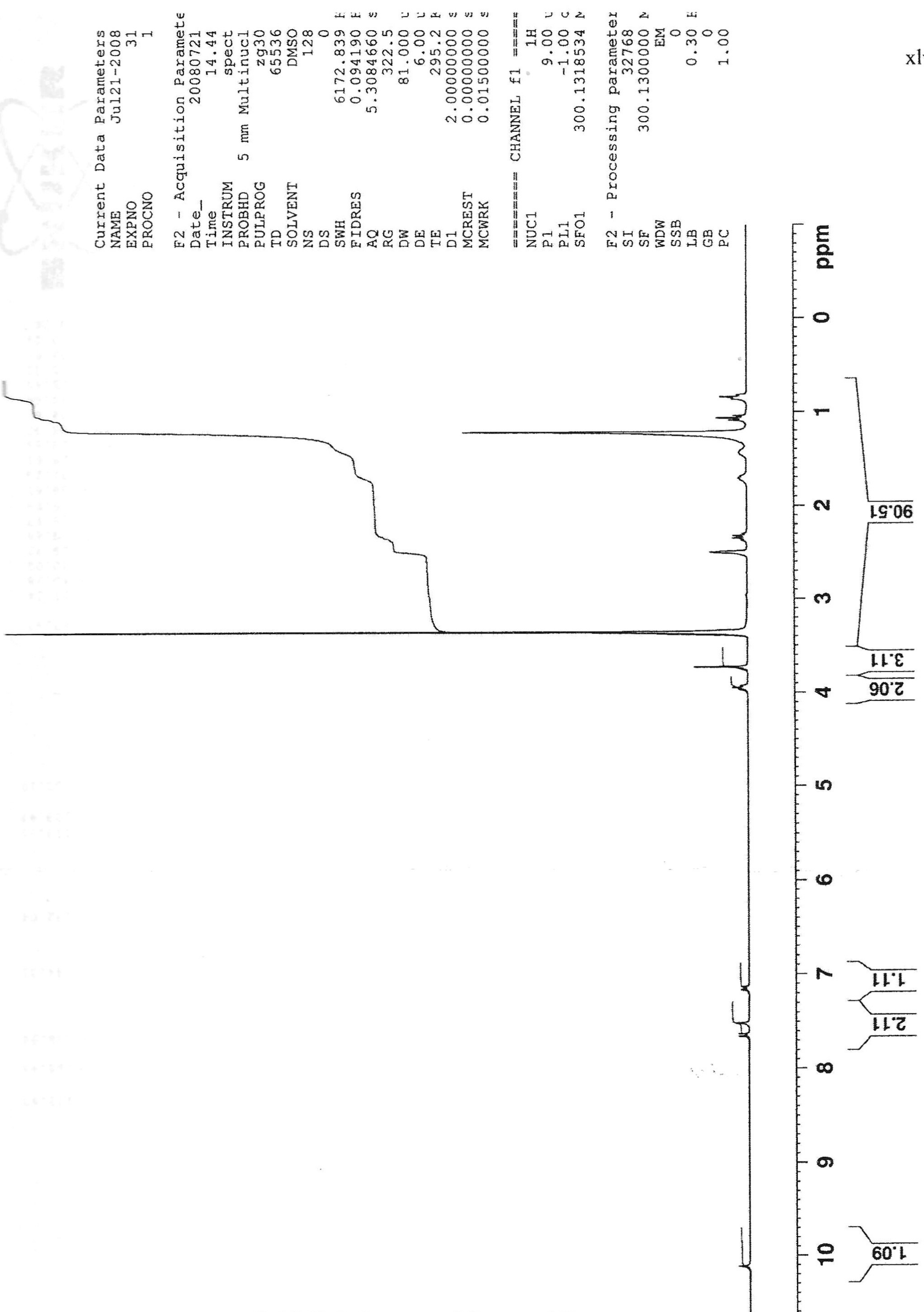

Figure IIo.ii ^{1}H NMR Spectrum of Compound IIo
Methyl 2-(octadecanyloxy)-4-propionamido salicylate, $C_{29}H_{49}NO_4$

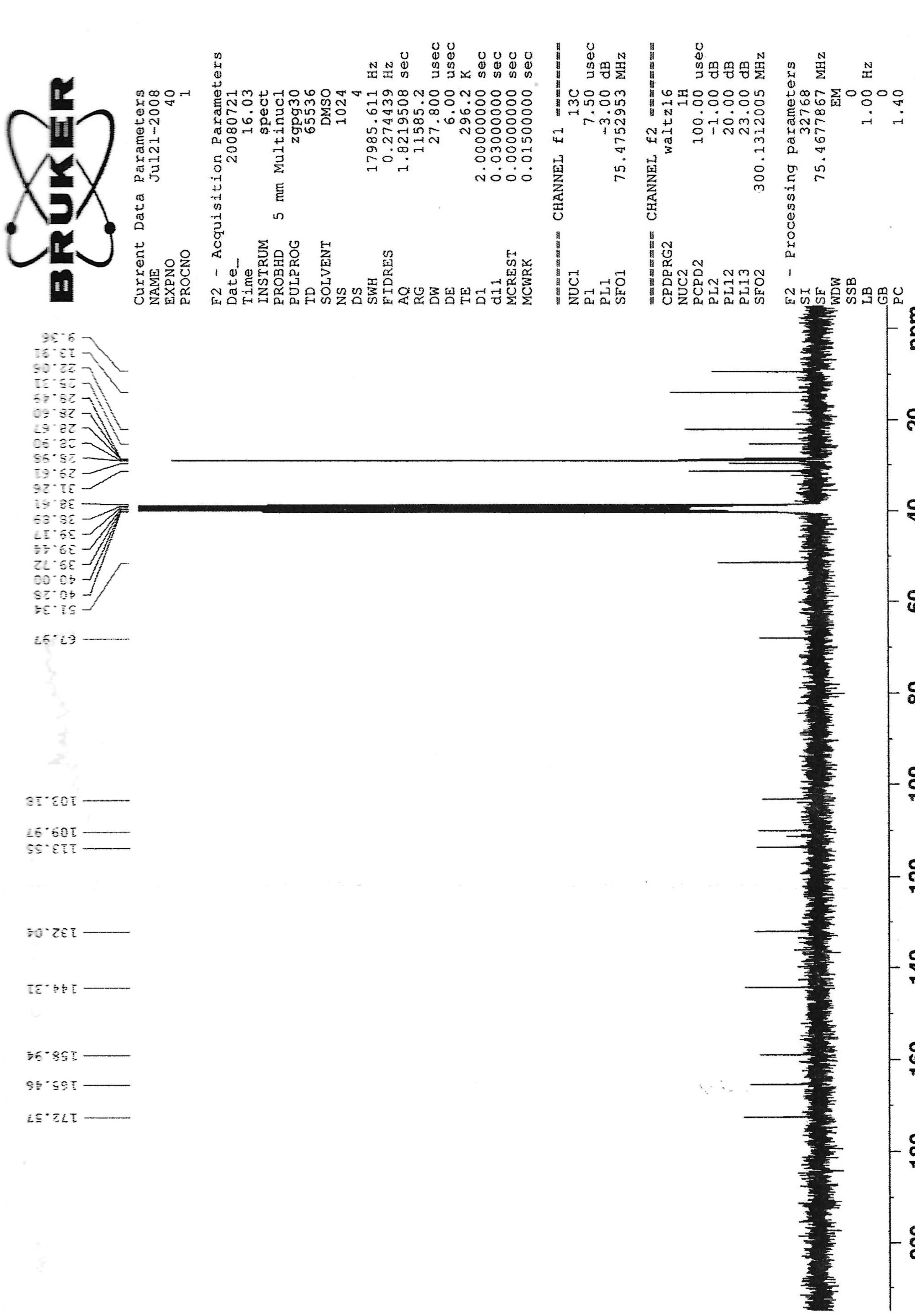

Figure IIo.iii ^{13}C NMR Spectrum of Compound IIo
Methyl 2-(octadecanyloxy)-4-propionamido salicylate, $C_{29}H_{49}NO_4$
