# Supplementary material for: Preparation of Lipophilic Derivatives of para-Aminosalicylic Acid for Antimicrobial Drug Design
Source: ACS Omega. 2025 Sep 24;10(39):46134–40. doi: 10.1021/acsomega.5c08092 (PMC12508941; doi:10.1021/acsomega.5c08092)
Supplement: Supplementary file 2 [file ao5c08092_si_002.pdf]

Preparation of Lipophilic Derivatives of *para*-Aminosalicylic Acid for Antimicrobial Drug Design

Michael J. Hearn* and Alice K. Min

Department of Chemistry
Wellesley College
Wellesley, Massachusetts 02481 USA

*Corresponding Author; E-Mail: MHearn@Wellesley.edu

Supplementary Materials

Table S1. ^{13}C-NMR chemical shifts of compounds IIa-IIo

Tabulation of Significant Functional Group Similarities

Table S1. ^{13}C-NMR chemical shifts of compounds IIa-IIo[a]

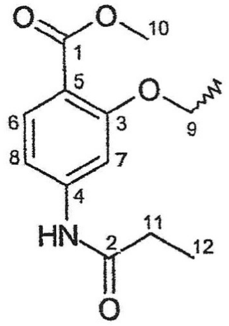

	δ ^{13}C (ppm)											
Compound	C_1	C_2	C_3	C_4	C_5	C_6	C_7	C_8	C_9	C_{10}	C_{11}	C_{12}
**IIa**	173	165	160	144	133	114	111	104	69	52	30	9
**IIb**	173	165	159	144	134	114	111	104	70	52	30	9
**IIc**	173	165	159	144	132	114	110	103	68	51	31	9
**IId**	173	166	159	145	132	114	110	104	68	52	31	10
**IIe**	173	165	159	144	133	114	110	103	68	51	30	9
**IIf**	173	165	159	144	132	114	110	103	68	51	31	9
**IIg**	173	165	159	144	132	114	110	103	66	51	30	9
**IIh**	173	166	159	144	132	114	110	104	69	52	30	9
**IIi**	173	165	159	144	132	116	110	104	69	51	30	9
**IIj**	173	165	159	144	132	114	110	104	69	52	30	9
**IIk**	173	165	159	144	132	114	110	104	69	52	30	9
**IIl**	173	165	159	144	132	114	110	103	68	51	30	9
**IIm**	173	165	159	144	132	114	110	103	68	51	30	-
**IIn**	173	165	159	144	132	114	110	103	68	51	31	9
**IIo**	173	165	159	144	132	114	110	103	68	51	31	9

[a] Complete spectral data for each compound are given in the Experimental section and copies of the original spectra are provided in the Supplementary Information.
